# A century of exercise physiology: effects of muscle contraction and exercise on skeletal muscle Na^+^,K^+^-ATPase, Na^+^ and K^+^ ions, and on plasma K^+^ concentration—historical developments

**DOI:** 10.1007/s00421-023-05335-9

**Published:** 2024-01-11

**Authors:** Michael J. McKenna, Jean-Marc Renaud, Niels Ørtenblad, Kristian Overgaard

**Affiliations:** 1 Institute for Health and Sport, Victoria University, Melbourne, VIC 8001 Australia; 2 College of Physical Education, Southwest University, Chongqing, China; 3 College of Sport Science, Zhuhai College of Science and Technology, Zhuhai, China; 4 Department of Cellular and Molecular Medicine, Neuromuscular Research Center, University of Ottawa, Ottawa, ON Canada; 5 Department of Sports Science and Clinical Biomechanics, University of Southern Denmark, Odense, Denmark; 6 Exercise Biology, Department of Public Health, Aarhus University, Aarhus, Denmark

**Keywords:** Skeletal muscle, Plasma, Potassium, Sodium, Exercise, Fatigue, FXYD, Na^+^, K^+^-pump

## Abstract

This historical review traces key discoveries regarding K^+^ and Na^+^ ions in skeletal muscle at rest and with exercise, including contents and concentrations, Na^+^,K^+^-ATPase (NKA) and exercise effects on plasma [K^+^] in humans. Following initial measures in 1896 of muscle contents in various species, including humans, electrical stimulation of animal muscle showed K^+^ loss and gains in Na^+^, Cl^−^ and H_2_0, then subsequently bidirectional muscle K^+^ and Na^+^ fluxes. After NKA discovery in 1957, methods were developed to quantify muscle NKA activity via rates of ATP hydrolysis, Na^+^/K^+^ radioisotope fluxes, [^3^H]-ouabain binding and phosphatase activity. Since then, it became clear that NKA plays a central role in Na^+^/K^+^ homeostasis and that NKA content and activity are regulated by muscle contractions and numerous hormones. During intense exercise in humans, muscle intracellular [K^+^] falls by 21 mM (range − 13 to − 39 mM), interstitial [K^+^] increases to 12–13 mM, and plasma [K^+^] rises to 6–8 mM, whilst post-exercise plasma [K^+^] falls rapidly, reflecting increased muscle NKA activity. Contractions were shown to increase NKA activity in proportion to activation frequency in animal intact muscle preparations. In human muscle, [^3^H]-ouabain-binding content fully quantifies NKA content, whilst the method mainly detects α_2_ isoforms in rats. Acute or chronic exercise affects human muscle K^+^, NKA content, activity, isoforms and phospholemman (FXYD1). Numerous hormones, pharmacological and dietary interventions, altered acid–base or redox states, exercise training and physical inactivity modulate plasma [K^+^] during exercise. Finally, historical research approaches largely excluded female participants and typically used very small sample sizes.

## Introduction and overview of muscle ions, excitability and contraction

**Fig. 1:**
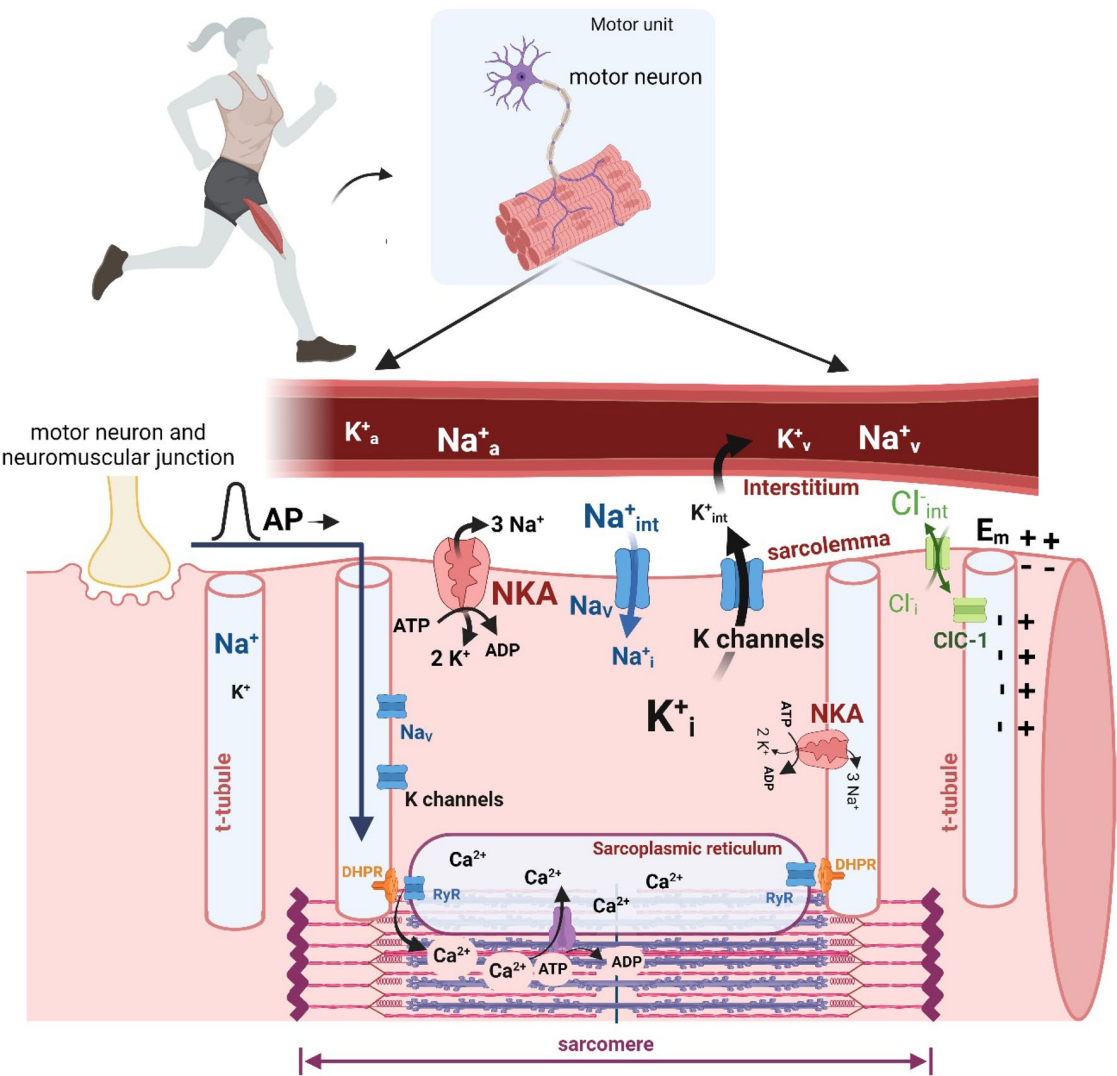
Schematic overview of ion movements in skeletal muscle during excitation contraction coupling. Overview of the sequence of events in excitation-contraction coupling leading to muscle contraction, Na^+^ and K^+^ movements and their regulation. The muscle action potential (AP) is initiated at the neuromuscular junction and transmitted along the sarcolemmal membrane of the muscle and through the transverse tubules (t-tubules) into the interior of the muscle fibre. The t-tubular membrane expresses voltage-gated dihydropyridine receptors (DHPR) which are in close contact with the sarcoplasmic reticulum (SR) Ca^2+^ release channels (RyR). The depolarisation of the DHPRs results in opening of the RyR receptor with an ensuing SR Ca^2+^ release, causing a transient increase in intracellular free [Ca^2+^] permitting the cycling of cross-bridges which eventually results in force development, whilst relaxation is caused by an active pumping of Ca^2+^ back to SR. Ion distribution at rest shows high intracellular [K^+^] and low [Na^+^], with low [K^+^] and high [Na^+^] in the extracellular space (interstitium). These steep trans-membrane concentration gradients for Na^+^ and K^+^ allow for propagation of the AP and contribute to maintenance of membrane potential. The AP is generated by Na^+^ influx via opening of voltage-gated Na^+^ channels followed by K^+^ efflux via voltage sensitive K^+^ channels. During an AP, there is a net K^+^ efflux into the interstitium and Na^+^ enters the cell, with K^+^ returned intracellularly and Na^+^ extruded by the NKA. During contractions, there is a net cellular gain of Na^+^ and loss of K^+^ from the fibre, with K^+^ then diffusing from the interstitium into capillaries and is removed by the venous circulation. Ca^2+^, calcium; Na^+^, sodium; K^+^, potassium; K^+^_a_, K^+^_v_, K^+^_i_ and K^+^_int_ denote arterial plasma, venous plasma, muscle intracellular and interstitial K^+^, respectively, whilst Na^+^_a_, Na^+^_v_, Na^+^_i_, and Na^+^_int_ denote arterial, venous, muscle intracellular and interstitial Na^+^, respectively. Cl^−^_i_ and Cl^−^_int_ denote intracellular and interstitial Cl^−^, respectively. *NKA* Na^+^,K^+^-ATPase, *Nav* voltage-gated Na^+^ channel, *t-tubule* transverse tubular system, *K channels* channels permeable to K^+^, e.g. voltage gated K^+^ and K_ATP_ channels, *E*_*m*_ membrane potential, *DHPR* dihydropyridine receptors, *SR* sarcoplasmic reticulum, *RyR* Ca^2+^ release channels

The fundamental importance of K^+^ and Na^+^ for skeletal muscle activation are now well known, with knowledge of the intricate regulation of K^+^ and Na^+^ during muscle contractions and exercise developing progressively during the past century. In brief, excitation of muscle leads to membrane depolarisation caused by opening of Na^+^ channels with a concomitant Na^+^ entry. This is followed by K^+^ efflux via K^+^ channels leading to repolarisation. This sequence of events is known as the action potential (AP) which then propagates along the sarcolemma and throughout the transverse tubular network (t-tubule). These AP-induced ion movements are countered by activation of the Na^+^, K^+^-ATPase (Na^+^, K^+^-pump, NKA), resulting in an active extrusion of Na^+^ from and uptake of K^+^ into the cell, across the sarcolemmal and t-tubular membranes. The AP activates the voltage-sensing dihydropyridine receptor (Ca_V_1.1 or L-type Ca^2+^ channels) in t-tubules, which then results in the opening of sarcoplasmic reticulum (SR) ryanodine receptors (i.e., the SR Ca^2+^ channels). The subsequent Ca^2+^ release and elevation in cytosolic Ca^2+^ concentration activate cross bridge cycling and enable development of muscle force and shortening (Fig. 1). Thus, K^+^ and Na^+^ are intricately involved in membrane excitation which is a prerequisite for muscle contraction.

**Fig. 2:**
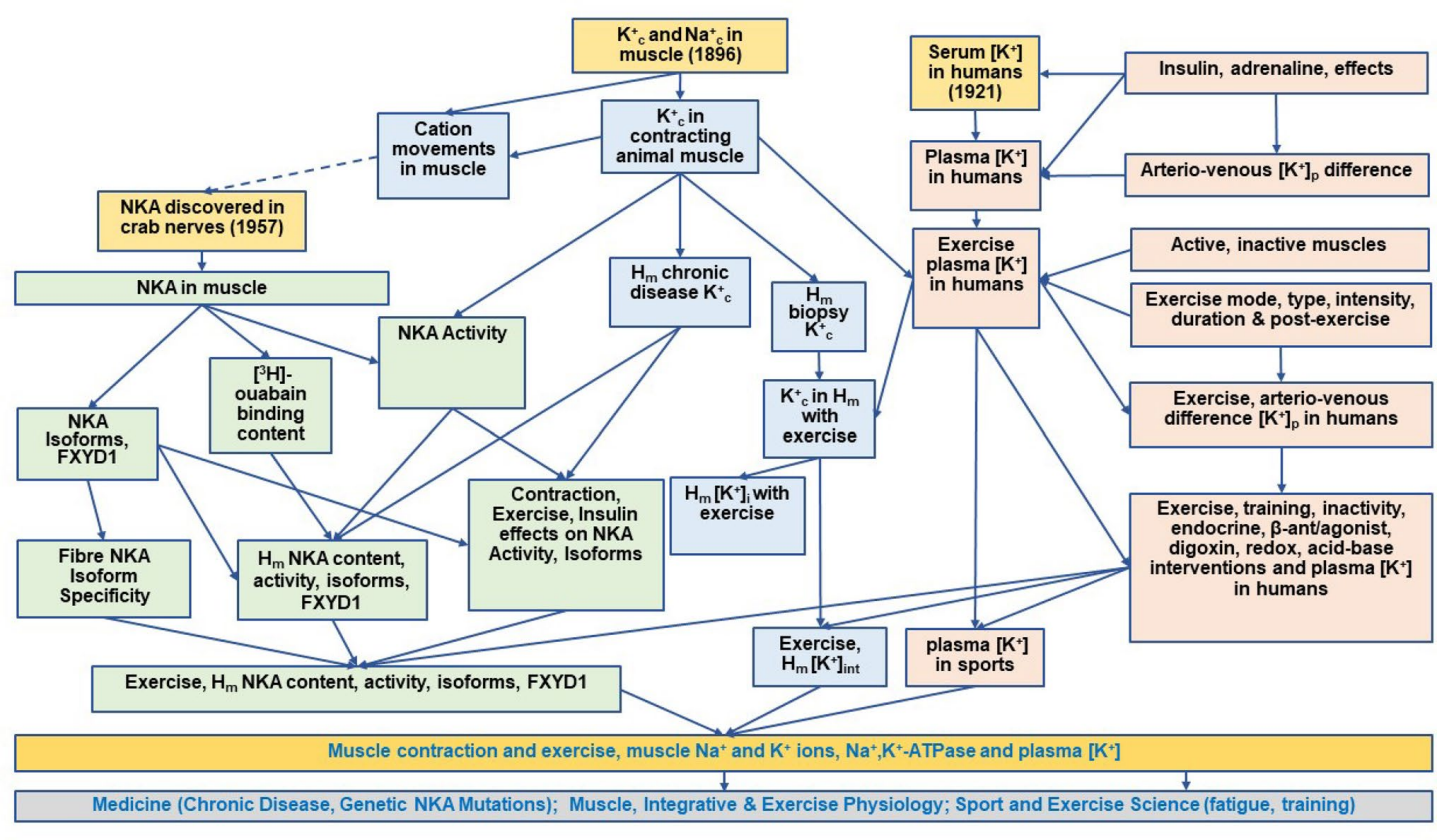
Schematic illustration of evolution of research into the effects of muscle contraction and exercise on skeletal muscle Na^+^ and K^+^ ions, Na^+^,K^+^-ATPase and on plasma K^+^ concentration. Schematic illustration of flow and connectivity of research from initial critical measurements (in light yellow boxes) of contents of K^+^ (K^+^c) and Na^+^ (Na^+^_c_) ions in skeletal muscle (m), serum K^+^ concentration ([K^+^]) in humans, and discovery of NKA; following research paths further investigating skeletal muscle ions and exercise (light blue boxes), plasma [K^+^] ([K^+^]_p_) in humans and muscle NKA activity, content and isoforms (light green boxes), all culminating in current understanding of the effects of muscle contraction and exercise on muscle Na^+^ and K^+^ ions, NKA and on plasma [K^+^]. The resulting impacts are shown (in light grey boxes) in the fields of medicine, physiology and sport and exercise science. *H*_*m*_ human muscle

This historical review outlines key chronological advances in three areas in skeletal muscle and exercise physiology that emerged and coalesced during the preceding century: (i) K^+^ and Na^+^ contents and concentrations in the intracellular and interstitial spaces in resting and contracting muscle; (ii) NKA activity, content, and isoform expression in muscle; and (iii) plasma K^+^ concentrations during and after exercise. This review starts with the initial measurements of K^+^ and Na^+^ contents in muscle, followed by changes with induced contractions and exercise, leading to the discovery of NKA and measurement in muscle, ion changes in human muscle and finishes with measurement of K^+^ in plasma with exercise and the interventions applied, as shown schematically in Fig. 2. This research culminated in understanding the effects of muscle contraction and exercise on muscle Na^+^ and K^+^, NKA and on plasma K^+^ concentration, now with applications in medicine via chronic disease, genetic NKA mutations, in muscle, integrative and exercise physiology and in sport and exercise science. This review will not discuss the physiological significance of the changes in Na^+^, K^+^ and NKA activity in regard to sarcolemmal excitability (defined as a reduction in AP amplitude or a complete loss in the capacity of the sarcolemma to generate an AP compared to AP measured in unfatigued and normal physiological conditions), on force potentiation and depression as well as the mechanisms of fatigue, as this is extensively reviewed in our companion review (Renaud et al. 2023). Furthermore, details on in-vivo and in-vitro regulation of muscle NKA, K^+^ and plasma [K^+^] with exercise are detailed elsewhere (Hostrup et al. 2021; Lindinger and Cairns 2021; Pirkmajer and Chibalin 2016).

## Early work on muscle K^+^ and Na^+^ and their movements, leading to the Na^+^, K^+^-pump discovery

Considerable research from the late nineteenth through the first half of the twentieth century measured K^+^ and Na^+^ in skeletal muscle at rest and after contractions, eventually leading to measurements of ion fluxes into and out of muscle cells.

### Early studies determining K^+^ and Na^+^ contents in resting muscle in various species

A large number of studies are detailed in Table 1, with their findings briefly summarised. The first K^+^ and Na^+^ contents’ (K^+^_c_ and Na^+^_c_, respectively) measures in skeletal muscle were in “ashed” muscle from 12 species, including humans, with values ranging from ~ 65 to 119 and from ~ 20 to 68 mmol·kg^−1^, for K^+^_c_ and Na^+^_c_, respectively (Katz 1896). During the 1910s–1940s, studies reported K^+^_c_ of ~ 80 to 110 mmol·kg^−1^ in animal muscles and from 44 to 100 mmol·kg^−1^ in human muscles, with Na^+^_c_ from ~ 6 to 38 mmol·kg^−1^ in animal muscles and from 28 to 143 mmol^.^kg^−1^ in human muscles. In over 1000 frog sartorius muscles, variations in K^+^_c_ were considerable between frogs, but small between paired muscles, with a mean K^+^_c_ of 83 mmol^.^kg^−1^ (Fenn and Cobb 1934). During the 1930s, there was considerable interest in determining whether abnormal K^+^ homeostasis in heart and skeletal muscle was an important underlying factor in chronic disease. K^+^_c_ and Na^+^_c_ were measured in hearts from persons deceased due to heart failure, pulmonary disease, or traumatic injuries (Wilkins and Cullen 1933; Calhoun et al. 1930a; Harrison et al. 1930). Cardiac K^+^_c_ from patients with heart failure was abnormally low, with the authors suggesting that K^+^ loss is one of the predisposing factors to cardiac fatigue and failure (Calhoun et al. 1930a). Several studies reported lower K^+^_c_ in *m. gastrocnemius* of patients suffering from cardiac failure than in non-cardiac patients, with values ranging from 39 to 44 mmol^.^kg^−1^ (Harrison et al. 1930; Pilcher et al. 1930), whilst this was normal (83 mmol^.^kg^−1^) in patients that had died from a variety of diseases (Cullen et al. 1933). From 1949 to 1957, muscle Na^+^_c_, K^+^_c_ and Cl^−^ contents (Cl^–^_c_) were measured in human muscle extracted during surgery or autopsy, with K^+^_c_ and Na^+^_c_ generally comparable to more contemporary measures in resting muscles (Overgaard et al. 2002). Several studies began to calculate intracellular ion concentrations in human muscles, by calculating muscle extracellular volume from the Cl^−^ space or inulin distribution, to determine the intracellular volume from the total muscle volume, which then allowed determination of intracellular ions after subtracting the extracellular ion contents. In various muscles obtained under general, spinal or local anaesthesia from healthy individuals and patients, intracellular K^+^ concentration ([K^+^]_i_) was typically around 150–160 mM, whilst the intracellular Na^+^ concentration ([Na^+^]_i_) was around 8–15 mM (Mudge and Vislocky 1949; Mokotoff et al. 1952; Horvath et al. 1955). A large study involving 46 healthy participants (13 women, 33 men) reported benchmark values for [K^+^]_i_ of 167 ± 11.9 mM (*n* = 35) and [Na^+^]_i_ of 4.4 ± 3.3 mM (n = 46) (mean ± SD^1^) (Bergström 1962). This heralded the use of needle biopsies under local anaesthesia to study human muscle at rest and after exercise, transforming exercise physiology for the next half-century. In summary, these studies over 7 decades from the late 1900s yielded variable results at first, that converged over time to form consistent findings of Na^+^_c_ and K^+^_c_ in muscle in humans and other species and also reported that [K^+^]_i_ was substantially higher than [Na^+^]_i_.

**Table 1:** Early historical findings (1896–1962) on contents of K^+^ (K^+^_c_), Na^+^ (Na^+^_c_) and Cl^−^ (Cl^–^_c_) in resting skeletal muscle in humans and in other species

References	Species	*n*	Muscle(s)	K^+^_c_ (mmol·kg ww^−1^)	Na^+^_c_ (mmol·kg ww^−1^)	Cl^–^_c_ (mmol·kg ww^−1^)
Katz (1896)	Human	2, after suicide	nr^a^	81.9	34.8	19.8
	Pig	2	nr^a^	64.9	67.8	13.7
	Beef	nr	nr^a^	93.7	28.4	16.0
	Deer	1	nr^a^	85.9	30.6	11.4
	Rabbit	2 adults	Thigh, back^a^	101.8	19.9	14.4
	Dog	1 young	Thigh, back^a^	83.2	41.0	22.7
	Cat	2 adults	Thigh, back^a^	97.9	31.7	16.0
	Chicken	1	Chest, thigh^a^	118.9	41.4	17.0
	Frog	50	“Upper tendon musculature”^a^	78.8	24.0	11.4
Meigs and Ryan (1912)	Frog	2	nr	89.5	24.0	
Mitchell and Wilson (1921)	Frog	19	*m. gastrocnemius, m. sartorius, m. vastus*	87.0		
Boutiron (1928)	Dog	nr	*m. grand oblique, m. biceps brachii, m. diaphragm*	37.3, 50.9, 41.4	37.9, 30.1, 33.4	15.5, 1.4, 11.8
	Rabbit	nr	*m. biceps brachii, m. diaphragm, m. grand oblique*	49.6, 47.1, 48.3	20.0, 14.8, 6.5	6.2, 17.1, 2.5
Norn (1929)	Human	1 (F), deceased after severe placental bleed	nr	89.3	27.8	
	Pig	1	nr	102.6	20.0	
	Rabbit	3	*m. psoas, upper extremity extensors and flexors*	108.2	19.6	
	Horse	1	nr	95.4	23.9	
	Goat	1	nr	93.6	26.1	
	Dog	3	Neck, upper and lower extremity, gluteal, back	90.5	28.3	
Ernst and Scheffer (1928)	Frog	10	*m. gastrocnemius*	87.0		
Lematte et al. (1928)	Human	nr	*m. psoas*	96.7	143.1	
	Beef		nr	138.2	34.9	
Ernst and Csúcs (1930)	Frog	7	*m. gastrocnemius*	82.1	90.9	43.7
Cullen et al. (1933)	Human	19 deceased patients (4 F/15 M)	*m. gastrocnemius*	82		40
Harrison et al. (1930)	Human	4 deceased heart failure, 2 non-heart failure patients	*m. gastrocnemius* *m. gastrocnemius*	49.1 89.0		
Pilcher et al. (1930)	Human	5 patients with cardiac disease	*m. gastrocnemius*	44.2		
Fenn and Cobb (1934)	Frog	134		83.1		
Fenn et al. (1934)	Frog	10			25.4	10.9
Fenn and Cobb (1935)	Frog	6–8	*m. sartorius, m. semitendinosis, m. tibialis anticus longus*	82.0, 79.0, 76.2		8.2
Fenn (1936)	Frog			83.0	25.4	10.9
Hastings and Eichelberger (1937)	Dog	20	*m. rectus femoris*	82.1 mmol·kg fat free^−1^	32.4 mmol·kg fat free^−1^	21.5 mmol·kg fat free^−1^
Fenn et al. (1938)	Cat	46 (K^+^),11 (Na^+^), 17 (Cl^−^)	*m. tibialis, m. EDL, m. gastrocnemius*	113.5	21.4	13.5
Mudge and Vislocky (1949)	Human	Three “normal” patients	*m. rectus abdominalis*	31.7 mmol·kg fat free^−1^	39.7 mmol·kg fat free^−1^	28.4 mmol·kg fat free^−1^
Eliel et al. (1951)	Human	6 “normal patients”	pectoral	100.4 mmol·kg dry fat free^−1^	18.1 mmol·kg dry fat free^−1^	
Iseri et al. (1952)	Human	16 “control” patients dying from non-cardiac causes	*m. pectoralis major*	94.2	40.6	29.7
Talso et al. (1953)	Human	16 patients with various non-cardiac disease	*m. rectus abdominus (13), m. latissimus dorsi (2) and m. quadratus femoris (1)*	94	33.7	19.1
Horvath et al. (1955)	Human	4 controls	*m. quadriceps*	103	32.5	
Williams et al. (1957)	Human	5 “normal” patients (no evidence of any muscular disorder)	*m. deltoid, m. gastrocnemius*	108	32.5	
Bergström (1962)	Human	46 healthy participants (13 women, 33 men, 19–59 years),	*m. quadriceps femoris*	110.9 mmol·kg fat free muscle^−1^	26.0 mmol·kg fat free muscle^−1^	19.4 mmol·kg fat free muscle^−1^

Blank cell not measured

*nr* not reported, *ww* wet weight, *F* female, *M* male

^a^20–50 g muscle used in each analysis. The age and sex of animals or humans used in these studies were not reported

### Early studies demonstrating muscle contraction effects on muscle K^+^ and Na^+^ contents

There was considerable interest during the first half of the twentieth century in Na^+^ and K^+^ movements in resting and contracting muscle. This included understanding the membrane permeability to Na^+^ and K^+^, whether this permeability and whether ion movements were active or passive. Pioneering experiments to examine ion movements in muscle, investigating the effects of NaCl, KCl and other salts on frog muscle excitability, introduced some of the key concepts of ion regulation including that: (i) extracellular NaCl was essential for excitability, (ii) addition of extracellular KCl at trace levels had a beneficial effect on muscle contractions, whereas (iii) larger KCl addition caused paralysis, (iv) Na^+^ penetrates muscle fibres and K^+^ leave them with every contraction, and (v) a mechanism must exist to prevent equalisation of these cations between the muscle sarcoplasm and interstitium (Overton 1902).

A large number of studies are detailed in Table 2 and their findings are briefly summarised here. The first reported measures of changes in muscle K^+^_c_ with contractions occurred 2 decades later, with findings that K^+^ diffuses out of fibres, that as much as half of K^+^ store may be lost in about 5 h and that there is a “loss of irritability” and considerable muscle swelling when frog *m. gastrocnemius* was electrically stimulated beyond physiological limits (Mitchell and Wilson 1921). Subsequently large K^+^_c_ decreases and Na^+^_c_ increases were reported in perfused frog *m. gastrocnemius* directly stimulated until fatigue, whereas there were no changes in K^+^_c_ in muscles indirectly stimulated via the nerve (Ernst and Fricker 1934; Ernst and Scheffer 1928; Ernst and Csúcs 1930). Initially, the K^+^ losses were considered to result from K^+^ released from bound potassium within muscle, an increased membrane permeability or from muscle damage (Ernst and Csúcs 1930). However, the concept that all K^+^ was bound in muscle was then disproven (Callison 1931). One study reported that stimulation via the sciatic nerve of dog *m. gastrocnemius* for 5–8 h and 11–13 h reduced muscle K^+^_c_ by 9.2 and 22.6 mmol·kg^−1^, respectively (Calhoun et al. 1930b), whilst others found no change in K^+^_c_ in stimulated frog muscle (Mond and Netter 1930).

**Table 2:** Early historical findings (1921–1938) of electrical stimulation or exercise effects on K^+^, Na^+^ and Cl^−^ contents in skeletal muscle in different species

References	Species	*n*	Muscle(s)	Stimulation/exercise	K^+^_c_ (mmol·kg ww^−1^)		Na^+^_c_ (mmol·kg ww^−1^)		Cl^–^_c_ (mmol·kg ww^−1^)	
					Rest	Post	Rest	Post	Rest	Post
Mitchell and Wilson (1921)	Frog		*m. gastrocnemius*	Perfused K^+^-free ringer 5.3 h (no stim) Plus Stim via lumbar plexus (30 min 1 s tetani .03TPS, 30 min rest) for:	74.7	59.6				
				1.5 h	89.5	78.3				
				for 2.5 h	76.5	60.6				
				for 8.5 h	58.8	51.9				
				for 2 h then direct stim until fail to respond	68.0	36.1				
				for 6.25 h to exhaustion	56.5	26.1				
Ernst and Scheffer (1928)	Frog	10	*m. gastrocnemius*	Stim nr	87.0	77.0				
Ernst and Csúcs (1930)	Frog	7	*m. gastrocnemius*	Direct stim 0.3–0.4 s tetani to fatigue	82.0	43	90.9	146.0	43.8	27.8
Calhoun et al. (1930b)	Dog	10	*m. gastrocnemius*	Stim via sciatic n						
				Twitches: 0.5–200 Hz for 5–8 h	90.5	81.3				
				for 11–13 h	94.9	72.3				
Fenn and Cobb (1936)	Frog	8 6	nr	10–400 tetani.min^−1^, 10–30 min						
				Stim “Indirect”—via sciatic nerve	46.6^a^	45.7^a^				
				Stim “Direct”—via electrodes on knee/ankle	46.2^a^	43.6^a^				
Fenn and Cobb (1936)	Rat	14 (11 for Cl^−^)	*m. gastrocnemius*	Stim via sciatic n 1 Hz, 5–30 min	47.3^a^	41.2^a^	7.6	15.9^a^	5.4^a^	8.2^a^
Fenn (1937)	Rat	9 8 8 9	*m. gastrocnemius* *m. tibalis* *m. biceps femoris* *m. semi-membranosus*	Swim to exhaustion 15–120 min	48.0^a^ 46.4^a^ 46.7^a^ 50.7^a^	44.6^a,b^ 45.0^a,b^ 43.9^a,b^ 45.5^a,b^				
Fenn et al. (1938)	Cat	46 (K^+^),11 (Na^+^),17 (Cl^−^)	*m.gastrocnemius* *m. tibialis, m.EDL*	Stim: 25 s tetani at 1 Hz, 30–60 min	43.1^a^	38.2^a^	8.8^a^	17.2^a^	5.5^a^	9.7^a^
Tipton (1938)	Cat	15	*m. gastrocnemius*	Stim: maximal shocks, 660 Hz, 30 min	40.2^a^	33.0^a^	7.8^a^	14.8^a^	5.5^a^	7.8^a^

Ions were measured at rest and after (post) exercise or electrical stimulation (stim). The age and sex of animals in these studies were not reported; blank cell indicates that variable was not measured

*n* number of animals or muscles analysed, *nr* variable was not reported, *TPS* train per s

Ion measures were in mmol per kg wet weight (ww), unless indicated as ^a^mmol per 100 g dry weight; ^b^the paired denervated muscles were used as resting muscles as they could not be activated during swimming

Major progress then occurred during the 1930’s from Fenn and colleagues (Fenn and Cobb 1934, 1936; Fenn 1936, 1937, 1938, 1939; Fenn et al. 1934, 1938). Collectively, these studies demonstrated: (i) frog *m. sartorius* incubated for up to 7 h lost more K^+^ and had a more rapid loss of “irritability” (i.e., excitability) when exposed to high CO_2_; (ii) frog muscle directly stimulated via electrodes showed only a small loss of K^+^_c_ (6.1 mmol.kg^−1^) in severe fatigue, whilst force declined by 66–75%, but with no loss in K^+^_c_ when muscle was indirectly stimulated via the sciatic nerve; (iii) contrary to frog muscle, rat muscles stimulated via the sciatic nerve lost K^+^ (6.1 mmol·kg^−1^), along with gains of Na^+^ (8.3 mmol.kg^−1^), Cl^−^ (2.8 mmol.kg^−1^) and water (15–25%), which were all reversible during recovery; (iv) muscle lost K^+^_c_ and gained water after “voluntary” swimming in rats, with the greatest muscle K^+^ loss seen in animals that swam the longest; (v) in stimulated cat muscle, K^+^ losses increased with greater contraction intensity and stimulation duration from 5 to 35 min; (vi) of the K^+^ liberated from stimulated cat muscle, 31% was absorbed by the liver, little was taken up by resting muscles, with only a small increase in plasma [K^+^]. In 1940, Fenn summarised key perspectives about the physiological importance of K^+^: (i) “…the cells are permeable to K^+^ but not to Na^+^”; (ii) “the activity of muscle is always accompanied by a loss of K^+^”; (iii) “the loss of K^+^ is in general proportional to the duration and the intensity of the contraction”; (iv) “possibly the progressive loss of K^+^ is one of the factors which causes the intensity of contraction to decrease” and finally, (v) “in small concentrations potassium is excitatory and in larger concentrations it is inhibitory” (Fenn 1940). These dual physiological roles of K^+^, excitatory (now known as potentiating) and depressive (possibly as part of fatigue) are extensively discussed in our companion review (Renaud et al. 2023).

### Early studies demonstrating K^+^ and Na^+^ fluxes in muscle at rest and after contractions

Two major questions investigated during the 1940s and 1950s where whether the membrane permeability to Na^+^ and K^+^ were altered by contractions and whether Na^+^ and K^+^ movements were active or passive. In 1941, experiments demonstrated that resting frog *m. sartorius* accumulated K^+^ against a concentration gradient, whilst the membrane was impermeable to Na^+^ (Boyle and Conway 1941), although the latter conclusions on Na^+^ impermeability were then criticised (Krogh 1946). Concurrently, it was shown that ^42^K uptake in *m. gastrocnemius* of swimming rats was fourfold greater than in resting rats and it was concluded that there was a bi-directional movement of K^+^ into and out of muscle during work (Hahn and Hevesy 1941). In the same year, Dean proposed “*there must be some sort of pump, possibly located in the fibre membrane, which can pump out the sodium or, what is equivalent, pump in the potassium*” (Dean 1941). The reciprocal nature in muscle that K^+^ leaves the cells and Na^+^ enters, also with the reverse exchange were clearly noted under a variety of conditions, including muscle contractions (Steinbach 1947). He also confirmed in vertebrates that muscle K^+^_c_ was 10–33 times greater than in plasma, whereas muscle Na^+^_c_ was 0.13–0.30 that of plasma (Steinbach 1947).

All the above studies suggested that membrane permeability to various ions differs between resting and active muscles, which was eventually confirmed. Two studies demonstrated that at rest, the cell membrane of frog skeletal muscle was permeable to K^+^ and Cl^−^ but almost impermeable to Na^+^ (Hodgkin and Horowicz 1959; Hutter and Noble 1960), which was later confirmed for mammalian muscles (Bryant and Morales-Aguilera 1971). Another study reported that during an AP in giant squid axon, Na^+^ permeability increases via the activation of voltage-sensitive Na^+^ channels, allowing Na^+^ influx during the depolarization phase, whilst K^+^ permeability increases during the repolarization phase allowing K^+^ efflux (Hodgkin and Huxley 1952). Other studies then confirmed the same for AP generation in skeletal muscle (Nastuk and Hodgkin 1950), being a vital step in the activation of contraction.

In summary, studies up to around 1950 demonstrated that whilst, at rest, the muscle cell membrane is primarily permeable to K^+^ and Cl^−^, it becomes permeable to Na^+^ and K^+^ when it generates APs. During muscle activity where multiple APs are generated, the Na^+^ influx during depolarization results in an increased [Na^+^]_i_, whilst the K^+^ efflux during repolarization results in an increased [K^+^]_e_. Thus, the central mechanisms responsible for the Na^+^ influx and K^+^ efflux during muscle activity were understood. The next issue was to understand the reverse flux, i.e., Na^+^ efflux and K^+^ influx.

The coincident inward and outward fluxes of radioactive Na^+^ and K^+^ in muscle provided early evidence that led to discovery of an active Na^+^/K^+^ transport system. Incubation of frog *m. sartorius* in low K^+^ solutions followed by recovery resulted in an outward Na^+^ extrusion and inward K^+^ movement, although the K^+^ uptake was considered at that time to be passive (Steinbach 1951, 1952). After ^24^Na^+^ loading, ^24^Na^+^ efflux at 18 °C from *m. sartorius* had “rapid” (1–3 h) and slow fractions, with total ^24^Na^+^ flux greatly reduced at 0 °C (Harris and Burn 1949; Harris 1950) and with similar findings in rat diaphragm muscle at 38 °C (Creese 1954). Inward and outward ^42^K^+^ movements were found in frog muscles, with both ^42^K influx and efflux increased with elevated external [K^+^] ([K^+^]_e_), concomitantly with greater ^24^Na (active) efflux, with the latter reduced when the muscles were bathed in K^+^-free solution (Creese 1954; Carey and Conway 1954; Keynes 1954). It was concluded that in amphibian muscles, “there may be a definite linkage between the inward movement of potassium and the outward movement of sodium” (Keynes 1954). Findings that the cardiac glycosides, strophanthidin and digitoxin, caused inhibition of active K^+^ and Na^+^ transport in red blood cells (Schatzmann 1953) and that ouabain inhibited the net transport of Na^+^ out of and K^+^ into frog *m. sartorius* (Johnson 1956) were key for the next major advance in understanding mechanisms of Na^+^ and K^+^ movements in muscle, i.e., the discovery of the NKA.

### Identification of NKA by Jens Skou, the Nobel Prize and the Post-Albers pump cycle

An ATPase enzyme activity was first investigated in crab isolated leg nerves and found to be dependent upon Na^+^, K^+^, Mg^2+^, Ca^2+^ and H^+^ concentrations, deduced as a Na^+^-Mg^2+^-ATP dependent process, that was also activated by K^+^ and possibly involved in the active extrusion of Na^+^ from the nerve fibre (Skou 1957). Inhibitory effects of g-strophanthin on this Na^+^-K^+^ activated ATPase activity were later demonstrated (Skou 1960) and with detailed evidence later described for the enzymatic, ATP-dependent active transport of Na^+^ and K^+^ across the cell membrane, its location in cellular membranes and inhibition by cardiac glycosides (Skou 1965). Skou received the Nobel Prize for Chemistry in 1997 “for the first discovery of an ion-transporting enzyme, Na^+^, K^+^-ATPase” (Skou 1998; Clausen and Persson 1998).

**Fig. 3:**
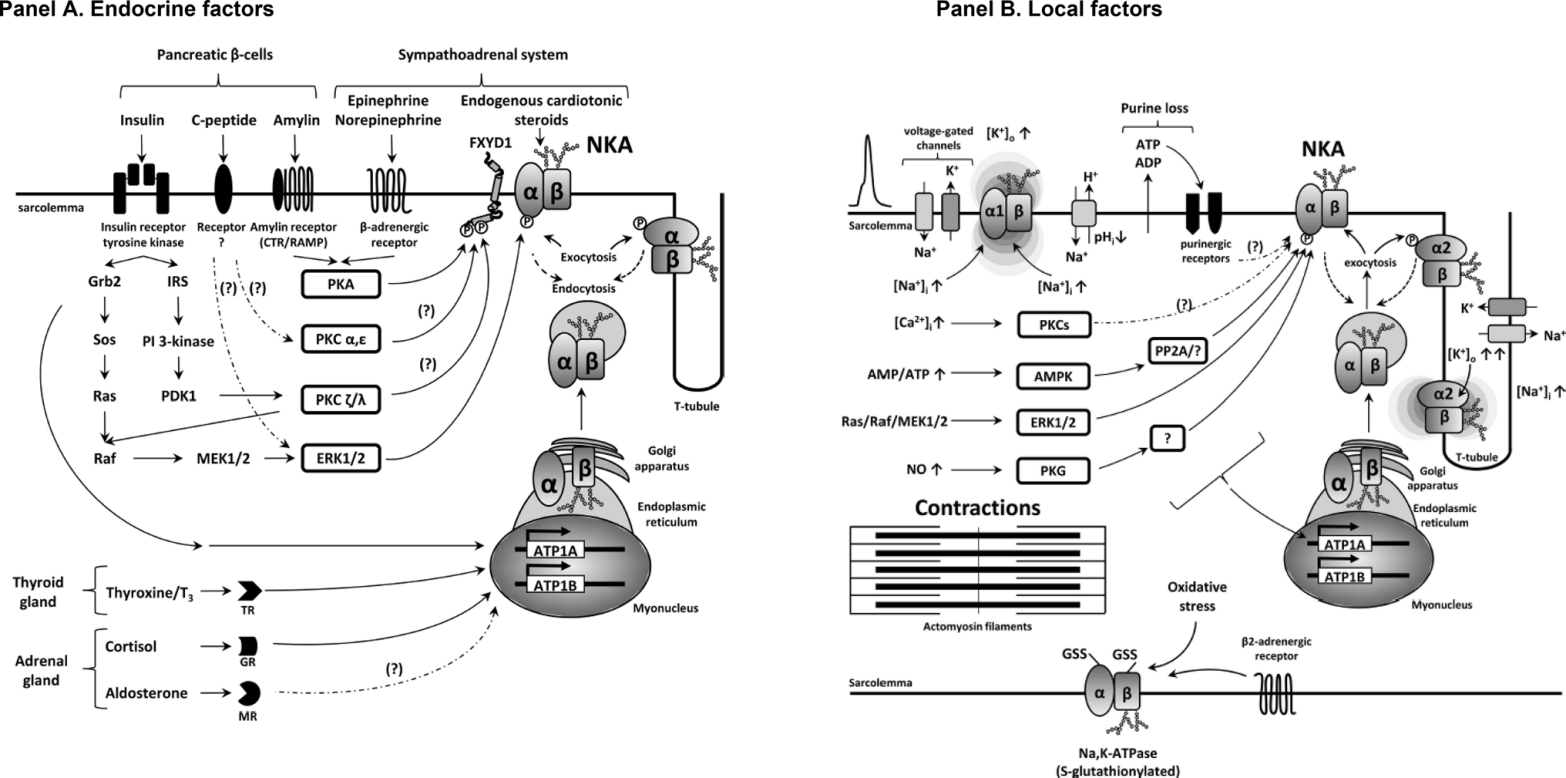
Receptors and pathways involved in regulation of NKA in skeletal muscle involving **A** endocrine factors, including insulin and catecholamines and **B** local factors. From Pirkmajer and Chibalin (2016) with permission. Detailed descriptions of regulatory factors, their receptors, pathways and actions are given in Pirkmajer and Chibalin (2016). *AMP* adenosine monophosphate, *ATP* adenosine triphosphate, *AMPK* AMP kinase, *cAMP* cyclic AMP, *Ras* Raf, *MEK1/2* kinase upstream of ERK1/2, *PKC* protein kinase C, *PKG* PP2a, *NO* nitric oxide, *GSS* glutathione, *FXYD1* phospholemman, *IRS* insulin receptor substrate, *PI3-kinase*, phosphoinositide 3-kinase, *PDK1* phosphoinositide-dependent protein kinase 1, *TR* thyroid hormone receptor, *GR* glucocorticoid receptor, *MR* mineralocorticoid receptor, *ATP1A* gene for NKA α_1_-subunit, *ATP1B* gene for NKA β_1_-subunit

This finding led to a global, ongoing explosion of research into NKA. The NKA is ubiquitously expressed and is embedded in plasma membranes, which in skeletal muscle comprise the sarcolemma and t-tubules (“Muscle NKA isoforms, FXYD, localisation, effects of exercise, genetic manipulations and their functional significance”, Fig. 1). The NKA functions primarily as a cellular transmembrane cation active transporter, respectively, extruding 3 Na^+^ and accumulating 2K^+^ ions against their electrochemical gradients per cycle (Post et al. 1960, 1967; Post 1989) and also exerting a small electrogenic effect on the cell membrane potential (Clausen 1986). This involves phosphorylation by ATP, the binding and release of Na^+^ and K^+^, known as the Post-Albers model of the pump cycle, with steps blocked by specific NKA-inhibitors, such as ouabain, digoxin, and other ouabain-like compounds (Fedosova et al. 2021). The NKA also functions as an intracellular signal transducing protein, involved in a number of signalling pathways (Xie and Askari 2002), and is a cellular receptor for endogenous ouabain and ouabain-like compounds (Schoner 2002; Blaustein et al. 2022). Many key historical developments related to NKA typically occurred in tissues other than in skeletal muscle and are therefore not considered in this paper, including the first determination of the NKA crystal structure (Morth et al. 2007), subsequent studies on structure and differences between the NKA isoforms, and identification of impacts of mutations in NKA structure on pump function and on their role in various diseases (Clausen et al. 2017; Morth et al. 2009; Heinzen et al. 2014; Biondo et al. 2021; Friedrich et al. 2016). The acute and/or chronic regulation of NKA in muscle is extensive, including a highly complex interplay of neural, humoral, ionic, redox, metabolic and genetic factors, with these and its implications for K^+^ and Na^+^ homeostasis described elsewhere (Clausen 1986, 2003, 2010; Clausen and Everts 1989; Pirkmajer and Chibalin 2016; Ewart and Klip 1995; McDonough and Youn 2005; Geering 2006; Hostrup et al. 2021; Lindinger and Cairns 2021). A schematic summarising the complex endocrine and regulatory factors involved in NKA regulation in muscle, and their receptors and pathways is shown in Fig. 3.

**Fig. 4:**
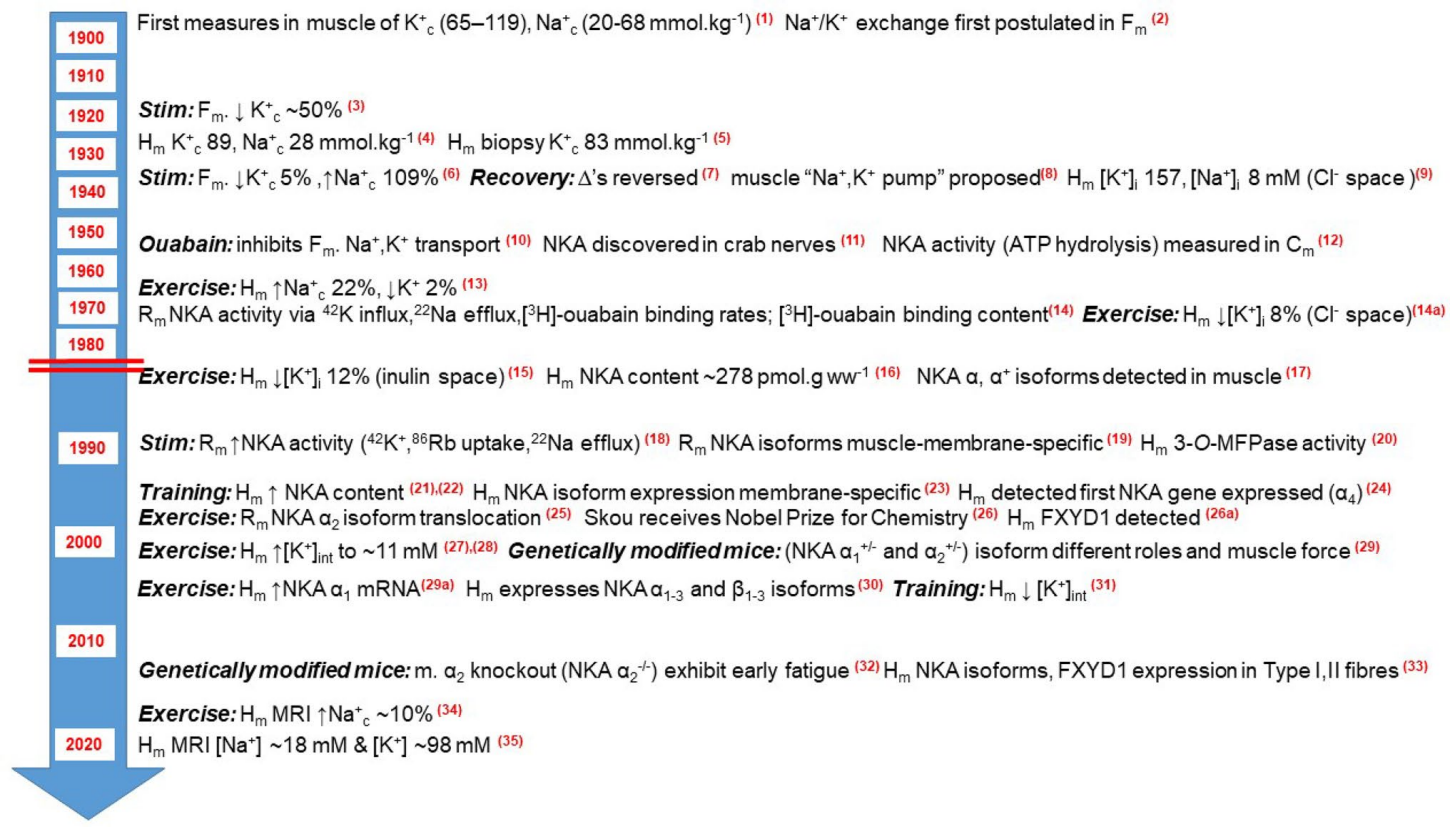
Timeline of selected key findings on Na^+^ and K^+^ ions, and of NKA in skeletal muscle at rest and with exercise, with focus on findings in human muscle. All findings are from measures in muscle obtained from humans (H_m_), rats (Rat_m)_, frogs (Frog_m_) or mice (Mouse_m_), except for discovery of NKA in crab nerves. Measures refer to resting muscle unless specified as following stimulation (Stim.) or Exercise. Interventions or use of mouse genetic modification models are indicated by bold, italicised text. Red horizontal lines indicate different time-scale after the split. All NKA disease-related discoveries are omitted from this figure. *Na*^*+*^ sodium ion, *K*^*+*^ potassium ion, *Na*^*+*^_*c*_ sodium ion content, *K*^*+*^_*c*_ potassium ion content, *[ion]* ion concentration, *i* intracellular, *int* interstitial, *ECW* extracellular water determined by (method), *NKA* Na^+^, K^+^-ATPase; NKA α^(+/−)^ or ^(−/−)^, modified mouse isoform lacking one or both copies of the gene encoding for that α isoform; 3-*O*-MFPase, 3-*O*-methyl fluorescein phosphatase; FYXD1, phospholemman; MRI, magnetic resonance imaging. References: (1) (Katz 1896); (2) (Overton 1902); (3) (Mitchell and Wilson 1921); (4) (Norn 1929); (5) (Cullen et al. 1933); (6) Fenn and Cobb 1936); (7) (Fenn et al. 1938); (8) (Dean 1941); (9) (Mudge and Vislocky 1949); (10) (Johnson 1956); (11) (Skou 1957); (12) (Bonting et al. 1961); (13) (Bergstrom and Hultman 1966); (14) (Clausen and Hansen 1974); (14a) (Sahlin et al. 1977); (15) (Sjogaard and Saltin 1983); (16) (Norgaard et al. 1984); (17) (Lytton et al. 1985); (18) (Everts et al. 1988); (19) (Hundal et al. 1992); (20) (Benders et al. 1992); (21) (Green et al. 1993); (22) (McKenna et al. 1993); (23) (Hundal et al. 1994); (24) (Shamraj and Lingrel 1994); (25) (Tsakiridis et al. 1996); (26) (Clausen and Pearson 1998); (26a) (Garvey et al. 1998); (27) (Green et al. 2000), (28) (Juel et al. 2000); (29) (He et al. 2001); (29a) Nordsborg et al. 2003a); (30) (Murphy et al. 2004); (31) (Nielsen et al. 2004); (32) (Radzykevich et al. 2013); (33) (Thomassen et al. 2013); (34) (Hammon et al. 2015); (35) (Gast et al. 2022b)

The focus of following sections is primarily on the effects of muscle contractions or exercise on NKA in skeletal muscle, with inclusion of related effects induced by elevated insulin and catecholamines. This first addresses the quantitative measurements of NKA activity and content in muscle, both critical to understanding NKA regulation, adaptability and function, especially during and after exercise. This is followed by the discovery of NKA subunits, isoforms, accessory proteins and more recently, by the genetic manipulation of NKA isoforms to examine their functional significances. The section concludes with a focus on human muscle, including the muscle cation changes with exercise in humans and of NKA. The important physiological roles of NKA in attenuating the K^+^-induced force depression and optimising muscle contraction at the onset of muscle activity are detailed in our companion review (Renaud et al. 2023). A timeline of key developments in the measurements of Na^+^ and K^+^ in skeletal muscle at rest and with exercise, the discovery of NKA and the effects of exercise on muscle NKA activity, content, and isoforms are shown in Fig. 4.

## NKA activity in skeletal muscle and the effects of muscle contractions and exercise

Major developments over the past 7 decades of NKA research included quantification of NKA activity in muscle in animals and humans and investigated the effects of a plethora of physiological perturbations with implications for K^+^ and Na^+^ homeostasis. This section briefly outlines key developments and applications in the measurement of NKA activity in muscle, culminating with measures of NKA activity in human muscle samples at rest and with exercise. Important issues addressed include how NKA activity was measured, in what type of preparation and the limitations of these approaches, with details on how NKA activity is regulated indicated by reference to other reviews.

### Activity determined by ATP hydrolysis rates

Mg^2+^-activated ATPase activity had been observed in rat hindlimb muscle in 1948 (Kielley and Meyerhof 1948a, 1948b) and shortly after Skou’s discovery of NKA, the first measurements of NKA activity in muscle appeared (Bonting et al. 1961). They determined NKA activity as the ouabain-inhibitable component of total ATPase activity in homogenates of various tissues from cats, including skeletal muscle and stated: “The enzyme has been variously called membrane ATPase, pump ATPase, ouabain-sensitive ATPase, strophanthidin-sensitive ATPase, magnesium-sodium-activated ATPase, and sodium-stimulated ATPase. It would seem more appropriate to label this enzyme sodium–potassium-activated ATPase (Na–K ATPase)…” A key subsequent finding was that the ouabain sensitivity of NKA activity differed between tissues (Bonting et al. 1962), although the existence of NKA isoforms to account for differing ouabain sensitivities would not be apparent for some decades (“Muscle NKA isoforms, FXYD, localisation, effects of exercise, genetic manipulations and their functional significance”). A strong temperature dependence of NKA activity was then found in frog *m. EDL* homogenates, with activity reduced from values at 37 °C by 89% at 0.5 °C and a general significance of NKA for repolarisation in excitable tissue was suggested (Bonting and Caravaggio 1963). Purification of NKA enriched preparations then showed that NKA was highly associated with plasma membranes and paved the way for detailed biochemical investigations into the regulation of NKA activity (Jørgensen 1974). Using skeletal muscle preparations, the separation of purified muscle plasma membrane fragments or membrane vesicles by ultracentrifugation then enabled NKA activity measurement in enriched samples (Narahara et al. 1979; Seiler and Fleischer 1982). The major advantage of this approach was the high NKA activity found. Disadvantages, however, included the large amount of tissue (200 g) and long time (2 days) required, but critically also the extremely low yield of only 0.01–0.02 mg protein^.^g^−1^, raising the risk that these membrane preparations may not be representative of the full population of NKA in the tissue (Seiler and Fleischer 1982; Mickelson and Louis 1985). The yield of NKA when using isolated and purified membranes was mostly around only a few percent (0.2–8.9%) of total NKA (Clausen 1986; Hansen and Clausen 1988). To avoid the issue of low yield, measurement of NKA activity in crude homogenates was suggested, but at that time was rarely undertaken (Hansen and Clausen 1988). Another problem with the methodology was that these in-vitro measures of NKA activity are undertaken at optimal conditions for the enzyme reaction, which reflects the enzyme maximal rate and NKA content or maximal NKA activity, rather than the in-vivo NKA activity of the muscle. Further methodological development was needed to assess activity in-vivo and the effects of acute activation of muscle.

### Activity determined by labelled K^+^, Rb^+^ and Na^+^ ion fluxes and by rate of ouabain binding

#### Activity in intact muscles and muscle pieces

An important approach to studying NKA activity was the use of radio-labelled ion fluxes which could be employed in intact muscle or muscle pieces. From maximal NKA activity of 67 μmol g^−1^ h^−1^ in muscle, a Na^+^ efflux of 10.9 pmol^.^(cm^2^)^−1^ s^−1^ and K^+^ influx of 8.8 pmol^.^(cm^2^)^−1^ s^−1^ were calculated, suggesting a large NKA-driven transport capacity for Na^+^ and K^+^ (Bonting and Caravaggio 1963). This was confirmed in rat isolated *m. soleus* where NKA activity was determined using ^42^K influx and ^22^Na efflux rates and which for the first time in muscle also investigated [^3^H]-ouabain binding (Clausen and Hansen 1974). Important findings included that: (i) [^3^H]-ouabain bound to the external surface of the plasma membrane of muscle; (ii) ouabain markedly reduced muscle ^22^Na efflux; (iii) at rest, each ouabain-binding site actively transported around 500 Na^+^ and 325 K^+^ ions per minute, or ~ 2.4% of calculated maximal activity (Clausen and Hansen 1974). It was later concluded that in resting muscle, NKA activity represented only ~ 5 to 6% of the basal metabolic rate (Chinet et al. 1977) and at 30–35 °C utilised only 2–6% of the total capacity for active Na^+^/K^+^ transport (Clausen 1986). This was consistent with later calculations that NKA activity in muscle consumes only 5–10% of the total ATP turnover in working fibres (Clausen et al. 1991; Ørtenblad et al. 2009).The calculation of percent total NKA capacity was established by comparing the resting ^42^K or ^86^Rb uptake (a marker for K^+^ uptake) with the maximal capacity for ^86^Rb^+^ uptake, which established that a huge reserve capacity exists for increasing Na^+^/K^+^ transport in muscle (Clausen et al. 1987). Validating the methodology, in non-contracting, isolated rat *m. soleus*, the ouabain-suppressible ^22^Na efflux was ~ 1.5 times greater than the ouabain-suppressible ^42^K influx and thus compatible with the expected 3:2 Na^+^/K^+^ exchange (Clausen and Kohn 1977). Furthermore, a strong linear relationship was found between the ouabain-suppressible ^86^Rb^+^ uptake rate and the number of available functional NKA units in muscle (Kjeldsen et al. 1985b).

The next critical investigations explored the maximal capacity of NKA in muscle, determining NKA activity in intact *m. soleus* from rats under conditions designed to induce maximal activity of the pumps, measuring each of ^42^K^+^ and ^86^Rb^+^ uptake rates, ^22^Na^+^ efflux rates, the net changes in Na^+^_c_ and K^+^_c_ (Clausen et al. 1987). Key findings included that: (i) full activation of all NKA required very high [Na^+^]_i_, which was achieved through Na^+^-loading to a non-physiological [Na^+^]_i_ of ~ 125 mM and [K^+^]_e_ of 100–130 mM; (ii) in these Na^+^-loaded muscles, the ouabain-suppressible net Na^+^ loss and K^+^ gain were 6000 and 5300 nmol g^−1.^min^−1^, respectively, whilst the corresponding ouabain-suppressible ^22^Na^+^ efflux and ^86^Rb^+^ uptake peak rates were 6500 and 5800 nmol g^−1^ min^−1^, respectively; (iii) a 1:1 relationship existed between ^42^K^+^ and ^86^Rb^+^ uptake rates, indicating that ^86^Rb^+^ uptake could adequately reflect K^+^ influx and (iv) the maximum ouabain-suppressible rates of active Na^+^–K^+^ transport corresponded to levels predicted by their [^3^H]-ouabain-binding site content. Hence, all NKA in muscle were shown to be functional and almost complete utilisation of all available NKA could be achieved with ensuing very high rates of active Na^+^/K^+^ transport.

#### NKA activity in muscle transverse tubule membranes

Radiolabeled ion tracers were also used to quantify NKA activity in vesicles from purified membranes from muscle to enable study of Na^+^/K^+^ transport in isolated specific membranes. This included the critical determination of the Na^+^/K^+^ exchange capacity in the t-tubules, using isolated vesicles comprising membranes from the t-tubular system from rabbit *m. sacrospinalis* (Lau et al. 1979). By measuring rates of ^22^Na and ^86^Rb transport, they demonstrated active Na^+^/K^+^ exchange in the t-tubules that was regulated by NKA.

### K^+^-dependent phosphatase activity

During the 1960s–1980s, the K^+^-dependent phosphatase activity that is a component of the NKA cycle was utilised to enable sensitive biochemical measures of NKA activity in small tissue samples, without relying on the more complex measures of radiolabeled ion transport. These biochemical investigations into NKA properties investigated the reactions that comprise the NKA cycle. These assays used either *p*-nitrophenyl phosphate (*p*-NPP) or the fluorogenic compound 3-*O*-methyl fluorescein phosphate (3-*O*-MFP) as substrates to determine phosphatase activity as a marker of NKA activity, with maximal rates measured in-vitro, under optimised conditions. As the use of 3-*O*-MFP led to later controversies regarding NKA activity in muscle, including in humans (“K^+^-dependent phosphatase activity, Summary of NKA activity measurements in resting muscle during the 1960s–1980s”), some details of early development of the assays are included.

The presence of a phosphatase that split *p*-NPP, was stimulated by K^+^ and inhibited by ouabain was demonstrated in purified membranes (Judah et al. 1962a, b), that was part of the ATPase reaction (Ahmed and Judah 1964) and was a possible final step in NKA, since the K^+^-activated *p*-nitrophenyl phosphatase (*p*-NPPase) activity and NKA activity shared numerous broadly similar characteristics, including K^+^-activation and inhibition by ouabain (Albers and Koval 1966). The K^+^-dependent phosphatase activity was confirmed to be a partial reaction of NKA (Askari and Koyal 1968; Uesugi et al. 1971). It was later concluded that the K^+^-dependent phosphatase activity associated with NKA, thought to represent the terminal step in ATP hydrolysis, is a sensitive measure of NKA activity suitable for use in small tissue samples (Hansen and Clausen 1988).

The subsequent use of 3-*O*-MFP enabled measures of NKA activity in even smaller tissue samples, because this assay was highly sensitive, requiring only 1–2% the amount of tissue needed for other NKA activity assays and was specific for NKA, because it was inhibitable by ouabain (Huang and Askari 1975). The K^+^-dependent 3-*O*-MFPase method was employed in skeletal muscle and concluded to be a reliable means of determining numbers of NKA in muscle (Nørgaard et al. 1984b). Exhibiting NKA-specificity via ouabain–inhibition and thus being unaffected by the large abundance of other ATPases in muscle, as well as being suitable for muscle biopsies, the K^+^-dependent 3-*O*-MFPase assay was subsequently used to study contraction and exercise effects on NKA activity in animal and human muscles (“Summary of NKA activity measurements in resting muscle during the 1960s–1980s, Increased muscle NKA activity with muscle contractions).

### Summary of NKA activity measurements in resting muscle during the 1960s–1980s

In summary, studies quantified NKA activity in resting muscle using intact isolated muscles, isolated membrane fractions and homogenates, utilising techniques to measure ATP hydrolysis rates by inorganic phosphate production, transport rates of ^42^K^+^, ^86^Rb^+^ and/or ^22^Na^+^, *p*-NPPase activity and 3-*O*-MFPase activity, with each preparation and technique having distinct advantages and disadvantages (Table 3). In general, muscle exhibits low NKA activity under resting conditions but has a large reserve capacity. However, further methodological development is required as none of the mentioned methods allow for direct measurements of NKA activity in exercising humans in vivo.

**Table 3:** Advantages and disadvantages of typical methods used to specifically detect NKA activity and proteins in skeletal muscle preparations

NKA activity method	Advantages	Disadvantages
ATP hydrolysis rate	Enables measures in-vitro Ouabain-inhibitable indicating specific measure of NKA activity (total—ouabain-inhibitable ATPase activity) Detects activity utilising full NKA cycle Can be used to indicate maximal NKA activity High sensitivity measure of NKA activity if linked with measures of radiolabeled P	Does not indicate activity in-vivo Dual measures (total—ouabain-inhibitable ATPase activity) increase measurement variability High ouabain concentrations are needed to inhibit the α_1_ isoform in rat and mouse muscle
	Normally used in muscle homogenates, which allows recovery of all NKA molecules in muscle	Homogenate measures includes small risk of contamination by non-muscle tissue including blood, interstitial fluid, nerve, adipose tissue In animals and humans, NKA activity is low compared with myosin ATPase and Ca^2+^-ATPase activities increasing risk of measurement error from smaller percentage ATPase
	Can be used in isolated membrane preparations (e.g., sarcolemma, transverse tubules, vesicles), enabling detection of highest NKA activity	Isolated membrane preparation measures have very low yield, require relatively large tissue mass, extensive preparation time and may be unrepresentative of NKA population in tissue studied. Includes moderate-high risk of contamination by membranes from other sources
Radiolabelled K^+^, Rb^+^ and Na^+^ fluxes	Enables measures in-vitro and in-situ Ouabain-inhibitable, NKA specific activity Detects activity utilising full NKA cycle can be used to indicate maximal NKA activity High sensitivity measure of NKA activity can be used with intact muscles and isolated membrane vesicle preparations	Does not indicate activity in-vivo Dual measures (total—ouabain-inhibitable fluxes) increase measurement variability Requires use of radioactive compounds Cannot be used in biopsies without prior preparation of membrane vesicles
Rate of [^3^H]-ouabain binding	Enables measures in-vitro and in-situ Ouabain-inhibitable, NKA specific activity Detects activity of functional NKA but stopped at ouabain binding	Does not indicate activity in-vivo Requires use of radioactive compounds
*p-*NPPase activity	Enables measures in-vitro Ouabain-inhibitable, NKA specific activity with ouabain inhibition allows specific measurement of NKA activity Specific for NKA ouabain inhibitable, K^+^-stimulated Suitable for small muscle pieces from animals and human muscle biopsy samples	Does not indicate activity in-vivo Detects only phosphatase activity which is terminal step in NKA activity cycle, not full ATPase cycle
3-*O*-MFPase activity	Enables measures *in-vitro* Ouabain-inhibitable, NKA specific activity Suitable for small muscle pieces from animals and human muscle biopsy samples	Does not indicate activity in-vivo Detects only phosphatase activity which is terminal step in NKA activity cycle, not full ATPase cycle Is not Na^+^-dependent

NKA protein measures	Advantages	Disadvantages
[^3^H]-ouabain-binding site content	Enables measures in-vitro In animal models and humans fully quantifies content in molar units of the most abundant α_2_ isoform (~ 80%) Suitable for small muscle pieces from animals and human muscle biopsy sample	In many animals, standard assay does not detect low affinity α_1_ and α_3_ isoforms (~ 20%) and detection of α isoforms thus varies with the ouabain sensitivity of tissue and thus the ouabain concentration used Requires use of radioactive compounds
	In human muscle assay fully detects all α isoforms (100%), which have similar ouabain affinities, hence measures NKA content	In humans cannot be used in-vivo due to ubiquitous expression and relatively high prevalence of NKA in all tissues
Western blotting using specific antibodies against NKA isoforms	Enables measures in-vitro Determines relative abundance of NKA isoforms (relative to other tissues, rest samples, a control sample etc.) Requires only very small tissue sample High sensitivity measure of NKA isoforms Suitable for small muscle pieces from animals and human muscle biopsy samples	Non-quantitative as not quantified in molar terms and is expressed relative to other tissues, rest samples etc. Does not measure content of NKA isoforms Non cross-reactivity of antibodies against other isoforms needs to be established
Detection through imaging in tissue, using specific antibodies against NKA isoforms	Enables measures in-vitro Allows isoform detection in transverse slices, longitudinal sections of muscle Is quantitative when coupled with immuno-gold labelling and detection using EM Can be applied to very small samples	Non cross-reactivity of antibodies against other isoforms needs to be established Most studies demonstrate presence at site only, but which is not quantified

Methodological factors and specific technical limitations of assays and assay conditions are described in original articles cited in appropriate sections in the review

*p-*NPPase, *p-*nitro phenyl phosphatase, *3-O-MFPase* 3-*O*-methyl fluorescein phosphatase, *EM* electron microscopy

### Increased muscle NKA activity with muscle contractions

Critical developments during the 1980s and 1990s extended these measurements of NKA in resting intact muscle by demonstrating that muscle NKA activity in rat muscles was rapidly and markedly increased during and following electrically activated contractions. These findings were fundamental to understanding Na^+^ and K^+^ regulation in muscle and in blood during exercise and recovery (Sects. “Na^+^ and K^+^ ion concentrations in human skeletal muscle with exercise–Specific intervention effects on plasma [K^+^] with exercise, linked with perturbations in muscle NKA activity).

#### Immediate effects of contraction on NKA activity measured in intact muscles

Excitation via electrical stimulation elicited large activation of NKA in rat isolated, intact *m. soleus* and *m. EDL* that were directly stimulated at 0.5–20 Hz for 10 s–15 min, with NKA activity determined immediately after, by ouabain-suppressible ^42^ K^+^ or ^86^Rb^+^ uptake and ^22^Na^+^ efflux rates (Everts et al. 1988). In *m. soleus*, stimulation for 15 min at 2 Hz and 5 Hz increased ouabain-suppressible ^86^Rb^+^ uptake by 110% and 67% above resting values, respectively, whilst after 20 Hz stimulation for 10 and 60 s, the increases were by 65% and 86%, respectively. In support, the ouabain-suppressible ^22^Na^+^ efflux was increased after 1 Hz and 2 Hz stimulation by 54% and 68%, respectively. In *m. EDL*, the resting ouabain-suppressible ^86^Rb^+^ uptake was 17% larger than in *m. soleus* and was also increased after 2 Hz stimulation, but only by ~ 31%, much less than in *m. soleus*. All of these changes occurred without changes in the average muscle intracellular Na^+^ or K^+^ contents, leading to the proposal that a Na^+^-independent mechanism of NKA activation was involved (Everts et al. 1988). As responses to stimulation and elevated adrenaline were not additive, it was concluded these likely involved common initial steps in activation pathway. The greater activation in *m. soleus* than *m.* EDL was also suggested to account for the greater fatigue resistance of slow than fast muscles. At this time, stimulation via the nerve of *m. soleus* in anaesthetised rats for 4 s at 20 Hz every 5 s for 5 min was shown to induce muscle hyperpolarization after the contractions, that could be abolished by ouabain, cooling or removal of K^+^ and thus supporting an excitation-activated, electrogenic NKA in muscle during the recovery period (Hicks and McComas 1989). Greater activation of NKA with stimulation was confirmed in rat *m. soleus* compared to *m. EDL*, being due to greater sensitivity to intracellular Na^+^_c_ and with NKA rapidly and dramatically activated by up to 15-fold by excitation (Everts and Clausen 1994). These effects were further examined in rat isolated *m. soleus*, either contracting isometrically, or allowed to shorten without force when stimulated, with measures of Na^+^ fluxes, intracellular Na^+^_c_ and ^22^Na^+^ efflux (Nielsen and Clausen 1997). After stimulation for 30 s at 60 Hz, the intracellular Na^+^_c_ was initially increased, but then fell in recovery to undershoot 32% below control, sustained for 30 min. The net Na^+^ extrusion was blocked by ouabain, indicating that it was due to NKA activity, with rates of ^22^Na^+^ efflux dependent on stimulation duration and frequency. After high-frequency stimulation, NKA activity was increased 22-fold in the first 30–50 s after contraction and reached the maximum theoretical transport capacity. Thus, during high-frequency stimulation of rat muscles, a dramatic increase in NKA activity was found, that occurred with increased intracellular Na^+^_c_ but also independently of Na^+^_c_, evidenced by increased activity even without gain in intracellular Na^+^_c_ and sustained during the intracellular Na^+^_c_ undershoot in recovery, with NKA phosphorylation proposed as a possible stimulatory mechanism (Nielsen and Clausen 1997). These mechanisms were proposed to protect the muscle from run-down of Na^+^ and K^+^ gradients and thus also against fatiguability during contractions (Nielsen and Clausen 1997).

Collectively, these studies demonstrated that in isolated, intact slow twitch and fast twitch muscles in rats, NKA was rapidly and substantially activated in an activation-frequency dependent manner by contractions even as short as 1–10 s, with elevated activity sustained for a considerable period post-contraction. Elevated NKA activity has important implications for muscle function: (i) in the maintenance of membrane excitability during contractions, by counteracting the Na^+^ influx and K^+^ efflux associated with AP’s, which preserves Na^+^ and K^+^ gradients and directly by contributing electrogenically to resting *E*_m_ (Renaud et al. 2023); (ii) in the post-exercise restoration of excitability and (iii) in minimising the exercise hyperkalaemia and enabling its subsequent rapid recovery post-exercise, which also accounts for the hypokalaemia that can occur for several minutes after high intensity exercise (Sect. Specific intervention effects on plasma [K^+^] with exercise, linked with perturbations in muscle NKA activity).

#### Effects of muscle contraction on NKA activity, measured in muscle membrane fractions and homogenates

The above measures were in isolated intact muscles and represent acute regulation of NKA activity under near-physiological conditions. However, it was of interest also to determine whether increased maximal NKA activity (or NKA capacity) would occur in muscle membrane preparations or homogenates following contractile activation and whether this was related to translocation of functional NKA units within the cells. One early report measured NKA activity (ATP hydrolysis) in a sarcolemmal preparation, 24 h after contraction, finding that 15 min stimulation increased maximal NKA activity by up to 28% above rest (Brodal et al. 1975). However, later studies that quantified the effects of exercise or electrical stimulation on 3-*O*-MFPase activity, in whole muscle homogenates or in muscle membrane fractions, showed contrasting results. Thus, exercise (running) or electrical stimulation was suggested to inactivate NKA as measured by reduced maximal 3-*O*-MFPase activity in rat muscle (Fowles et al. 2002; Mishima et al. 2008), whereas no change in 3-*O*-MFPase activity was found in *m. EDL* after stimulation (Goodman et al. 2009). In contrast, however, electrical stimulation of rat *m. soleus* increased 3-*O*-MFPase activity in muscle homogenates by 40–53% and in sarcolemmal fractions by 37–40%, along with increased NKA α subunits and of [^3^H]-ouabain-binding site content in homogenate (Sandiford et al. 2005). This suggested that increased NKA activity occurred as a result of both increased NKA α subunit availability and translocation to the plasma membrane. Subsequently, several studies supported the notion of improved maximal NKA activity in various membrane fractions through translocation of NKA isoforms. The central evidence for this was that acute exercise and electrical stimulation of rat muscles increased NKA activity, measured by both 3-*O*-MFPase activity and by Na^+^-stimulated hydrolysis of ^32^P-ATP, as well as by an increased abundance of NKA α subunits in sarcolemmal giant vesicles and in an enriched outer membrane fraction containing both sarcolemmal and t-tubular membranes (Kristensen et al. 2008; Rasmussen et al. 2008; Juel 2009). In contrast, measures in whole muscle homogenates showed no increase of maximal NKA activity. Thus, studies in purified muscle membrane fractions of sarcolemmal origin collectively indicated that measures of NKA activity were increased by electrical stimulation and by exercise and that this may be consequent in part to translocation of NKA to these surface membranes, or by structural alterations in caveolae.

In summary, measurements in animal muscles over 4 decades indicate that large and rapid increases in muscle NKA activity occur during and immediately after electrical stimulation and exercise, caused both by increased intracellular [Na^+^] and by increased Na^+^-affinity of NKA, which under extreme conditions can approach maximal theoretical activity. Measures of NKA activity in-vitro in purified sarcolemmal membrane fractions further suggest that increased NKA activity after contractions may also result from translocation of NKA subunits to plasma membranes.

### NKA activity in human muscle at rest and with exercise

#### Resting muscle

The first measurements of NKA activity in human muscle utilised the maximal K^+^- stimulated 3-*O*-MFPase assay in crude homogenates prepared from muscle biopsies (Benders et al. 1992) which was later modified to enable reliable, ouabain-inhibitable maximal NKA activity measurements in *m. vastus lateralis* biopsies (Fraser and McKenna 1998). More recently, an alternate NADH-linked method was developed for human muscle samples, which was fully inhibited by 2 mM ouabain and yielded maximal NKA activity corresponding to theoretical maximal values predicted from reported ouabain-binding content (Jannas-Vela et al. 2019).

#### Exercise and recovery

The maximal K^+^-stimulated 3-*O*-MFPase activity assay has been widely utilised in human exercise studies, with the first finding that fatiguing, repeated knee extensor contractions reduced the maximal 3-*O*-MFPase activity by 14% in *m. vastus lateralis* biopsies (Fraser et al. 2002). This observation was corroborated by findings from nine acute exercise studies in humans from two laboratories, showing ~ 11 to 35% reductions in 3-*O*-MFPase activity after a range of fatiguing exercise types and durations (McKenna et al. 2008) and also being reversible by 3 h post-exercise (Sostaric et al. 2022). The phenomenon was referred to as exercise-induced inactivation of NKA, as the NKA content did not decline, and was suggested to reflect inhibitory actions of reactive oxygen species, or increased cytosolic [Ca^2+^] in muscle fibres on NKA activity and was proposed to be an important mechanism leading to muscle membrane depolarisation and contributing to muscle fatigue (McKenna et al. 2008). A further possible explanation for reduced maximal 3-*O*-MFPase activity with exercise is an increased glutathionylation of NKA in muscle (Juel et al. 2015). However, the validity and significance of these activity findings was challenged, positing that the maximal K^+^-stimulated 3-*O*-MFPase activity measure was “an inappropriate method for ATPase quantification” (Juel 2012), whilst others also pointed out limitations with the method (Broch-Lips et al. 2012). Key criticisms included that the assay is Na^+^-independent and thus cannot measure activity under physiological conditions of elevated [Na^+^] and that these in-vitro measurements do not reflect in-vivo NKA activity (Juel 2009, 2012; Broch-Lips et al. 2012). The significance of these 3-*O*-MFPase activity measures in human *m. vastus lateralis* was further challenged by findings that maximal NKA activity measured by the rate of Na^+^-dependent ^33^P-ATP hydrolysis was increased by 19% after 4 min intense exercise, whereas the K^+^-stimulated 3-*O*-MFPase activity declined after exercise, and was also insensitive to a stable ADP analogue and to protein kinase C activation, both of which increase NKA activity (Juel et al. 2013). They concluded that the 3-*O*-MFPase activity method is not suited to detect changes in NKA activity in muscle with exercise. However, in contrast, a subsequent study from the same laboratory found that NKA activity in human *m. vastus lateralis* measured via rates of Na^+^-dependent ^33^P-ATP hydrolysis was actually reduced after intense, fatiguing exercise (Hostrup et al. 2014b). Thus, in that study, reduced maximal NKA activity directly measured by ATP hydrolysis rates after exercise was consistent with the previous findings of reductions in maximal 3-*O*-MFPase activity (Juel et al. 2013).

#### Conclusions on measurement of NKA activity in human muscle and functional implications

In human muscle, NKA activity has been mostly assessed as maximal K^+^-stimulated 3-*O*-MFPase activity. Controversy exists, however, regarding the effects of acute exercise on NKA activity in human muscle, with the *in-vitro* maximal K^+^-stimulated 3-*O*-MFPase method typically showing a reduction in activity post-exercise, which is not always consistent with the activity determined by the rate of Na^+^-dependent ^33^P-ATP hydrolysis. Further studies with exercise in humans are required to clarify these discrepancies. However, comparisons should not be drawn between measures of maximal rates of NKA activity in muscle determined in vitro under optimised laboratory conditions and the actual NKA activity occurring in vivo. Indeed, measures of plasma [K^+^] changes in femoral venous plasma during and after intense leg exercise suggest that in-vivo activation of NKA probably only reaches 15–25% of the maximal theoretical activity (Hallén et al. 1994). Accordingly, none of the *in-vitro* maximal measures reflect actual in-vivo NKA activity. This would require either development of techniques to accurately and directly measure NKA activity in-vivo, or alternate functional measures influenced by NKA activity, such as Na^+^ and K^+^ ion movements in muscle cells, muscle interstitial fluid or in blood plasma or red cells. Studies using indwelling K^+^-selective electrodes in humans conclude that an initial lag in NKA activity, as well as only fractional activation occur in muscle with exercise (Sect. Specific intervention effects on plasma [K^+^] with exercise, linked with perturbations in muscle NKA activity). An interesting possibility is that an initial lag followed by submaximal activation of NKA in muscle with exercise allows muscle interstitial [K^+^] to increase, which can then potentiate muscle twitch and submaximal contractions and thus facilitate ongoing muscle performance. In contrast, an eventual decline in maximal NKA activity by whichever mechanism is responsible may then allow greater increases in interstitial [K^+^], that could then have inhibiting effects on muscle function, i.e., fatigue. This dual role of elevated [K^+^] in muscle is discussed in our companion review (Renaud et al. 2023). More intensive focus on muscle NKA activity and exercise is required in humans, including comparison of multiple methodologies to resolve current controversies such as the proposed inactivation of maximal NKA activity with exercise.

## NKA content in skeletal muscle, including the effects of insulin, exercise, training and aging

This section outlines some of the key early developments in measures of [^3^H]-ouabain-binding site content in animal muscles, and examines this as a measure of NKA content in human muscle and its implications. The studies behind the proposal that insulin, electrical stimulation and exercise can each increase [^3^H]-ouabain binding in muscle due to translocation of NKA subunits to plasma membranes are covered. Furthermore, the effects of physical training, inactivity as well as age on [^3^H]-ouabain-binding site content are also covered.

### [^3^H]-ouabain-binding site content in animal muscles

A key advance in muscle NKA research was the development of the [^3^H]ouabain-binding site content method to quantify NKA molecules (Clausen and Hansen 1974). This method is now recognised as a gold-standard approach to quantify NKA in muscle (Clausen 2008, 2013) and has enabled extensive analyses over the past half-century of the intricate and interactive effects of a huge array of hormonal, dietary, environmental, behavioural and other factors regulating NKA content, as well as enhancing understanding in many clinical conditions, exercise and sport science applications (Clausen 1986, 1996, 2003, 2013; Hansen and Clausen 1988; Clausen and Everts 1989; Clausen et al. 1998; Clausen 2008).

The [^3^H]-ouabain binding to muscle was measured to quantify NKA, based on the strong affinity of ouabain for NKA and binding in a 1:1 proportion and was found not to differ between intact muscles and cut muscle pieces (Clausen and Hansen 1974). Whilst the *early rate* of [^3^H]-ouabain binding to rat *m. soleus* during incubation was increased by insulin and adrenaline, the final steady-state [^3^H]-ouabain binding was not increased (Clausen and Hansen 1977). Vanadate (VO_4_), which is structurally similar to phosphate (PO_4_), was found to facilitate binding of ouabain and was therefore introduced in the [^3^H]-ouabain-binding site measures (Hansen 1979). The [^3^H]-ouabain-binding site method was found to validly measure NKA in cut muscle pieces from rat *m. soleus* and *m. EDL* (Nørgaard et al. 1983), which then paved the way for measurement of NKA content in muscle biopsies obtained from humans (Sect. NKA content in human muscle).

The [^3^H]-ouabain-binding site content in muscle varies considerably across species (Clausen 1986) and in animals, changes substantially with age (Sect. Aging) and differs between muscles with different fibre types. In young rats (4 week), muscle [^3^H]-ouabain-binding site content is typically higher in fast twitch muscles with higher glycolytic potential such as *m. EDL*, than in slow twitch muscles such as the more oxidative *m. soleus*. Thus, in young rats, the *in-vitro* [^3^H]-ouabain-binding site content was 21–27% higher in *m. EDL* than *m. soleus* (Clausen et al. 1982, 2004; McKenna et al. 2003). In adult rats, this relationship with oxidative potential was, however, suggested to be reversed (Chin and Green 1993).

An important question is whether the [^3^H]-ouabain-binding site content method detects all NKA in the muscle preparation. In rat muscle, the NKA α_1_ isoform has a low affinity for ouabain (i.e. is ouabain insensitive) and thus is not detected in the standard ouabain-binding site assay, which indicates that this assay measures the *content* of the α_2_ isoform only (also α_3_, although this is probably very low) (Hansen 2001). The only study to have determined the molar amount of NKA α_1_ isoform in rat *m. soleus* and *m. EDL* quantified this at ~ 135 to 220 pmol g ww^−1^, around 15–25% of all NKA, meaning that in rats, the actual NKA total content would be 20–30% greater than the measured [^3^H]-ouabain-binding content (Hansen 2001). An important implication is that intervention studies in rats using muscle [^3^H]-ouabain-binding site content will measure the dominant α_2_ isoform, whilst any changes in the α_1_ isoform will not be detected.

### NKA content in human muscle

The [^3^H]-ouabain-binding site content developed to determine NKA in human muscle biopsy pieces (Nørgaard et al. 1984a) has been widely employed in healthy individuals and those with chronic disease (Clausen 1986; Murphy et al. 2007; Green et al. 1993; Evertsen et al. 1997), with the measured range in healthy human muscle typically between 243 and 425 pmol g ww^−1^ (Clausen 2013). A vital difference exists in the interpretation of the muscle [^3^H]-ouabain-binding site content when measured in humans versus in other animals. Wang and colleagues demonstrated that the affinity of ouabain binding was high for the three main NKA α isoforms, α_1_, α_2_ and α_3_ (Wang et al. 2001), each of which are expressed in human muscle (Murphy et al. 2004). Thus, the [^3^H]-ouabain-binding site content in human muscle therefore also represents the total NKA content (NKA_c_) and is now the gold-standard for full quantification of NKA_c_ in human muscle. Hence, interventions that modify NKA_c_ in human muscle indicate a change in the total pool of NKA in that tissue, although they do not differentiate which of the three α isoforms are changed.

Numerous human clinical studies have measured muscle NKA_c_ in a diversity of diseases, often revealing substantial up- or down-regulation of NKA_c_ in muscle, including patients with hyper- and hypothyroidism, diabetes, McArdles disease, heart failure and myotonic dystrophy (Clausen 1998), chronic obstructive lung disease (Ravn and Dorup 1997), alcoholism (Aagaard et al. 2003), spinal cord injury (Ditor et al. 2004; Boon et al. 2012), as well as heart, lung or kidney transplant recipients (Williams and McKenna 2012). These findings demonstrate the enormous clinical implications of muscle NKA research. Measurements of muscle NKA_c_ in humans are predominantly in biopsies from *m. vastus lateralis* muscle, with few studies comparing NKA_c_ in biopsies from other muscles. The limited data available from these studies did not show systematic variation between NKAc of human muscles (Nørgaard et al. 1984a; Dorup et al. 1988a; Nordsborg et al. 2005a), except for cases in which muscles were subject to severe inactivity due to paraplegia (Ditor et al. 2004) or shoulder impingement (Leivseth and Reikeras 1994).

### Insulin, contraction and exercise effects on muscle [^3^H]-ouabain-binding site content

A key question, as previously mentioned in the section “Effects of muscle contraction on NKA activity, measured in muscle membrane fractions and homogenates”, is whether increased muscle NKA activity with contractions/exercise might reflect an increased muscle [^3^H]-ouabain-binding site content, due to translocation of NKA molecules from intracellular or sub-sarcolemmal sites to the plasma membranes.

#### Insulin

The concept of translocation of NKA can be traced to early studies that found increased [^3^H]-ouabain binding in frog muscles exposed to insulin, which was suggested to be due to an “unmasking” of latent NKA sites in muscle (Grinstein and Erlij 1974; Erlij and Grinstein 1976). However, in these experiments, incubation in [^3^H]-ouabain was only for 50 min (Erlij and Grinstein 1976), which was insufficient time to achieve saturation of [^3^H]-ouabain binding to muscle (~ 2 h) and thus for full quantification of all NKA (Clausen 2003). Several studies then used longer incubation periods to achieve a plateau in [^3^H]-ouabain-binding and failed to detect an increase in [^3^H]-ouabain-binding with insulin in mouse and rat muscle, despite increased NKA activity evidenced by increased ^86^Rb uptake (Clausen and Hansen 1977; Dorup and Clausen 1995; McKenna et al. 2003). However, the [^3^H]-ouabain-binding site technique was also recently criticised as being unable to detect trafficking of NKA molecules to the plasma membrane, due to the slow binding kinetics of ouabain (Pirkmajer and Chibalin 2016). Nonetheless, the lack of an increase [^3^H]-ouabain binding in most studies does not support an insulin-stimulated increase in [^3^H]-ouabain-binding site content in muscle.

#### Electrical stimulation

Electrical stimulation increases NKA activity and thereby also the early rate of [^3^H]-ouabain binding to rat *m. soleus* (Everts and Clausen 1994). To determine whether increased NKA activity was accompanied by an increased appearance of NKA in muscle surface membranes, which might reflect NKA translocation from intracellular sites, the effects of electrical stimulation were investigated in isolated rat *m. soleus* and *m. EDL* (McKenna et al. 2003). High intensity stimulation increased NKA activity substantially, but did not increase the [^3^H]-ouabain-binding site content in *m. soleus* or *m. EDL* (McKenna et al. 2003), which argues against a role for NKA translocation in the increased NKA activity with muscle activation.

#### Acute exercise in humans

Several experiments also investigated whether acute exercise in humans increased NKA_c_ in *m. vastus lateralis*, with conflicting findings. After a 100 km run that lasted ~ 11 h, the muscle NKA_c_ was 13% greater than at 4 weeks prior (Overgaard et al. 2002). This could result from translocation, but considering the long time course of exercise, might reflect increased synthesis of NKA, or simply variation during the pre-race period. Consistent with the above, during 16 h of 6 min exercise bouts at 91%*V*O_2peak_ repeated each hour, the NKA_c_ was not altered immediately after each bout, but was increased by ~ 5% and ~ 7% by the 9th and 16th bouts, respectively (Green et al. 2007). Furthermore, the NKA_c_ was increased by ~ 15% after 2 h cycling at 62%*V*O_2peak_ (Green et al. 2011) and recently, by 10% after 20 min submaximal cycling, which was proposed to be due to rapid formation of functional NKA molecules from existing, but not bound, α and β subunits within the muscle (Sostaric et al. 2022). In contrast, no change was found in NKA_c_ after sprint cycling (~ 52 s) at ~ 170% peak power output (Aughey et al. 2006), or after submaximal cycling to fatigue (~ 54 to 72 min) (Leppik et al. 2004; Murphy et al. 2006). The reasons for these varying findings with exercise on NKA_c_ in humans remains to be determined.

In summary, early reports of insulin-stimulated increases in [^3^H]-ouabain-binding site content in rat muscle could not be confirmed in rat or mouse muscles when sufficient time for full saturation of all NKA sites by ouabain was utilised. Furthermore, whilst electrical stimulation acutely increased muscle NKA activity in rat isolated muscles, this was not associated with an increased [^3^H]-ouabain-binding site content. Finally, studies in humans have yielded conflicting findings regarding exercise effects on muscle NKA_c_, but the reasons for this discrepancy are unresolved.

### Effects of training, inactivity and aging on muscle [^3^H]-ouabain-binding site content

Numerous studies have investigated the effects of physical training or inactivity (McKenna et al. 1996; Wyckelsma et al. 2019), chronic disease (Clausen 1998) and aging on human muscle NKA_c_ (Wyckelsma and McKenna 2016).

#### Training

Early studies typically showed that training in animals increased muscle [^3^H]-ouabain-binding site content. Thus, [^3^H]-ouabain-binding site content was increased after endurance training in muscles from rats (Kjeldsen et al. 1986), guinea pigs (Leivseth et al. 1992) and horses (McCutcheon et al. 1999) and also after sprint training in horses (Suwannachot et al. 1999)*,* although one study found no increase after training in rats (Galuska et al. 2009). Importantly, the magnitude of these increases in muscle [^3^H]-ouabain-binding site content was typically 20–40%, but was greater if training either directly followed, or was compared to inactivity (Kjeldsen et al. 1986; Leivseth et al. 1992). Similar training-induced increases were also evident in disease models, such as in rats with diabetes induced by partial pancreatectomy (Schmidt et al. 1994) and with surgically induced myocardial infarction (chronic heart failure) (Helwig et al. 2003).

In healthy humans, 12 studies from 1990 to 2017 investigated the effects of training on NKA_c_ in *m. vastus lateralis*, with consistent findings that endurance, high intensity and resistance training induced an 8–25% upregulation of NKA_c_, which was unrelated to mean training intensity, cumulative training time or training duration (Wyckelsma et al. 2019) and a similar upregulation in NKA_c_ after resistance training was recently confirmed (Altarawneh et al. 2020). In chronic heart failure patients, there was no effect of training on *m. vastus lateralis* NKA_c_ (Green et al. 2001), whilst in contrast, in young patients with Type I diabetes, NKA_c_ was increased by 8% after sprint training (Harmer et al. 2006). It was proposed that an upper limit, or plateau that occurs in human muscle NKA_c_ with training reflects a balance between beneficial functional outcomes through improved Na^+^/K^+^ handling in muscles and in plasma with exercise, against potential adverse consequences such as the risks of post-exercise hypokalaemia for myocardial arrhythmias (Wyckelsma et al. 2019). The increase in NKA_c_ after training is consistent with the typical lowering after training of the muscle interstitial [K^+^] ([K^+^]_int_) and circulating [K^+^] during exercise (Sects. “Human skeletal muscle interstitial [K^+^] with exercise, Specific intervention effects on plasma [K^+^] with exercise, linked with perturbations in muscle NKA activity”) and may reduce muscle fatigue and facilitate muscle performance (Renaud et al. 2023).

#### Inactivity

Early studies using animal models of inactivity demonstrated reductions in muscle [^3^H]-ouabain-binding site content by around 20% in rat and guinea pig muscles (Kjeldsen et al. 1986; Leivseth et al. 1992), with these changes coinciding with impairments in muscle contractile function. The effects of physical inactivity on muscle NKA_c_ in humans are not well understood, being investigated in only six studies, but with most of these utilising injury models involving a cross sectional design (Wyckelsma et al. 2019). Reductions in NKA_c_ after injury include by 20–23% after knee ligament injury, 34–45% after spinal injury and 27% with shoulder impingement syndrome (Wyckelsma et al. 2019). Only one study investigated the effects of restricted activity alone, finding no change in NKA_c_ after 23 days of unilateral lower limb suspension (Perry et al. 2016). Further research into inactivity effects on muscle NKA_c_ in humans is clearly warranted.

#### Aging

There are tremendous differences with age in [^3^H]-ouabain-binding site content in animal muscles (Wyckelsma and McKenna 2016), increasing from birth to peak values in immature animals, then declining through young and adult animals and with further modest decline in older adults (Kjeldsen et al. 1984, 1985a; Clausen et al. 1982), with substantial differences also in NKA isoform expression (Orlowski and Lingrel 1988). The potential impact of aging on human muscle NKA is therefore of interest. However, the *m. vastus lateralis* NKA_c_ determined after autopsy in 18 children from one day to 8 years of age did not differ from adult muscle (Kjeldsen and Gron 1989). Little is known about the effects of aging on NKA_c_ in human adults, with the few studies restricted to cross sectional study designs and often with a small sample size. However, in the age ranges studied, no apparent decline in muscle NKA_c_ occurred. Thus, when data from 57 healthy participants were compared, there was no difference in *m. vastus lateralis* NKA_c_ between subgroups of adults aged between 18 and 76 years (Wyckelsma and McKenna 2016) and others also found no apparent differences in adults across different ages (Klitgaard and Clausen 1989; Dorup et al. 1988a, b). Thus, the large decline seen with aging after early peak in immature animals is not evident in human muscle. One possibility is that prolonged reduced activity in rats due to their long-term housing in cages is responsible for these divergent responses in muscle NKAc content between rats and humans. However, studies are required in humans beyond 80 years of age. Nonetheless, the marked decline in muscle mass with aging means that despite unchanged NKA_c_, the overall NKA-mediated capacity for K^+^ regulation is substantially reduced with age.

## Muscle NKA isoforms, FXYD, localisation, effects of exercise, genetic manipulations and their functional significance

### Overview of NKA isoforms and FXYD1 in muscle

The NKA belongs to a multi-gene family and exists as a heterodimer comprising a catalytic α subunit with 4 isoforms (α_1_–α_4_) and a heavily glycosylated, regulatory β subunit with three isoforms (β_1_–β_3_), together with a regulatory accessory protein, FXYD, with seven isoforms (FXYD_1_–FXYD_7_) (Fedosova et al. 2021; Blanco and Mercer 1998; Garty and Karlish 2006; Yap et al. 2021; Geering 2006). The importance of these different isoforms and accessory proteins in muscle is demonstrated through their differing intracellular locations, abundances, fibre-type specific expression and physiological roles. In brief, the most abundant NKA α isoforms in muscle, α_1_ and α_2_, are primarily involved in regulating Na^+^/K^+^ exchange and contributing to *E*_m_, but with α_1_ involved under rest and α_2_ under exercise conditions, whilst α_1_ is also involved in intracellular signalling pathways mediated by cardiotonic steroids. Thus, conditions or interventions that change the overall or site-specific abundance of these isoforms are likely to modulate those local regulatory effects. FXYD1 is expressed in muscle and changes in the overall or site-specific abundance, or phosphorylation status of FXYD1 will also modulate NKA activity.

### NKA isoform and FXYD expression in animal skeletal muscle

#### Discovery of NKA isoforms in muscle

After the discovery of NKA in 1957, it took several decades to realise that this comprises a family of proteins with multiple subunits, isoforms and an accessory protein, with NKA isoforms encoded by separate genes, and with differing sensitivity to ouabain and K^+^ affinities (Jørgensen 1974; Sweadner 1989; Lingrel et al. 1990). Two biochemically distinct molecular forms of NKA, then referred to as α and α^+^ (Sweadner 1979), later identified, respectively, as being α_1_ and likely both α_2_ and α_3_ (Sweadner 1989) were first detected in muscles in rats (Lytton et al. 1985). In rats, *m. soleus* had predominantly high-affinity ouabain binding sites (Kjeldsen et al. 1985b), indicative of the α_2_ isoform, which was detected as the predominant isoform in rat hindlimb muscles, also with expression of α_1_ and α_3_ (Urayama et al. 1989). Further, α_1_ in rats was resistant to (i.e., marked insensitivity, low affinity to) ouabain, being 100-fold more resistant to ouabain than α_2_ and α_3_, both of which had a high affinity to ouabain (Lingrel 1992; Blanco and Mercer 1998).

#### NKA isoform cellular locations and insulin-induced translocation

Two fundamental questions regarding NKA isoforms in animal muscles addressed from around 1980 were: (i) where are NKA molecules and specifically, the different isoforms located? and (ii) can physiological stimuli (e.g., insulin) induce translocation of NKA isoforms within the muscle? In frog muscle treated with glycerol to cause detubulation, ~ 80% of NKA ([^3^H]-ouabain binding) were in the surface membrane and ~ 20% in t-tubular membranes, but given the much larger surface area of the t-tubule membranes, the NKA density was 4–5% of that in surface membranes (Venosa and Horowicz 1981). Detection of NKA in the t-tubules is consistent with other studies in amphibian and mammalian muscle (Lau et al. 1979; Ariyasu et al. 1987; Donoso and Hidalgo 2002). A series of studies during the 1990s then made important advances in demonstrating membrane-specific NKA isoform expression, with most finding higher α_1_, α_2_ and β_1_ abundances in plasma membrane fractions than internal membrane fractions (Hundal et al. 1992, 1993, 1994; Marette et al. 1993; Lavoie et al. 1996, 1997). Using crude membrane preparations from rat mixed hindlimb muscles (after an overnight fast), each of the α_1_, α_2_, β_1_ and β_2_ isoforms were expressed, with higher α_1_, α_2_ and β_1_ in purified plasma membrane fractions, compared to purified internal membranes (10%, 17% and 20% compared to plasma membrane, respectively) and with higher β_2_ abundance in internal membranes (Hundal et al. 1992). However, contrary to their studies above, they also reported that both a_2_ and β_1_ were several-fold more abundant in internal than in plasma membrane fractions in red and white hindlimb muscles in rats (after an overnight fast) (Lavoie et al. 1996). Immunogold labelling and electron microscopy then revealed α_2_ in the plasma membrane, in intracellular tubular and vesicular structures in sub-sarcolemmal and triadic regions, as well as in the perinuclear area in rat *m. soleus*, *m. gastrocnemius* and *m. quadriceps* (Marette et al. 1993). Cell surface α_2_ and β_1_ abundance was later confirmed in both *m. soleus* and *m. gastrocnemius (white)* (Lavoie et al. 1997). They then quantified α and β molar contents in rat red muscle finding the α_1_, α_2_, β_1_ and β_2_ isoforms were 1.6–3.3 times more abundant in surface than in internal membrane fractions and also indicated a clear excess of β subunits (Lavoie et al. 1997). They also demonstrated in rat muscles that insulin substantially increased α_2_ and β_1_ in plasma membrane fractions, consistent with reduced α_2_ in internal membranes, suggesting that insulin caused trafficking of α_2_ and β_1_ from different intracellular pools to the plasma membrane (Hundal et al. 1992; Marette et al. 1993; Lavoie et al. 1996). They suggested that the high plasma membrane abundance and unresponsiveness of α_1_ to insulin was compatible with a “house-keeping” role for α_1_ as regulating Na^+^/K^+^ ion transport in muscle (Hundal et al. 1993). Furthermore, insulin increased surface membrane α_2_ and β_1_ only in *m. soleus* but not in *m. gastrocnemius (white)* (Lavoie et al. 1996). Insulin-induced translocation of NKA α_2_ (but not α_1_) to the plasma membrane was later shown in rat *m. soleus*, also with greater NKA activity in isolated cell surface membranes and with reversible phosphorylation of α_1_ and α_2_ (Chibalin et al. 2001). Then, using surface biotinylation, they detected translocation of both α_1_ (51%, 73%) and α_2_ (74%, 97%) to the plasma membrane with insulin, in rat epitrochlearis muscle and in human muscle cell cultures, respectively (Al-Khalili et al. 2003). In summary, most of these studies reported greater abundance of NKA isoforms, especially a_2_, in plasma membrane than in internal membranes in muscle and further showed that insulin induced translocation of NKA isoforms from internal to plasma membranes, which occurred to a greater extent in oxidative than glycolytic muscles.

**Fig. 5:**
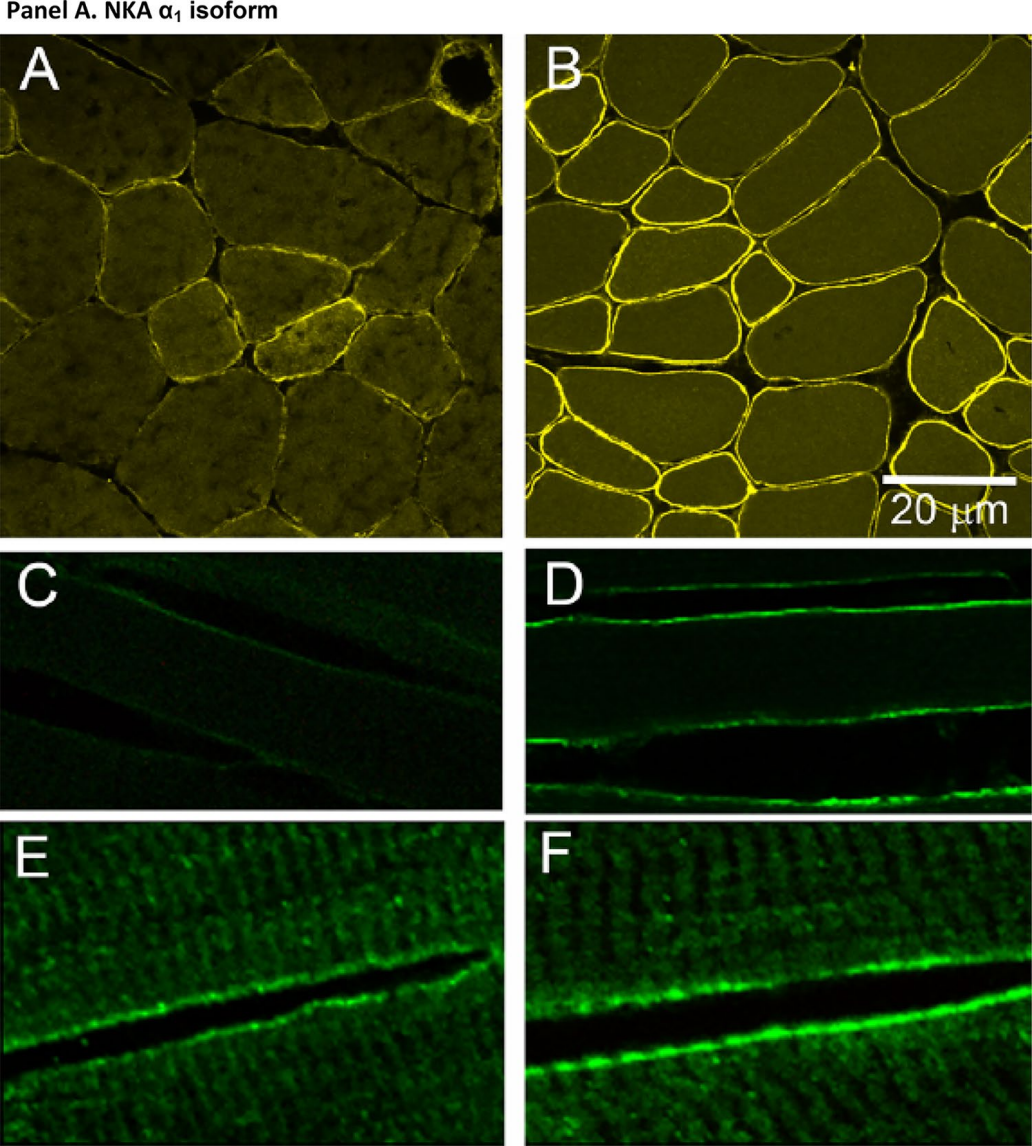

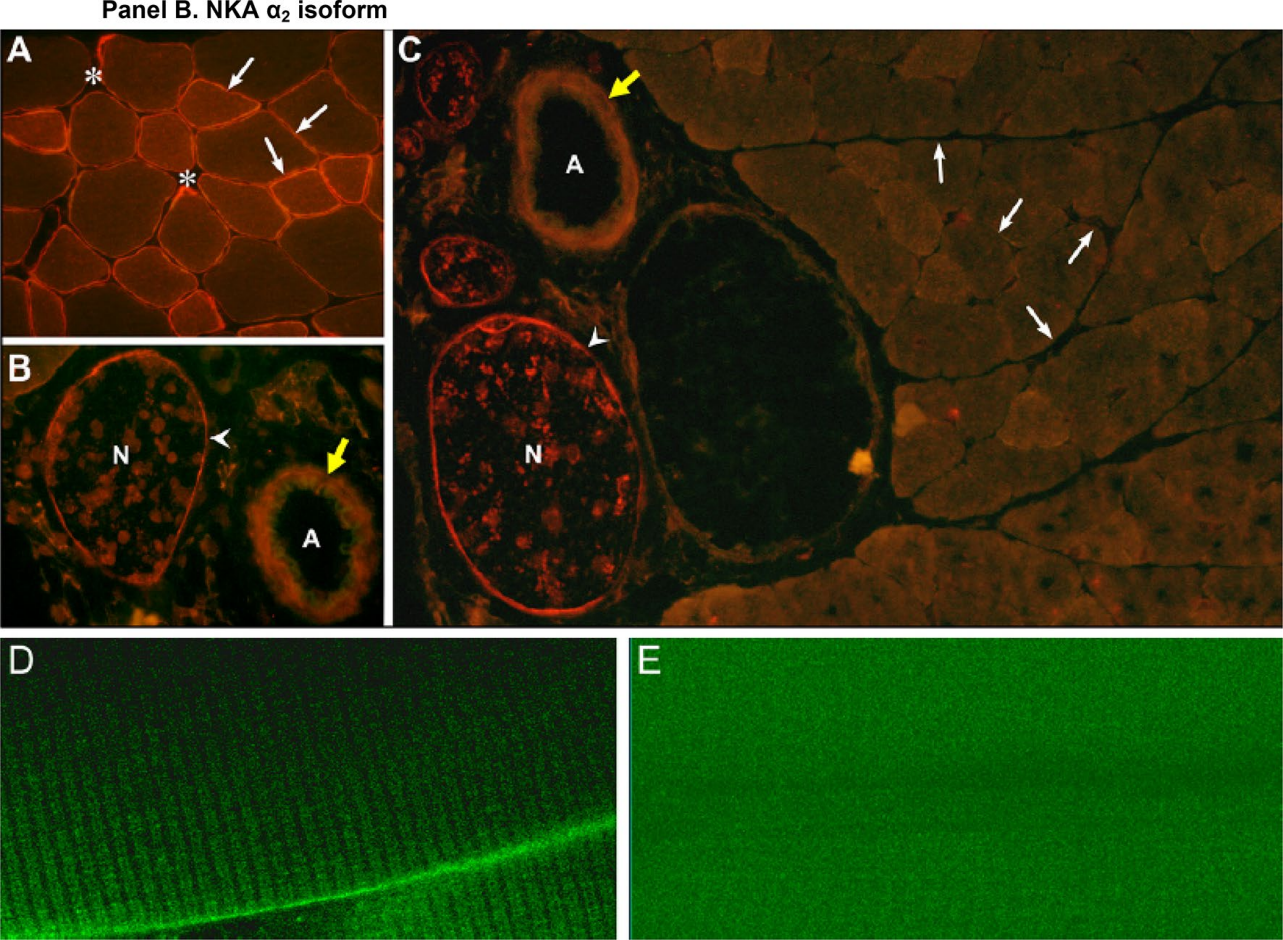
Fluorescence and confocal images of NKA α_1_ (Panel I) and α_2_ (Panel II) isoform expression and localization in *m.* *tibialis anterior* and *m. EDL*, in wild-type mice and in gene-targeted mice with deletion of NKA α_2_ isoform expression in Skeletal muscle (skα_2_^(−/−)^). From Figs. 4 and 3, respectively, in Radzyukevich et al. (2013) (with permission). Panel I: Transverse sections of murine *m*. *tibialis anterior* (**A**, **B**) and longitudinal scans of *m. EDL* (C-F) labelled for NKA α_1_ isoform, in wild-type (**A**, **C**, **E**) and in gene-targeted skeletal muscle α_2_ deletion (skα_2_^(−/−)^) mice (**B**, **D**, **F**). Images show sarcolemmal and t-tubular location of α_1_ in wild-type mice, with enhanced α_1_ abundances in skα_2_^(−/−)^ mice. Panel II: Transverse sections of murine *m*. *tibialis anterior* (**A**, **B**, **C**) and longitudinal scans of *m. EDL* (**D**, **E**) labelled for NKA α_2_ isoform, in wild-type (**A**, **B**, **D**) and in gene-targeted skeletal muscle α_2_ deletion (skα_2_^(−/−)^) mice (**C**, **E**). Images show sarcolemmal (image **A**, designated by arrows) and t-tubular locations (**A**, **D**) of α_2_ in wild-type mice, with absence of α_2_ in muscle fibres in skα_2_^(−/−)^ mice (**C**, **E**), although with α_2_ presence retained in motor nerves and arteriolar smooth muscle (images B and C, labelled as (small font) “N” and “A” with accompanying arrow head and arrow)

Using immunofluorescence longitudinal scans in *m. EDL* in rat and in mice, each of NKA α_1_ and α_2_, β-spectrin and ankyrin-3 were co-distributed in a rectilinear, “costameric” lattice on the plasma membranes, concentrated over Z- and M-lines, with their co-association confirmed by co-immunoprecipitation analyses. In transverse sections of mouse *m. EDL*, both α_1_ and α_2_ were present in the sarcolemma but only α_2_ in t-tubules, which was confirmed using isolated t-tubular and sarcolemmal membrane fractions (Williams et al. 2001). Contrasting, specific locations of NKA α_1_ and α_2_ isoforms were clearly demonstrated in cross- and longitudinal-sections of *m. EDL*, with α_1_ mainly located in the surface sarcolemma, but also found in t-tubules, possibly at superficial regions and/or low abundance, α_2_ present in t-tubules and the sarcolemma, including the motor end plate, caveolae and costameres, as well as the sheath surrounding the muscle spindle and with α_2_ also detected in motor nerve axons, perineurium and arterial smooth muscle (Radzyukevich et al. 2013). Confocal imaging of longitudinal-sections indicated α_2_ in sarcolemma and also in t-tubules evident as double rows per sarcomere (Radzyukevich et al. 2013) (Fig. 5). The α_2_ in t-tubules were functionally important in rapidly responding to elevated t-tubular [K^+^] from 4 to 40 mM (DiFranco et al. 2015).

In summary, studies in rats and mice using muscle membrane fractionation, immunogold and immunofluorescence approaches all demonstrated an abundance of α_1_ in plasma membranes and of α_2_ in t-tubular membranes, with immunofluorescence studies demonstrating additional detection of α_2_ in plasma membranes and of α_1_ in t-tubular membranes and also α_2_ located in costameres and other sub-cellular structures. Insulin increased α_2_ abundance in plasma membranes, which suggested that NKA α_2_ translocation was important in enabling increased NKA activity, but corresponding intracellular changes were inconsistent. Use of [^3^H]-ouabain binding and de-tubulation indicated that the majority of NKA were present in the sarcolemma.

#### NKA isoform muscle-specific expression

Another fundamental question addressed was whether NKA isoform expression in animal muscles varies between different muscle fibre types. Striking phenotypical differences between red and white muscles were found for β_1_ and β_2_, but not α_1_ or α_2_ in rats, with β_1_ abundance in a plasma membrane fraction ~ fivefold higher in pooled red than in white muscles and conversely, with β_2_ abundance in plasma and internal membrane fractions ~ threefold higher in white than in red muscles (Hundal et al. 1993). In rat hindlimb muscles, immunogold electron microscopy analyses in rat hindlimb muscles indicated 38% higher α_2_ abundance at the cell surface in white than red muscles (Lavoie et al. 1996). Subsequently, α_1_ and α_2_ were found in all muscles, with α_1_ and β_1_ abundance two–fourfold higher in oxidative than in glycolytic muscles, α_2_ abundance relatively high in all muscles, with β_2_ not detected in oxidative muscles and highest in fast glycolytic muscles and with α_3_ not detected in any muscle (Thompson and McDonough 1996). They suggested that the α_2_β_2_ heterodimer is predominant in fast-twitch glycolytic muscle with both α_2_β_1_ and α_2_β_2_ heterodimers expressed in muscles rich in oxidative fibres and with tissue-specific downregulation of NKA α_2_ and β_2_ with hypokalaemia to help preserve extracellular [K^+^] (Thompson and McDonough 1996; McDonough and Youn 2005; McFarlin et al. 2020). The ratio of NKA isoform abundances in rat sarcolemmal giant vesicles between pooled oxidative compared to glycolytic muscles for α_1_, α_2_ and β_2_ was 2.4, 1.6 and 0.8, respectively, with β_1_ found almost exclusively in oxidative muscles (Juel et al. 2001). In rats, α_1_ and β_1_ were greater in *red* than in white muscles, whereas differences were less marked for α_2_ and β_2_ (Fowles et al. 2004), and in mice, α_1_ and α_2_ were greater in *m. flexor digitorum brevis (m. FDB)* than in *m. EDL* (Ammar et al. 2015). Recently, lesser α_1_ abundance was found in more glycolytic muscles in mice, whereas differences in α_2_ were not proportional to glycolytic activity (Kutz et al. 2018). Comparisons of isoform protein abundances between different muscles are shown in Table 4. Whilst these comparisons for a given isoform were only expressed relative to that in other muscle(s), two studies have quantified NKA α_1_ and α_2_ contents in different muscles. In rats, α_1_ content determined by immunoblotting and radiography was only ~ 15–25% of total muscle NKA, being similar in *m. soleus* and *m. EDL* in 4 week old rats (135–220 pmol g^−1^) and with both lower in adults (~ 70 to 80 and 40–60 pmol g^−1^, respectively) (Hansen 2001). In *m. EDL* in mice, α_1_ and α_2_, respectively comprised 87 and 13% of the total α isoforms, when determined using an antibody recognising an epitope common to all α isoforms (He et al. 2001).

**Table 4:** Historical comparisons of immuno-detection of NKA isoform protein relative abundances between different muscles in rats and mice

References	Species	Muscles compared (in order shown from first red relative to last white muscle)	Preparation	NKA isoform	Ratio(s)^a^
Hundal et al. (1993)	Rat	Pooled red: pooled white muscles, comprising *m. soleus*, *m. gastrocnemius (red)* and *m. quadriceps (red)*: *m. gastrocnemius (white)* and *m. quadriceps (white)*	SL fraction IC fraction	α_1_	~ ~
		As above	SL fraction IC fraction	α_2_	~ ~
		As above	SL fraction IC fraction	β_1_	5: 1 ~
		As above	SL fraction IC fraction	β_2_	1: 3 1: 3
Thompson and McDonough (1996)	Rat	*m. diaphragm: m. soleus*: *m. gastrocnemius (red)*: *m. gastrocnemius (white): m. EDL* ^b^	Homogenate	α_1_	4.3: 3.3: 1.7: 0.8: 1
		As above	Homogenate	α_2_	1.4: 0.7: 1.1: 0.6: 1
		As above	Homogenate	β_1_	2.0: 2.5: 1.5: ND: 1
		As above	Homogenate	β_2_	ND: ND: 0.8: 1.3: 1
Lavoie et al. (1996)	Rat	*m. soleus*: *m. gastrocnemius* (*white*) (immuno-gold labelling)	Ultrathin cryosection	α_2_	1: 1.4
Juel et al. (2001)		Pooled oxidative: pooled glycolytic muscles, comprising *m. soleus, m. vastus intermedius, m. gastrocnemius (red)*: *m. vastus lateralis (white), m. gastrocnemius(white)* and* m. tibialis anterior(white)*	SL (giant vesicle)	α_1_	2.4: 1
		As above	SL	α_2_	1.6: 1
		As above	SL	β_1_	> 30: 1
		As above	SL	β_2_	0.8: 1
Fowles et al. (2004)	Rat	*m. soleus*: *m. gastrocnemius (red): m. EDL: m. gastrocnemius (white)*	Homogenate	α_1_	6.7: 4.3: 1.7: 1
		As above	Homogenate	α_2_	1.2: 1.4: 1.4: 1
		As above	Homogenate	β_1_	2.0: 1.3: 1.2: 1
		As above	Homogenate	β_2_	0.4: 0.9: 0.8: 1
		*m. soleus*: *m. gastrocnemius (red): m. EDL: m. gastrocnemius (white)*	Crude membrane	α_1_	2.5: 1.4: 0.9: 1
		As above	Crude membrane	α_2_	1.3: 1.2: 1.0: 1
		As above	Crude membrane	β_1_	50: 35: 18: 1
		As above	Crude membrane	β_2_	0.1: 0.2: 0.7: 1
Ammar et al. (2015)	Mouse	*m. FDB: m. soleus: m. diaphragm: m. EDL*	Homogenate lysate	α_1_	3.2: 2.7: 2.8: 1
		As above	Homogenate lysate	α_2_	1.6: 1.4: 1.1: 1
Kutz et al. (2018)	Mice	*m. soleus: m. gastrocnemius (red): m. gastrocnemius (white): m. plantaris: m. EDL*	Homogenate lysate	α_1_	20: 10: 5: 4: 1
		As above	Homogenate lysate	α_2_	2.5: 1.8: 0.3: 1.3: 1

~ no difference found, *ND* not detected, *SL* sarcolemma, *IC* intracellular

^a^When relative abundances of multiple muscles were compared and details provided, all ratios are included together. Ratios rounded to one decimal place

^b^Rat muscle fibre types cited as approx: *m. soleus*, 87% slow oxidative fibres, with some fast glycolytic-oxidative fibres; red gastrocnemius, a mixed muscle type, 30% slow oxidative fibres, 62% fast glycolytic-oxidative and 8% fast glycolytic; *m. EDL*, a classically fast muscle type, both fast glycolytic-oxidative (42%) and fast glycolytic (56%), with only 2% slow oxidative fibres; *m. gastrocnemius (white)*, very fast glycolytic muscle (84%) with some fast oxidative fibres; and *m. diaphragm*, a mixed muscle type, approximately 40% slow oxidative, 27% fast glycolytic-oxidative, and 34% fast oxidative (Thompson and McDonough 1996)

In summary, NKA isoform protein abundances differ between muscles in rats and mice, although the relative differences between muscles varied. All muscles contain α_1_ and α_2_ isoforms, with α_1_ and to a lesser extent also α_2_ having greater abundance in oxidative than glycolytic muscles, whereas β_1_ abundance was greater in oxidative and β_2_ higher in glycolytic muscles. A significant limitation was that these conclusions were based only on relative comparisons, whilst molar quantifications indicated that α_1_ comprised only ~ 15 to 25% of the total NKA α isoforms in both oxidative and glycolytic muscles.

#### Contraction-induced translocation of NKA isoforms to surface membranes in muscle

An intriguing question addressed over the past quarter century was whether exercise or muscle contractions can induce translocation of NKA isoforms from intracellular sites to the plasma membrane, with five studies providing evidence in support of NKA translocation in muscle in rats, although methods and findings varied (Tsakiridis et al. 1996; Juel et al. 2001; Sandiford et al. 2005; Kristensen et al. 2008; Rasmussen et al. 2008). After 1 h running, α_1_ and α_2_ abundances were increased in a purified plasma membrane fraction in both mixed red and white hindlimb muscles, but without any changes in α_1_ or α_2_ in a purified intracellular membrane fraction, whilst β_1_ or β_2_ were unchanged in all preparations (Tsakiridis et al. 1996). They concluded that additional α subunits could be recruited to plasma membranes during exercise, but limitations included the lack of reciprocal changes in plasma and internal membrane pools, as well as the very low membrane yield and small sample size. After 1 h low-intensity intermittent treadmill running, each of α_1_, α_2_, β_1_ and β_2_ were increased in sarcolemmal giant vesicles from oxidative muscles (by ~ 19, 32, 27 and 25%, respectively), with α_1_, α_2_ and β_2_ increased in glycolytic muscles (~ 22, 25 and 13%, respectively) with these changes reversed post-exercise and with binding of [^3^H]-ouabain in *m. soleus* also increased (~ 30%) (Juel et al. 2001). Intense electrical stimulation of *m. soleus* that reduced force by 80% increased α_1_ and β_1_ (22% and 18%, respectively), with no change in α_2_. They concluded that translocation of NKA isoforms occurred with exercise and were reversible in recovery, but noted the yield of their method was only 0.3% of the total NKA in muscle (Juel et al. 2001). Stimulation of *m. soleus* for 90 min increased α_1_ abundance in homogenates by 15% with no changes in α_2_ or β_1_, whilst in a sarcolemmal fraction, α_1_ and α_2_ were increased (14% and 40%, respectively), whereas in an endosomal fraction, α_1_ and α_2_ were decreased after 15 min stimulation (27% and 42%, respectively), with α_1_ increased (29%) after 90 min and with β_1_ unchanged in both fractions (Sandiford et al. 2005). Further, after 90 min stimulation, the homogenate [^3^H]-ouabain-binding site content was also 16% greater than controls, with 3-*O*-MFPase activity increased by 53% in a homogenate and by 40% in a sarcolemmal fraction. They concluded that NKA α isoforms were translocated to sarcolemmal membranes and contributed to the observed increase in NKA activity (Sandiford et al. 2005). After intense intermittent running, α_2_ was increased by 41% in sarcolemmal giant vesicles and by 36% in an enriched outer membrane fraction (2.1–2.4% protein recovery), along with a 37% increase in 3-*O*-MFPase activity, from pooled mixed muscles (Kristensen et al. 2008). After stimulation of *m. soleus* and cell surface biotinylation, α_2_ was increased by 40%, with caveolin-3 abundance increased by ~ 19% after exercise and stimulation (Kristensen et al. 2008). They concluded that NKA α_2_ can be translocated from caveolae and from intracellular sites to the plasma membrane by muscle contractions. They separately also reported that treadmill running increased NKA α abundances and NKA activity (Na^+^-stimulated ^32^P-ATP hydrolysis) in giant vesicles from mixed hindlimb muscles (53% and 67%, respectively) and in an enriched outer membrane fraction from mixed muscles (both by 33%) and concluded that translocation of α isoforms directly contributed to increased NKA activity in exercised muscle (Rasmussen et al. 2008).

In summary, considerable evidence has accumulated in favour of NKA α_2_ translocation in muscle after exercise and induced contractions, primarily obtained utilising purified membrane preparations, showing increases in sarcolemmal preparations and reductions in intracellular preparations, as well as cell surface biotinylation. Uncertainty remains because of inconsistencies in the actual isoforms involved, the detection and reciprocity of gains/declines of isoform abundances in these fractions, differences between exercise and electrical stimulation, low protein yields of purified fractions and small sample sizes. Further work is required to unequivocally support translocation of NKA to the surface membrane with muscle contractions, but would be extremely beneficial to increase muscle NKA activity (Benziane and Chibalin 2008), reduce intracellular K^+^ loss, preserve *E*_m_ and therefore contribute to minimising muscle fatigue (Renaud et al. 2023).

#### Effects of training and inactivity in animals on muscle NKA isoforms

Numerous studies have demonstrated upregulation of NKA isoforms with training in animals, but differ greatly in the magnitude of responses between muscles, types of training and animal models used. In rats with surgically induced myocardial infarction, endurance training for 6–8 weeks increased both α_2_ and β_2_ in *m. gastrocnemius (red)*, but not in *m. gastrocnemius (white)* (Helwig et al. 2003). In senescent rats, endurance training for 13–14 weeks increased α_1_ and α_2_ in *m. gastrocnemius (red)* (15, 73%, respectively), α_2_ in *m. gastrocnemius (white)* (89%) and *m. EDL* (34%), β_1_ in all three muscles (by 2–3-fold), but reduced β_2_ and β_3_ in *m. gastrocnemius (white)* (64, 49%, respectively) and β_3_ in *m. gastrocnemius (red)* (67%) (Ng et al. 2003). In rats fed a chow diet, 5 day swim training did not alter α_1_, α_2_, or β_1_ but reduced β_2_ (45%) in *m. gastrocnemius (white)*, whilst rat fed a high fat diet for 4 weeks had an initial elevation in α_1_ (50%) and reductions in both α_2_ (50%) and β_1_ (52%), that were each normalised after training (Galuska et al. 2009). Thus, in rats, diet affected the NKA isoforms in muscle and training normalised these changes. In horses, 18 weeks of combined interval and endurance training increased α_2_ in *m. vastus lateralis* and *m. pectoralis descendens* (2.2- and 1.5-fold, respectively) and also β_1_ (1.7-fold) in *m. vastus lateralis* (van den Burg et al. 2009). Finally, after sprint interval training for 3 days, increases were found relative to controls in *m. soleus,* for α_1_ (trained 41% increase compared to control 15% reduction, net increase 56%), α_2_ (net increase 101%) and β_1_ (net increase 31%), with no changes evident after 3 weeks training, whilst in *m. EDL*, α_1_ was increased after 3 days (net increase 58%), α_2_ and β_1_ were unchanged and β_2_ abundance reduced (38%) (Rasmussen et al. 2011). After endurance training, no differences were seen after training for any isoform in *m. soleus*, or in *m. EDL* except for reduced β_2_ after 3 day and 3 weeks training (27 and 64%, respectively). Thus, considerable differences were seen between studies on NKA isoform adaptability, with very large and inconsistent increases reported in isoforms and including that increased α_2_ was also not always found after training, as expected by the 20–40% increases in [^3^H]-ouabain-binding site content found after training.

Reductions in [^3^H]-ouabain-binding site content in animal muscles after inactivity (Sect. Inactivity) infers corresponding α_2_ downregulation, but few studies have examined NKA isoform changes with inactivity in animal muscles. Many studies used cage-bound rats as controls, which enforce sedentary behaviour and display ~ 20% lower α_1_ in both *m. soleus* and *m. EDL* compared to rats that undertook voluntary wheel running for 12 weeks (Xu et al. 2018). This suggests that α_1_ is sensitive to chronic reductions in activity levels in rats and also that a component of the training responses in earlier studies in rats was simply due to restoration of their normal daily activity. Several studies recently demonstrated early, localised changes in α_2_ (but not α_1_) and *E*_m_ in *m. soleus* after inactivity induced via short-term hindlimb suspension in rats (Kravtsova et al. 2015, 2016; Kravtsova and Krivoi 2021; Petrov et al. 2017). The α_2_ was unchanged after 6 h, increased by 150% after 12 h, by 125% after 24 h, primarily at extrajunctional membranes (caveolae and t-tubules), but did not differ from control by 72 h. Resting *E*_m_ was slightly depolarised at each time, primarily due to small reductions in the electrogenic contribution of α_2_ to the *E*_m_, at both junctional and extrajunctional regions. Hence, NKA α_2_ and associated E_m_ are differentially regulated in the early hours after hindlimb suspension.

#### NKA isoform-specific Na^+^ and K^+^ affinities

The affinity of NKA isoforms for Na^+^ and K^+^ determines their binding at intracellular and extracellular sites and thus also modulates NKA activity. In non-muscle tissues, different NKA αβ isoform complexes display an apparent affinity for Na^+^ (*K*_0.5_) ranging from 8.8 to 27.9 mM and for K^+^ (*K*_0.5_) from 1.9 to 6.2 mM (Blanco and Mercer 1998). In rat muscle, the affinities for Na^+^ and K^+^ were higher (i.e. lower *K*_m_) in oxidative than glycolytic fibres and treadmill running reduced the *K*_m_ for Na^+^ in *m. vastus lateralis (white)* which removed the difference between fibre types and increased *K*_m_ in oxidative fibres; exercise did not affect the *K*_m_ for K^+^ (Juel 2009). The Na^+^ and K^+^ affinities of human NKA isoforms were measured after αβ complexes were expressed in *Xenopus* oocytes, with the apparent affinity for Na^+^ (*K*_0.5_) dependent on the α isoform, in the order α_1_β_1_ ≥ α_2_β_1_ > α_3_β_1_ (8.3 to 24.7 mM), whilst the apparent affinity for K^+^ (*K*_0.5_) for αβ complexes ranged from 0.92 to 2.70 mM (Crambert et al. 2000). In mouse *m. flexor digitorum brevis* fibres, the K_0.5_ for K^+^ for α_2_ was ~ 4 mM, which implies saturation in the [K^+^]_e_ range of 20–40 mM and with its abundant t-tubular location, means that α_2_ can respond rapidly to elevated [K^+^] within the t-tubules (DiFranco et al. 2015). In contrast, K_0.5_ values for K^+^ for α_1_ of ~ 1 to 2 mM indicate that α_1_ operates above its K_0.5_ at resting [K^+^]_e_ and thus likely primarily contributes to Na^+^/K^+^ exchange and membrane potential whilst the muscle is at rest. Hence, they proposed the α_1_ was responsible for these roles in quiescent muscle, whereas α_2_ provides a reserve capacity for rapid NKA activation in contracting muscles.

In summary, the NKA affinities for Na^+^ and K^+^ were higher in oxidative than glycolytic fibres and also vary between different αβ complexes, which affect NKA activity. Different affinities of α_1_ and α_2_ for K^+^ also enable specific NKA αβ complexes to function throughout the physiological range of [K^+^]_e_, with α_1_ complexes proposed to be primarily active under conditions of low [K^+^]_e_ at rest and in recovery and α_2_ complexes during contractile activity when [K^+^]_e_ is substantially elevated.

#### Genetic manipulation of NKA α isoforms in muscle and their functional implications

A major development was the use of gene targeting to investigate different physiological roles of NKA α_1_, α_2_ and α_3_ isoforms in mice, developing animals with a global knockout, lacking one allele of the NKA genes (Lingrel et al. 2003; Kutz et al. 2018; He et al. 2001), as well as targeted gene deletions of the α_2_ isoform in skeletal muscle (Radzyukevich et al. 2013; Manoharan et al. 2015). Global knockouts revealed each of the α isoforms were essential for survival, with complete knockout of α_1_ and α_3_ non-viable and α_2_ global knockout pups either born dead or dying within a few minutes after birth (Lingrel et al. 2003; Moseley et al. 2007). In contrast, animals lacking one allele of the α_1_, α_2_ or α_3_ genes were viable and fertile but demonstrated isoform-specific behavioural changes, including in locomotor activity (Lingrel et al. 2003; Moseley et al. 2007).

In mice lacking one copy of α_1_ (α_1_^+/−^) or of α_2_ (α_2_^+/−^), the α_1_ and α_2_ abundances were correspondingly reduced by 48% and 46% in *m. EDL*, where force was reduced by 20% in α_1_^+/−^ mice, but increased by 2% in α_2_^+/−^ mice (He et al. 2001). A compensatory 39% increase in α_1_ was found in the α_2_^+/−^ mice, whereas β isoforms, Na^+^_c_ and K^+^_c_ and fatigue-induced reductions in force were unchanged in both mouse models. In α_2_ heterozygous (α_2_^+/−^) and α_2_ knockout (α_2_^−/−^) mice, the perinatal *m. diaphragm* α_2_ was decreased by 38% and absent, respectively, with substantial compensatory α_1_ upregulation of 47% and 94%, respectively (Radzyukevich et al. 2004). Importantly, in the α_2_^−/−^ mice, they found that the *m. diaphragm* was capable of maintaining near-normal *E*_m_, AP’s and force, including during fatiguing contractions, although a reduced ability to sustain trains of AP’s was found. These findings provided strong evidence for the role of α_1_ in NKA “housekeeping” functions of maintaining Na^+^/K^+^ gradients and *E*_m_. In mice where NKA α_2_ was specifically knocked out in skeletal muscle (skα_2_^−/−^), despite a 2.5-fold compensatory increase in α_1_, running speed and capacity during an incremental test were markedly impaired, the *m. EDL* was more fatigable in-vivo as well as in vitro, with twitch and maximal force reduced by 24–54% in *m. EDL* and *m. soleus* which was also more fatigable than in wild type mice (Radzyukevich et al. 2013). Resting muscle *E*_m_ did not differ between wild type and skα_2_^−/−^mice, consistent with other findings (Ammar et al. 2015). These findings suggest that α_2_ only played a small role in *E*_m_ maintenance in resting muscle, but is essential for locomotor activity, provided a reserve capacity for Na^+^/K^+^ transport during muscle contractions and was essential for resisting fatigue that might occur due to K^+^ build up in t-tubules. The greater fatiguability in these skα_2_^−/−^ animals was consistent with lacking any α_2_ in t-tubules and sarcolemma. Recently, in α_1_ haplo-deficient/heterozygous (α_1_^+/−^) mice, α_1_ was reduced by 30–40% in *m. soleus*, *m. plantaris* and *m. EDL*, without any compensatory increase in α_2_, changes in NKA activity or running performance (Kutz et al. 2018). However, the *m. soleus* mass was reduced by 9% in α_1_^+/−^ mice, indicating that α_1_ was important for maintaining *m. soleus* growth, suggested to be due to cardiotonic steroid-induced intracellular signalling (Xie and Askari 2002).

In summary, studies with genetically modified mice strongly support different roles and locations of α_1_ and α_2_ isoforms in skeletal muscle. The α_1_ is located primarily in the sarcolemma with lesser t-tubular abundance and plays a key role in maintaining resting muscle Na^+^/K^+^ exchange and *E*_m_, as well as growth in oxidative muscles, but with little specific role during muscle contractions. In contrast, the α_2_ isoform is located primarily in the t-tubules with lesser abundance in the sarcolemma, has little role in resting muscle, but plays a key role in Na^+^/K^+^ exchange, *E*_m_ and fatigue resistance during stimulated muscle contractions and exercise.

#### FXYD expression in skeletal muscle in animals at rest and with exercise

The earlier described γ-subunit of NKA was later designated as FXYD2, a member of the FXYD family that comprises seven family members (FXYD_1-7_) and includes FXYD1, originally named phospholemman (Sweadner and Rael 2000). The FXYD family are small, single-span membrane proteins associated with NKA, with FXYD1 mainly expressed in skeletal muscle and heart (Geering et al. 2003; Geering 2005, 2006). The tissue distribution, interactions with NKA and physiological implications of individual FXYD proteins are covered elsewhere (Garty and Karlish 2006; Yap et al. 2021).

FXYD1 was first identified in skeletal muscle sarcolemmal membrane fractions as a 15 kDa peptide that was phosphorylated by insulin (Walaas et al. 1977) and later detailed in muscle (Walaas et al. 1988) and in cardiac membranes (Palmer et al. 1991). In rat muscles (not specified) FXYD1 was associated with NKA α_1_ but not α_2_ and reduced apparent affinity for intracellular Na^+^ and for K^+^, thus being an important regulator of NKA activity (Crambert et al. 2002). Others have also shown NKA regulation via FXYD phosphorylation increasing Na^+^ affinity (Bibert et al. 2008; Cirri et al. 2011). Insulin, adrenaline, cAMP, electrical stimulation and exercise all increase FXYD1 phosphorylation in muscle, which likely plays a vital role in regulating NKA activity in muscle, including via increasing NKA affinity for Na^+^ (Pirkmajer and Chibalin 2016). Whilst FXYD1 interaction with NKA inhibits NKA activity and decreases Na^+^ affinity, FXYD1 phosphorylation relieves this inhibition and increases Na^+^ affinity, which allows protection against cellular Na^+^ overload (Yap et al. 2021). Hence, increased FXYD1 abundance and particularly phosphorylated FXYD1 in muscle, or specifically in plasma membranes due to translocation, enable increased overall increased capacity of muscle to regulate [Na^+^]_i_ and [K^+^]_i_.

In rats, FXYD1 abundance was ~ 15% higher in *m. EDL* than in *m. gastrocnemius (red)*, was primarily present in the sarcolemma, was associated with both α_1_ and α_2_ and an anti-FXYD1 antibody reduced NKA activity by more than 50%, indicating that FXYD1 modulates NKA activity (Reis et al. 2005). The FXYD1 abundance was similar in *m. EDL* and *m. soleus* in rats (Rasmussen et al. 2008). Treadmill running in rats increased FXYD1 by 203% in sarcolemmal giant vesicles and by 344% in an outer membrane-enriched fraction, prepared from mixed muscles, without change in phosphorylation of Serine^68^ (Rasmussen et al. 2008). This increased FXYD1 in plasma membranes was attributed to translocation of FXYD1 and α subunits, with an increased association between FXYD1 and α_1_ also seen and proposed to partially contribute to increased muscle NKA activity after exercise. In *m. soleus*, FXYD1 was located in the sarcolemma and throughout the fibres and immunoprecipitation indicated FXYD1 were associated with around 30% NKA α_1_ and α_2_ isoforms. Subsequently, whilst an acute bout of exercise did not change FXYD1 in either *m. soleus* or *m. EDL*, 3 days of training increased FXYD1 after exercise in *m. soleus* and conversely reduced FXYD1 in *m. EDL* (Rasmussen et al. 2011). Contrary findings were obtained in FXYD1-knockout mice, which showed normal exercise capacity, fatiguability, α_2_ abundance, ouabain-inhibitable Rb^+^ uptake and furthermore, found that in vivo muscle contraction did not alter FXYD1 phosphorylation in muscle (Manoharan et al. 2015). They concluded that neither FXYD1 nor FXYD1 phosphorylation was required for normal muscle function with exercise.

In summary, FXYD1 is found in rat skeletal muscle, is associated with some NKA α_1_ and α_2_ isoforms and exercise increased its association with α_1_. Whilst acute exercise did not increase overall FXYD1 abundance in muscle, FXYD1 was increased in sarcolemmal membranes, possibly resulting from translocation from undetermined intracellular sites. Whilst an increased sarcolemmal FXYD1 abundance and association with α isoforms would contribute to increased sarcolemmal NKA activity, studies in FXYD1-knockout mice revealed that FXYD1 was not essential for NKA function. Exercise or muscle contractions do not appear to increase FXYD1 phosphorylation in rat or mouse muscle.

### NKA isoform and FXYD expression in human skeletal muscle

#### NKA gene expression in human muscle

Multiple NKA gene transcript variants were first detected in human muscle for α_1_, α_2_ and β_1_ (Nordsborg et al. 2003a), followed by detection of each of the NKA α_1_–α_3_ and β_1_–β_3_ gene transcripts (Murphy et al. 2004) and since confirmed (Nordsborg et al. 2005b; Murphy et al. 2006; Aughey et al. 2007; Perry et al. 2013). Detection of these transcripts also in human muscle cell cultures suggested that this expression was unlikely due to contamination by nervous tissue, adipocytes or leucocytes (Murphy et al. 2004). The α_1_ mRNA was 20-fold more abundant than of α_2_, β_1_ 100-fold more abundant than β_2_ and β_3_, whilst the α_3_ and α_4_ transcripts were present, but not at reliable detection levels (Nordsborg et al. 2005b). Recreationally active males had several-fold higher α_3_ and β_3_ mRNA expression than recreationally active females, whereas α_1_, α_2_, β_1_ and β_2_ mRNA did not differ (Murphy et al. 2007). Early studies in unspecified human muscle detected very low levels of β_3_ gene (Malik et al. 1998) and also α_4_ mRNA (Shamraj and Lingrel 1994; Keryanov and Gardner 2002). However, α_4_ is only abundantly present in sperm cells (Blanco and Mercer 1998) and was not detected in another study (Murphy et al. 2006). The NKA gene transcripts expressed in human muscle are summarised in Table 5.

**Table 5:** NKA isoform mRNA or protein expression in skeletal muscle in humans

References	*n*, sex (F, M)	Age (years)	Muscle (fibre type)	α_1_	α_2_	α_3_	α_4_	β_1_	β_2_	β_3_
mRNA										
Shamraj and Lingrel (1994)	nr	nr	nr				+			
Keryanov and Gardner (2002)	nr	nr	nr				+			
Malik et al. (1998)	nr	nr	nr							+
Nordsborg et al. (2003a)	6 M	25	v. lat	+	+			+		
Murphy et al. (2004)	7F,7 M	24	v. lat	+	+	+		+	+	+
Petersen et al. (2005)	7F,8 M	25	v. lat	+	+	+		+	+	+
Nordsborg et al. (2005a)	10 M	25	v. lat Deltoid	+ +	+ +			+ +	+ +	− + *
Nordsborg et al. (2005b)	8 M	24	v. lat	+	+	+	+ *	+	+	+
Murphy et al. (2006)	5F, 6 M	24	v. lat	+	+	+	-	+	+	+
Perry et al. (2013)	10F,9 M OA 8F, 9 M	70 70	v. lat	+ +	+ +	+ +		+ +	+ +	+ +
Christiansen et al. (2018a)	19 M	24	v. lat	+	+	+		+	+	+
Protein^a^										
Hundal et al. (1994)	5, nr PVD	nr	Soleus	+	+	+		+	-	
Murphy et al. (2004)	7F, 7 M	24	v. lat	+	+	+		+	+	+
Murphy et al. (2006)	5F,6 M	24	v. lat	+	+	+		+	+	+
Mohr et al. (2006)	13 M	26		+	+			+		
Thomassen et al. (2010)	18 M	23	v. lat	+	+			+		
Thomassen et al. (2013)	6 M	27	v. lat. Type I v. lat. Type IIA	+ +	+ +			+ +		
Petersen et al. (2012)	3F,7 M	40	v. lat	+	+	+		+	+	+
Wyckelsma et al. (2016)	8F,6 M 7F,10 M	26 69	v. lat. homog v. lat. Type I v. lat. Type IIA	+ + +	+ + +	+ + +		+ + +	+ + +	+ + +
Wyckelsma et al. (2017)	6F,9 M	69	v. lat. homog v. lat. Type I v. lat. Type IIA	+ + +	+ + +			+ + +		
Christiansen et al. (2018a)	19 M	24	v. lat. Type I v. lat. Type IIA	+ +	+ +	+ +		+ +	+ +	+ +

All references presented in chronological order. F, female, M, male; Age in mean years

Presence of NKA isoform detected (+), inconsistently detected (+ *) or not detected (−); blank cell indicates the transcript was not probed for

All analyses on healthy humans except where indicated as: OA, osteoarthritis; PVD peripheral vascular disease limb amputees, RTx, renal transplantation patients; HDP, haemodialysis patients; Muscle: v. lat., *m. vastus lateralis*; Type I, IIA Type I fibres and Type IIA fibres; homog., homogenate

^a^Only selected articles on NKA isoform protein expression are included here, for simplicity

#### NKA isoform protein abundances and their localisation in human muscle

NKA distribution in fast and slow twitch muscle fibres from patients undergoing surgery, comprised sarcolemmal distribution in transverse sections and in longitudinal sections, a cross-striation effect with NKA confined at the I-band, suggesting a t-tubular location (Benders et al. 1992). In *m. soleus* obtained from patients undergoing limb amputation, each of α_1_, α_2_, α_3_ and β_1_ were expressed and primarily located in a plasma membrane (96%, 58%, 88% and 74%, respectively, as percentage of total) compared to an internal membrane fraction, with β_2_ not detected (Hundal et al. 1994). Immunocytochemical analyses indicated that α_1_ was located in the surface membrane, whereas α_2_ was located at surface membranes and also diffusely distributed throughout the fibres. In healthy human *m. vastus lateralis*_*,*_ each of the α_1-3_ (molecular mass ~ 100 to 105 kDa) and β_1_-_3_ (~ 45 to 52 kDa) proteins were detected in homogenates, which enabled recovery of all NKA (Murphy et al. 2004). Thus, human muscle expresses each of the α_1_, α_2_, α_3_, β_1_, β_2_ and β_3_ isoforms, but their molar abundances and αβ complexes remain unknown.

Human muscle is heterogeneous with respect to fibre-type composition and fibre-type-specific approaches are recommended for analyses of intervention effects for NKA and proteins involved in contractile, metabolic, signalling and stress responses (Tobias and Galpin 2020). Recent studies compared the relative abundance of NKA isoforms in Type I and Type II single fibres and found few, or inconsistent, fibre type differences in NKA isoform expression (Thomassen et al. 2013; Wyckelsma et al. 2015, 2016, 2017; Christiansen et al. 2018a; Perry et al. 2016), in contrast to the differences typically seen in isoforms between different muscles in rat (Sect. NKA isoform muscle specific expression). Each of α_1_, α_2_ and β_1_, as well as FXYD1 were expressed in both Type I and Type II fibres, but with no differences between fibre types, except for 37% α_2_ higher in Type II fibres (Thomassen et al. 2013). A later investigation detected all α_1-3_ and β_1-3_ isoforms in both Type I and Type IIa muscle fibres, but with no fibre-type differences, except for β_2_ which was ~ 45% higher in Type IIa fibres (Wyckelsma et al. 2015). Higher α_3_ and β_2_ were found in Type IIa than Type I fibres, with no other fibre-specific differences detected (Wyckelsma et al. 2016). In contrast, a subsequent study found higher α_2_ (17%), β_1_ (62%) and β_2_ (54%) in Type II than I fibres and higher (35%) FXYD1 in Type I fibres, with no differences for α_1_, α_3_ and β_3_ (Christiansen et al. 2018a). No differences between fibre types were then found for α_2_, β_1_ and FXYD1 abundances and FXYD1 phosphorylation, but higher α_1_ (29%) was seen in Type I fibres in the control leg (Christiansen et al. 2019). Finally, no differences were found between Type I and IIa fibres for α_2_, β_1_ or for FXYD5, although glycosylated-β_1_ was higher in Type IIa fibres (Hostrup et al. 2023). Hence, there is no consensus on NKA isoform differences between fibre types in human muscle, with further studies clearly required, ideally using a large sample size comprising both men and women.

##### Ouabain, Na^+^ and K^+^ affinities

An early study reported that human muscle expressed ouabain-binding sites with two different affinities (Desnuelle et al. 1985), but others reported only small differences in ouabain sensitivity between nine different human NKA αβ complexes after expression in oocytes, with all αβ complexes exhibiting high affinity for ouabain (Crambert et al. 2000). The ouabain affinity was also measured in human α_1_, α_2_ and α_3_ isoforms in situ in skeletal muscle, finding that all three α isoforms had almost the same affinity for ouabain (Wang et al. 2001). Thus, human NKA affinity for ouabain does not differ between NKA complexes, supporting the use of ouabain binding as a measure of NKA content in human muscle. The Na^+^ and K^+^ affinities of different human NKA αβ complexes were also examined, finding that K^+^ affinity was lower in α_2_β_1_ than in α_2_β_2_ complexes, whilst the Na^+^ affinity was affected by the α isoform expressed, in the order α_1_β_1_ > α_2_β_1_ > α_3_β_1_ (Crambert et al. 2000).

##### Effects of acute exercise on NKA isoform gene expression

Intense knee extension exercise increased *m. vastus lateralis* α_1_ mRNA ~ threefold (Nordsborg et al. 2003a). Subsequently, brief, intense exercise was shown to elevate each of the α_1_, α_2_, α_3_, β_1_, β_2_ and β_3_ mRNA’s, when averaged over 0, 3 and 24 h post-exercise, with variable time courses of changes (Murphy et al. 2004). Increases in α_1_, α_2_, β_1_ and β_3_ mRNA were confirmed 0–5 h after intense knee extensor exercise (Nordsborg et al. 2005b), of α_1_, α_2_ and α_3_ mRNA after intense interval cycling (Aughey et al. 2007), α_1_ and β_3_ mRNA at 0 and 3 h after repeated 30 s maximal cycle sprints, whereas β_2_ mRNA was decreased (Christiansen et al. 2018a). In contrast, prolonged exhaustive cycling did not elevate the average post-exercise NKA mRNA for any isoform, although single post-exercise time-point increases were seen for α_1_, α_3_ and β_2_ mRNA (Murphy et al. 2006). Hence, although there is evidence that each of the NKA gene transcripts in human muscle can be increased with exercise, the extent and time-course of these effects are variable and the effects of exercise type, intensity and duration are not yet fully established. Nonetheless, this suggests that post-transcriptional regulation of NKA is important and likely plays a role in NKA adaptability in human muscle undergoing repeated bouts of exercise, i.e. training. The likely mechanisms underpinning these acute exercise effects on NKA mRNA in human muscle are recently discussed (Christiansen 2019).

##### Effects of acute exercise on NKA isoform protein abundances

Most studies that investigated acute exercise effects on the NKA isoform abundances in humans found no changes in α_1_, α_2_, α_3_, β_1_, β_2_ and β_3_ when measured in crude muscle homogenates, after each of brief, intense exercise (Murphy et al. 2004), prolonged, exhaustive exercise, except for an increase in α_3_ (Murphy et al. 2006), intense interval exercise (Aughey et al. 2007), or 2 h cycling at 60% *V*O_2peak_ (Green et al. 2011). Hence, acute exercise did not affect NKA isoform abundances in human muscle, although the effects in single fibres are not yet known. More extreme exercise over 16 h, comprising intense cycling for 6 min at 91% *V*O_2peak_ repeated each hour, did, however, increase muscle α_2_ (~ 26 to 30%) and α_3_ (~ 29 to 40%), but reduced β_3_ (~ 10%) protein (Green et al. 2007).

##### Effects of training and inactivity on NKA isoform protein abundances

As recently reviewed, the effects of training on the α_1_, α_2_ and β_1_ isoforms in human muscle are highly variable and inconsistent, contrasting robust findings of 8–22% increases in muscle NKA content after a range of training types (Wyckelsma et al. 2019). More recent training studies also show considerable differences in adaptability of NKA isoforms. Resistance training increased both α_1_ (32%), α_2_ (32%), with β_1_ and β_2_ unchanged (Altarawneh et al. 2020), whilst, in another study, induced large increases in α_2_ (70%) and β_1_ (78%), with these also similarly increased after low-load resistance training with restricted blood flow (Wang et al. 2023). High-intensity interval training for 6 weeks increased α_1_ (41%) and β_1_ (10%), but not α_2_ (Lemminger et al. 2022), did not change α_2_ or β_1_, but increased glycosylated β_1_ and lowered FXYD5 in Type IIa muscle fibres (Hostrup et al. 2023). Thus, further work is required to resolve these different outcomes in NKA isoform adaptability with training in humans. Studies examining the effects of reduced physical activity utilising bedrest, detraining, or unilateral limb suspension on human muscle NKA content and isoforms are few and their findings inconsistent (Wyckelsma et al. 2019).

##### FXYD expression in human muscle at rest and after exercise

FYXD1 mRNA was initially found to be expressed in high abundance in human muscle (unspecified) (Chen et al. 1997). FXYD1 mRNA was not affected by acute exercise comprising 4 × 30 s maximal sprints on a cycle ergometer (Christiansen et al. 2018a), or moderate-interval running, but was increased when running was performed with blood flow restriction (Christiansen et al. 2018b).

The effects of acute exercise on FXYD1 abundance and phosphorylation in human muscle are shown in Table 6. The FXYD1 protein was first detected in human *m. vastus lateralis* (Garvey et al. 1998), subsequently found to be widely expressed in human tissues (Floyd et al. 2010), and is the main isoform in muscle, although FXYD5 is also expressed (Boon et al. 2012). Only one study has reported an increased total FXYD1 abundance with acute exercise, being increased 19% after 5 min intense knee extension exercise (Thomassen et al. 2013). However, several studies have demonstrated that acute exercise can increase muscle FXYD1 phosphorylation status in humans, with differing methods and results (Benziane et al. 2011; Thomassen et al. 2011, 2013, 2016; Kalsen et al. 2016). One hour knee extension increased FXYD1 phosphorylation at Serine^63^ and Serine^68^ by 107% and 35%, respectively (Benziane et al. 2011), whilst combined intense and then submaximal exercise increased FXYD1 phosphorylation (32%) and specifically increased serine^63^ (43%), serine^68^ (26%) and combined serine^68^ and threonine^69^ (26%) phosphorylation (Thomassen et al. 2011). Brief intense knee extension exercise, increased the non-specific phosphorylated FXYD1 in both Type I (28%) and Type II fibres (46%), with serine^68^ phosphorylation also increased (90%) in Type II fibres (Thomassen et al. 2013). Intermittent exercise comprising short submaximal cycling then repeated intense exercise bouts, increased non-specific FXYD1 phosphorylation (100%), phosphorylation at FXYD1 Serine^68^ (~ 60%) and Thr^69^ (~ 150%) but not FXYD1 Serine^63^ (Thomassen et al. 2016). However, 30 s maximal sprint exercise did not change FXYD1 phosphorylation status (Kalsen et al. 2016). In summary, only one study has reported increased total FXYD abundance in muscle after acute intense exercise, whereas several have demonstrated increased FXYD phosphorylation at non-specific, Serine^63^, Serine^68^ and at Thre^68^ sites. An increased FXYD1 phosphorylation would be expected to increase the affinity for intracellular Na^+^ and contribute to increased muscle NKA activity (Pirkmajer and Chibalin 2016; Yap et al. 2021), suggesting that FXYD1 phosphorylation in muscle is also an important regulatory response to exercise in humans.

**Table 6:** Effects of acute exercise, training, reduced activity and inactivity/injury on FXYD1 protein abundance and phosphorylation in skeletal muscle in humans

References	*n*, sex (F/M)	Age	Exercise, train or inactivity details		FXYD1	FXYD1 phosphorylation				
Acute exercise			Exercise mode, type, intensity (%*V*O_2max_)	Dur min)	Total (%∆)	Non-specific (%∆)	Ser^63^ (%∆)	Ser^68^ (%∆)	Thre^69^ (%∆)	Ser^68^ + Thre^69^ (%∆)
Benziane et al. (2011)	8 M	23	CE, C, S; 1 leg 72% *V*O_2peak_	60	nr		↑107%	↑35%	nc	
Thomassen et al. (2011)	10 M	27	CE, C, HI; 166% *V*O_2max_ CE, C, S; 79% *V*O_2max_	0.5 20		↑16% ↑32%	nc ↑43%	nc ↑26%		nc ↑26%
Thomassen et al. (2013)	6 M	27	CE, C, Max; 95% *V*O_2peak_ Overall	5	↑19%					
			Type I, Type II fibres			↑28, ↑46%		nc, ↑90%		
Thomassen et al. (2016)	8 M	33	CE, Int; 6 min @ 50%, 70%, 70% peak PO 2 min 90%, to exh @ 90% peak PO (356W) (PreTrain Rest vs Exh)			↑100%	nc	↑ ~ 60%	↑ ~ 150%	
Kalsen et al. (2016)	13 M	32	CE, C, HI maximal sprint	0.5		nc				

Training, reduced activity or inactivity/injury			Mode, type of training/inactivity/injury details	(d/wk/mo)						
Thomassen et al. (2010)	7, nr 11, nr	23	FR. HIT, ↓ vol.; 5 × small sided soccer (84–88% HR_max_); SET: 4× (10–12 × 25–30 s all-out EB; 20 min); SET: 1 × 16 × 40–60 s EB;14 min	2 wk	nc			↑27%		
			Reduced Activity After final match season, maint. d. activities	2 wk	nc			↓18%		
Benziane et al. (2011)	9 M	23	CE, Aerobic + HIT; 6 d × 75% *V*O_2peak_, 45–90 min; 4 d × 6 × 5 min @ 95–100% *V*O_2peak_	10 d	nc		nc	nc	nc	
Boon et al. (2012)	6 M 7 M/1F 6 M	44 33 49	Inactive; chronic, complete cervical spinal cord injury		↓52%		nc	nc		
			Acute, complete cervical spinal cord injury	12 mo	~ ↓60%		nc	~ ↑30%		
			Acute, incomplete cervical spinal cord injury		nc		nc	nc		
Thomassen et al. (2016)	8 M	33	FR, HIT, ↓vol, (↓70%); SET: 2–3 dx 10–12 × 30 s all-out EB;20 min;	7 wk	↑30%	↑30%	nc	↑ ~ 90%	↑	
			Aerobic HIT 1-2d × 4–5 × 2 km run ~ 4 min, 90–95% HR_max_ (data shown as Pre Train Rest vs Post Train Rest, Ex: Pre Train Ex vs Post Train Ex)			Ex:↑ ~ 10–50%	nc	Ex:↑ ~ 35–53%	Ex:↑ ~ 39%	
Skovgaard et al. (2017)	8 M/3F 6 M/1F	29	FR SET: 20 sessions × 8–12 × 30 s all-out EB (high Freq 4 per 8 d; low freq 2 per 8 d); AM Aerobic moderate intensity train 30–60 min @ 60–80%HRmax	40 d 80 d	↑57% nc	↑46% nc				
Mohr et al. (2017)	21F 21F 21F	45	Swim/soccer; Train 3/wk. HIS 6–10 × 30 s all out swim,	15 wk	nc, nc					
			MOS 1 h max distance continuous swim;		nc,↑42%					
			SOC 1 h small-sided soccer games (data shown for Muscles: deltoid; v. lat.)		nc, nc					
Skovgaard et al. (2018)	8 M/3F	30/27	FR, Training High vol. SET: 4 sessions × 8–12 × 30 s all-out EB and 2 sessions AM train 30–60 min @ 60–85%HRmax every 8 d	40 d	↑ ~ 90%	nc				
			Tapering; SET 4 × and AM 3 × every 8 d. Post vs Pre	18 d	↑ ~ 50%	nc				
Fransson et al. (2018)	21 M 18 M	21	SET: 6 × 30 s all-out EB; Soccer: 2 × 7–9 min small-sided games 3x/wk added to normal training	4 wks	nc					

Blank cell indicates that variable was not measured. Participants: n number of participants; sex reported as F, female, M, male (n F/n M); age is reported mean years

Exercise details: Mode: CE, cycle ergometer, KE, knee extension, FR field/track running. All exercise conducted upright. Type: C, Continuous; Incr. incremental; Int, Intermittent; exercise intensity classified broadly as S, submaximal; Max, maximal (i.e. equivalent to *V*O_2max_); HI (high intensity at supramaximal workrate, i.e. exceeding *V*O_2max_). Intensity expressed as % (%*V*O_2max_) unless otherwise indicated as % HR_max_, or peak incremental Power Output (PO, W). Dur: exercise duration in minutes; Exh ~ exhaustion. Muscles: v.lat *m. vastus lateralis* unless otherwise specified. Biopsies usually taken at Rest or immediately after exercise (Ex); time in minutes

Muscle FXYD1 and phosphorylation status: reported as % change from Rest (End exercise vs Rest), using stated data or interpolated from Figures; ↑, increase, ↓, decrease; nc, no change (not significant); non-specific phosphorylation, reported as % change from inverse of phosphorylation antibody measure, see (Thomassen et al. 2011). Values not reported, nr

Training details: HIT high-intensity training, SET speed-endurance training, AM aerobic moderate intensity training

##### Effects of exercise training and inactivity on FXYD expression and phosphorylation in human muscle

Several studies demonstrated that training can increase FXYD1 abundance and/or phosphorylation in human muscle (Thomassen et al. 2010, 2016; Skovgaard et al. 2017, 2018; Mohr et al. 2017), whilst others found no effect (Benziane et al. 2011; Lemminger et al. 2022). Two weeks of high-intensity exercise training (HIT) elevated FXYD1 phosphorylation by 27% (Thomassen et al. 2010), whereas 10 days of combined aerobic training and HIT did not change total FXYD1 or the phosphorylation status (Benziane et al. 2011). Intensified training with reduced volume increased total FXYD1 by 30% and increased non-specific FXYD1 phosphorylation (30%), with greater increases during intense exercise after training in phosphorylation at Ser^68^ and Thre^69^ sites (Thomassen et al. 2016). After speed endurance combined with moderate intensity training, both FXYD1 abundance (57%) and FXYD1 non-specific phosphorylation (46%) were increased (Skovgaard et al. 2017). In pre-menopausal women, FXYD1 abundance was unchanged in *m. deltoid* after soccer, moderate intensity swim or high intensity intermittent swim training, but was increased in *m. vastus lateralis* after moderate swim training (42%) (Mohr et al. 2017). High-volume sprint interval training increased total FXYD1 (~ 90%) but did not alter phosphorylation status, with total FXYD1 remaining elevated (~ 50%) during subsequent 18 d tapering with reduced training (Skovgaard et al. 2018). Speed endurance training did not change FXYD1 abundance (Lemminger et al. 2022), whilst high-intensity training decreased FXYD5 in Type IIa but not Type I fibres, which was suggested to stabilise NKA complexes in IIa fibres (Hostrup et al. 2023). In summary, training increased FXYD1 abundance and phosphorylation in several studies. An increased FXYD1 abundance in muscle with training would dis-inhibit NKA and thus together with elevated FXYD1 phosphorylation status, would be expected to increase NKA activity in muscle, and potentially counter Na^+^/K^+^ fluxes during contractions. This would help to preserve muscle force during exercise despite elevated muscle [K^+^]_int_ and avoid fatigue that ensues under conditions of metabolic stress (Renaud et al. 2023).

Two weeks of reduced activity lowered FXYD1 protein by 18% (Thomassen et al. 2010). After complete spinal cord injury, the total FXYD1 in *m. vastus lateralis* was reduced (~ 52%), whereas phosphorylation at Serine^63^ and Serine^68^ were unchanged and FXYD5 abundance was elevated (~ 7-fold), compared to able-bodied controls (Boon et al. 2012). Time-course data after complete spinal cord section showed a reduction in total FXYD1 after 3 and 12 months (60%) but with increased phosphorylation at Ser^68^ (30%), with no changes found after incomplete spinal cord injury. Reductions after spinal injury in FXYD1 abundance, but with unchanged or increased phosphorylation and elevated FXYD5, make the overall impacts on NKA activity unclear. Lesser FXYD1 suggests less activation of NKA, but further work is required to fully understand the effects of inactivity on FXYD and NKA activity in muscle.

## Na^+^ and K^+^ ion concentrations in human skeletal muscle with exercise

In animal muscles, electrical stimulation of isolated muscles and exercise such as running or swimming induce profound reductions in [K^+^]_i_ and increases in [Na^+^]_i_ (Balog and Fitts 1996; Juel 1986; Murphy et al. 2008; Lindinger et al. 1987; Fenn 1937; Sreter 1963), as detailed elsewhere (Renaud et al. 2023). This section focusses on the effects of exercise on [K^+^] and [Na^+^] in human muscle from the late 1960s through to the 1980s.

### Measurements of [K^+^] and [Na^+^] in human skeletal muscle biopsies

The effects of exercise on [K^+^]_i_ and [Na^+^]_i_ in human muscle are shown in Table 7. Early studies in humans measured ion contents, reporting only small decreases in intracellular K^+^_c_ in *m. quadriceps femoris* after either 30 min recumbent cycling at 49 W (− 1.0 mmol·100 g glycogen-free, fat-free solids^−1^), with Na^+^_c_ increased by 0.5 mmol·100 g glycogen-free, fat-free solids^−1^) or in K^+^_c_ after cycling to exhaustion at 116 W (~ 127 min) (− 2.2 mmol·100 g glycogen-free, fat-free solids^−1^) (Bergström and Hultman 1966; Ahlborg et al. 1967). These findings were considered unimportant and inadequate to account for the subjects' exhaustion (Hultman 1967). Three studies in humans from the 1970s investigated intense cycling effects on *m. vastus lateralis* intracellular ion concentrations, measuring ion contents and extracellular water volume based on the Cl^−^ distribution, then deriving intracellular water volume and ion concentrations (Bergström et al. 1971; Costill and Saltin 1975; Sahlin et al. 1978). The mean [K^+^]_i_ at rest was 163 mM (range 150–178 mM) and fell after exercise to 146 mM (range 134–165 mM), representing a decrease of 17 mM (range 13–22 mM). Two of these studies also measured [Na^+^]_i_ which was elevated after exercise by 2.5 mM. However, the Cl^−^ distribution method did not take into account the possibility that the Cl^−^ distribution differs between rest and after exercise. It is known that the muscle cell membrane is highly permeable to Cl^−^ and that Cl^−^ influx occurs during action potentials affecting the Cl^−^ distribution, which occurs passively according to the resting E_M_ (see Renaud et al. 2023). During the 1980s, the [^3^H]-inulin distribution was used to measure changes in extracellular water content after exercise and determine intracellular [ion] (Sjøgaard and Saltin 1982; Sjøgaard et al. 1985; Saltin et al. 1981). In the first study, after three, 3 min cycling bouts at 120% *V*O_2max_, the *m. vastus lateralis* total water content and extracellular (interstitial) water content both increased, whilst the intracellular water content was unchanged (313 vs. 359, 34 vs. 60 and 280 vs. 299 ml·100 g dry weight^−1^, rest vs. exercise, respectively (Sjøgaard and Saltin 1982). These increases in muscle water with intense exercise are due to increased intracellular and extracellular osmolality, with the intracellular changes mainly due to increases in creatine, inorganic phosphate and lactate resulting from metabolic activity (Lindinger 2022). The decline in [K^+^]_i_ was from 161 to 141 mM after exercise, whilst [Na^+^]_i_ was unchanged (Sjøgaard 1983). The *E*_m_ was calculated from [K^+^]_i_ and [K^+^]_e_ (plasma [K^+^]_v_) using the equation from (Hodgkin and Horowicz 1959) and declined from − 88 to − 79 mV after exercise (Sjøgaard 1983). In a second study, [K^+^]_i_ declined from 168 to 129 mM after exhaustion and the calculated *E*_m_ declined from − 89 to − 75 mV (Sjøgaard et al. 1985). In contrast, after isometric contractions of the knee extensors at intensities up to 50% MVC, each of intracellular and extracellular water contents and [K^+^]_i_ were unchanged (Saltin et al. 1981). Muscle [K^+^]_i_ also did not differ between sexes, between muscles with varying proportions of slow and fast twitch fibres and with no difference in K^+^_c_ between slow and fast twitch muscle fibre fragments (Sjøgaard 1983). In another study, the muscular K^+^ release during incremental knee extension exercise was 3 mmol.min^−1^, whilst the total amount of K^+^ lost from the leg, which varied with both exercise intensity and duration, totaled 17 mmol during exercise at 100% *V*O_2max_ to fatigue and as much as 40 mmol for exercise lasting 2 h at 60% *V*O_2max_ (Saltin et al. 1987). The role of non-working muscle in K^+^ homeostasis during intense exercise was also explored during 4 × 30 s cycle ergometer sprint bouts, with *m. deltoid* [K^+^]_i_ unchanged from 112.7 mM at rest, despite net K^+^ uptake into the inactive forearm from arterial plasma, but [K^+^]_i_ declined to 91.8 mM at 25 min post-exercise (Lindinger et al. 1990a). These findings suggested that inactive muscle could take up K^+^ released from contracting leg muscles during exercise and that K^+^ could then be released in recovery.

**Table 7:** Historical finding﻿s (1970s–1980s) on effects of exercise on K^+^ ([K^+^]) and Na^+^ concentrations ([Na^+^]) in skeletal muscle in humans

Reference	*n*, sex (F/M)	Age	Exercise				Post-exercise muscle biopsy time (min)	[K^+^]_i_ (mmol·L^−1^)			[Na^+^]_i_ (mmol·L^−1^)		
			Mode	Type	Intensity	Dur (min)		Rest	Post	(∆)	Rest	Post	**(∆)**
Bergström et al. (1971)	3, nr	22	CE	C, S	163 W	17–20	5 20	150	134 131	− 16 − 19	9.5	11.7 16.8	+ 2.2 + 7.3
Costill and Saltin (1975)	6 M	nr	CE	C, S	80–85%*V*O_2max_								
					First run								
					1.5–2.5 h to dehydration	5	< 1	178	165	− 13	nr	nr	
					Then Second run 2–2.5 h fluid rehydration	5	< 1	196	174	− 22			
					Then Third run	5	< 1	176	162	− 14			
Sahlin et al. (1977)-Rest Sahlin et al. (1978)-CE	8 M	23–31	CE	C, HI	50%*W*_max_ + W_max_ until Exh	10–11 (total)	1 8 20	161	139 147 150	− 22 − 14 − 11	8.2	11.0 11.6 10.9	+ 2.8 + 3.4 + 2.7
Saltin et al. (1981)	8 M	nr	Iso KE	C, S C, HI C, HI	15% MVC 25% MVC, Exh 50% MVC, Exh	5–7		146	146	0	nr	nr	
						3–4							
						1–1.5		146	145				
Sjøgaard (1983)	6 M	25	CE		120%VO_2max_ (Three bouts of 3 min)	9	0.25	161	141	− 20	~ 25	23	− 2
Sjøgaard et al. (1985)	6 M	nr	KE	S HI	50–70%*V*O_2max_ then 100%*V*O_2max_ to Exh	10 6		168	150 129	− 18 − 39	6	20 24	+ 14 + 18
Kowalchuk et al. (1988b)	6 M	nr	CE	C, HI	Maximal sprint (mean P = 845W)	0.5	0.5 3.5 9.5	142	138 123 128	− 4 − 19 − 14	9.3	11.4 11.5 9.7	+ 2.1 + 2.2 + 0.4

All muscle biopsies were from *m. vastus lateralis*, except for *m. quadriceps femoris* in the Sahlin 1977, 1978 studies

Exercise details (all conducted upright): CE, cycle ergometer; KE, knee extension; Iso, isometric; C, Continuous; S, submaximal; Max, maximal (i.e. equivalent to *V*O_2max_); HI, high intensity at supramaximal workrate, i.e. exceeding *V*O_2max_. W, watt; MVC, maximum voluntary contraction; Exh ~ exhaustion; PEx, post exercise time lapse for sampling time. Dur, Duration

*nr* not reported, *n* number of participants, *F* female, *M* male, *Age* reported in mean years

In summary, studies in humans during the late 1960s through to the 1980s utilised intense dynamic exercise leading to fatigue to measure changes in muscle intracellular and extracellular water, K^+^ and Na^+^, with studies finding [K^+^]_i_ was decreased with fatigue (mean − 21 mM, range − 13 to − 39 mM), but with more variable increases in [Na^+^]_i_ (Table 7).

### Non-invasive measurements of Na^+^_c_ and K^+^_c_ in human skeletal muscles

A limiting factor in studies using muscle biopsies is the low number of sampling times, thereby preventing time-course studies for change in muscle ions. Hence, a non-invasive approach such as magnetic resonance imaging (MRI) is promising for future applications. The main advantages of using MRI include the ease of participant recruitment, minimisation of potential risks with repeated invasive procedures and time-course measurements from several muscles. However, MRI measurements are currently hampered by slow imaging times (~ 15 min) relative to the rapidity of [ion] changes in muscle during and after exercise. Furthermore, unless muscle water content is also determined from ^1^H, MRI measurements cannot differentiate between intracellular and interstitial ions, thus do not accurately reflect intracellular ion concentrations.

One early study measured the rate of ^43^K^+^ radioactive decay from the *m. quadriceps femoris* (and part of *m. sartorius*) and found that muscle K^+^_c_ declined by 3.2% during 2 h single-leg knee extension at moderate intensity at a moderate workrate (Qayyum et al. 1993). This was followed by several MRI studies measuring the signal of the naturally occurring ^23^Na isotope to determine muscle ^23^Na at rest and calculate Na^+^_c_, which was typically ~ 26 to 28 mmol·kg^−1^, but these studies used small sample sizes and poorly defined, or only mild intensity exercise protocols (Constantinides et al. 2000; Bansal et al. 2000; Weber et al. 2006). The Na^+^_c_ in calf muscles increased above rest in two males by ~ 6 mmol·kg^−1^ after 5 min of dynamic ankle plantar flexion at 40–50% MVC (Constantinides et al. 2000), but was unchanged after repeated toe lifts (Bansal et al. 2000) or after 20 min moderate cycling (Weber et al. 2006). More recently, muscle ^23^Na was measured in several muscles in 3 women and 3 men, including *m. triceps surae*, peroneal and superficial flexor muscles, medial and lateral *m. gastrocnemius* and *m. soleus*_*,*_ with Na^+^_c_ increased from a mean resting value of 34.0 (31.9–34.9) to 37.3 (35.3–38.9) mmol·kg ww^−1^ after incremental cycling (Hammon et al. 2015)*.* Recent MRI studies using a 7 T magnet have used muscle ^23^Na, ^39^K and ^1^H measurements to calculate [K^+^] and [Na^+^] (Chang et al. 2010; Gast et al. 2022a, b; Höger et al. 2022). In 7 females and 7 males, in medial and lateral *m. gastrocnemius*, *m. soleus* and *m. tibialis anterior*, [K^+^] ranged between 96 and 100 mM and [Na^+^] between 16 and 19 mM (Gast et al. 2022b), and after 5 min eccentric contractions, [K^+^] had not changed whilst [Na^+^] had increased to ~ 26 to 28 mM (Gast et al. 2022a), with Na^+^_c_ also elevated after eccentric contractions in medial *m. gastrocnemius* and *m. soleus* (Höger et al. 2022). In summary, recent MRI measurements have reported increased muscle [Na^+^] and decreased [K^+^] with exercise, especially when fatigue is involved, which are qualitatively consistent with the previous studies using muscle biopsies (Table 7).

## Human skeletal muscle interstitial [K^+^] with exercise

**Fig. 6:**
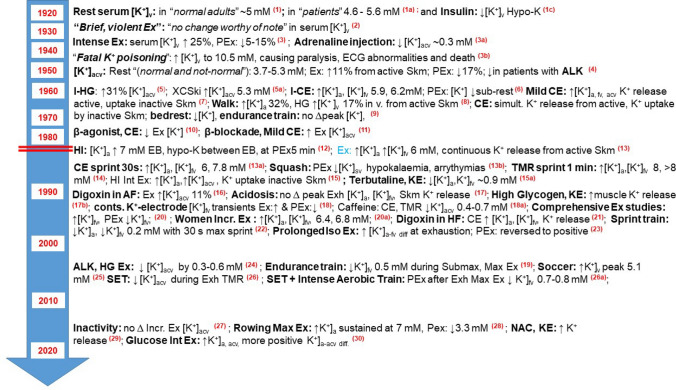
Timeline of key developments for plasma [K^+^] with exercise in humans

The resting E_m_ depends on the transmembrane K^+^ gradient which is influenced by both [K^+^]_int_ and [K^+^]_i_. The first measures of [K^+^]_int_ in contracting muscle in humans occurred almost 40 years ago, using ion-selective electrodes inserted within a needle into the *m. brachioradialis* in 3 individuals, who performed isometric handgrip contractions for 20–30 s (Vyskocil et al. 1983). Muscle [K^+^]_int_ increased from 4 to 5 mM at rest to a mean of 9.5 mM after maximal contractions and exceeded 15 mM in one individual. Although the method was challenged due to potential K^+^ leakage artefacts from damaged fibres, their [K^+^]_int_ values are similar to those obtained using the microdialysis technique that was developed 16 years later. Microdialysis measures include variability between different probes within an individual, within individuals during similar exercise and also between individuals (Juel et al. 2000; Nordsborg et al. 2003b). For example in one study, individual [K^+^]_int_ measurements ranged between 3.9 and 4.3 mM at rest, whilst during an exercise at 40 W [K^+^]_int_ ranged between 5.0 to 10.8 mM (Fig. 6 in (Juel et al. 2000)). One possible explanation for the variability is the position of the probe in relation to the activated fibres, with a smaller increase in [K^+^]_int_ when a lesser number of active fibres near the probe. Hence, we only report mean values here. The mean resting [K^+^]_int_ typically varied between 4.0 and 4.5 mM and was increased with exercise, which in some studies was proportional to exercise intensity. Thus, in *m. gastrocnemius medialis*, [K^+^]_int_ rose to 6.9, 7.4 and 7.5 mM during 15 min isometric plantarflexion contractions at 15, 30 and 45% maximum force, respectively (Green et al. 1999), whilst during one-legged knee extension exercise at 10, 30 and 50 W, the *m. vastus lateralis* mean [K^+^]_int_ increased to 6.2, 7.8 and 9.0 mM, respectively (Juel et al. 2000). Greater increases in mean [K^+^]_int_ were observed at higher intensities, reaching 11.1 mM during 5 min dynamic contractions at 85% peak power output (Green et al. 2000) and 11.9 mM in *m. vastus lateralis* during exhaustive knee extensor exercise (Nordsborg et al. 2003b). During three bouts of intense one-legged knee extensions to exhaustion, time to fatigue decreased progressively to 5.1, 4.2 and 3.2 min, whilst peak [K^+^]_int_ reached 11.4, 10.4 and 9.1 mM, respectively (Mohr et al. 2004). Thus, the point of fatigue occurred with lower [K^+^]_int_ from the first to the third bout. During 30 min of non-fatiguing knee extensor exercise at 30 W, mean [K^+^]_int_ rose during the initial 5 min to 10.2 mM, and then declined progressively to 7.5 mM, whilst in the same participants, [K^+^]_int_ reached 9.9 mM during incremental exercise to fatigue (Nielsen et al. 2004). In one leg that underwent intense intermittent training, [K^+^]_int_ was ~ 2 to 3 mM less in the trained leg throughout continuous exercise and reached 9.1 mM at fatigue during incremental exercise, similar to the untrained leg. Finally, in *m. vastus lateralis*, a [K^+^]_int_ of ~ 12 mM was found after two bouts of exhaustive cycling (each ~ 2 min), but was unchanged after intense training (~ 11 mM) (Gunnarsson et al. 2013).

Animal studies have shown that at the physiological temperature of 37 °C, [K^+^]_int_ must exceed 10–12 mM before K^+^ severely depresses tetanic force (Ammar et al. 2015; Pedersen et al. 2003; Uwera et al. 2020). Given that the [K^+^]_int_ rarely exceeds 10–12 mM in most of these microdialysis studies in humans, this suggests that the increase in [K^+^]_int_ may by itself be insufficient to cause fatigue. However, as discussed (Renaud et al. 2023), K^+^ disturbances may be a major factor in the mechanism of fatigue in combination with changes in [Na^+^]_i_ and Cl^−^ ClC-1 channel activity occurring during exhaustive exercise. Interestingly, and in contrast to the fatiguing effects of large [K^+^]_int_ elevations, the reported increases in [K^+^]_int_ are in the range that might potentiate force development during submaximal contractions (Renaud et al. 2023).

In summary, studies using microelectrodes or microdialysis demonstrated [K^+^]_int_ values increasing with exercise intensity and reaching ~ 9 to 12 mM at fatigue. However, fatigue did not always coincide with a given muscle [K^+^]_int_, and in some studies, [K^+^]_int_ reached similar levels during non-fatiguing exercise and when fatigue/exhaustion was observed. As discussed in our accompanying review, high [K^+^]_int_ in human muscles during exhaustive exercise may contribute to the mechanism of muscle fatigue, but only in combination with concomitant increases in [Na^+^]_i_ and ClC-1 Cl^−^ channel activity (Renaud et al. 2023).

## Plasma [K^+^] during and following exercise in humans

### Introduction and definitions

The following section details key chronological developments in understanding K^+^ regulation with exercise in humans during the twentieth and early twenty-first centuries, which progressed coincident with studies investigating K^+^ homeostasis in contracting animal muscles. Whilst considerable parallel research during this period included the regulation of Na^+^, Cl^−^, Lactate^−^ and H^+^ with exercise, these are considered beyond the scope of this review and apart from brief mentions of Na^+^, are not covered here. In general, earlier studies measured K^+^_c_ in blood via flame photometry or later by atomic absorption spectrophotometry, whilst many later studies utilised automated K^+^-selective electrodes, with most reporting [K^+^] in plasma. K^+^ regulation and exercise has been the focus of numerous excellent reviews that focus on implications for human integrative physiology, including muscle fatigue, heart function, blood flow and ventilation, as well as examining the roles of other tissues such as red cells and of fluid shifts per se (McKenna 1992; Lindinger and Cairns 2021; Sejersted and Sjøgaard 2000; Hostrup et al. 2021; Lindinger et al. 1995; Lindinger 2022; Renaud et al. 2023).

### Fundamental discoveries on plasma [K^+^] and exercise in the early to mid-twentieth century

#### Resting [K^+^] normative data

The first advance was in the accurate measurement of [K^+^] in serum in the early 1920s, which allowed determination of resting values of 4.6–5.6 mM in healthy individuals and patients with varying pathologic conditions (Kramer and Tisdall 1921; Wilkins and Kramer 1923). Later determinations of venous plasma [K^+^] at rest in 70 healthy individuals ranged from 3.7 to 5.3 mM (Farber et al. 1951), similar to the normal range of 3.5–5.5 mM commonly used clinically today.

#### Foundational studies on exercise and plasma [K^+^] during the 1930s through 1960s

The first study to investigate exercise effects on [K^+^] reported “no [other] change worthy of note” in venous serum [K^+^] after treadmill running (Dill et al. 1930). Later, it was reported that “brief violent” exercise leading to exhaustion in 1 min led to a 25% increase in venous serum [K^+^] “immediately at the end of work”, which in recovery then “drops precipitously” to 5–15% below rest after 10–15 min, followed by up to a 20% increase after 40 min and a return to rest levels after 1–1.5 h recovery (Keys 1937). From the middle of the twentieth century, usage of flame photometry and cannulation facilitated analysis of plasma [K^+^] in repeated samples and more comprehensive exercise studies in humans, with these foundational studies detailed in Table 8.

**Table 8:** Historical findings (1930–1968) on plasma [K^+^] at rest, during and after exercise in healthy humans

References	*n*/sex	Age	Exercise				Sample time (min)	Plasma [K+] (mM)			
			Mode	Type	Intensity	Dur (min)	Rest/Ex/PEx	[K^+^]_a_	Vein	Venous [K^+^]	a–v difference
Dill et al. (1930)	9 M	30	TMR	C, S	9.3 km.h^−1^, *V*O_2_ ~ 2 L min^−1^	20	Rest, PEx: + 1		acv	Serum 3.3, 3.2	
Keys (1937)^a^	15 M	nr	nr	C, HI	“Violent”, Exh	~ 1	PEx: immed., 10-15		acv	serum ↑25%, ↓15% (mM data nr)	
Farber et al. (1951)	12 nr 12 nr 6 nr	nr	HGRest	Int, S	Open/close fist 10 times	nr	Rest, Ex: “end” PEx: + 2		sv sv sv	4.4 5.1 4.4	
	2 nr 2 nr 2 nr		KE	Int, S	KE every 2 s	2	Rest, Ex: “end” PEx: + 2	No change (nr) No change (nr)	fv fv fv	4.0 4.7 4.2	
Skinner (1961)	6 M	nr	HG	Int, S	“Intense” contractions		Rest Ex: “during” PEx: + 0.25–0.5		acv, sv acv, sv acv, sv	4.1, 4.2 5.4, 4.6 5.3, 5.0	
	10 nr		HG	Int, S	“Slight” contractions		“Rest”		sv	0.1–0.8 greater than controls	
Thiebault et al. (1963)	40 M	nr	CE	C, S	200 W	10	Rest, PEx: “immed.”		acv acv	4.7 4.7	
Kilburn (1966)	7 M	22–33	TMW	C, S	4–5.6 km.h^−1^, *V*O_2_ ~ 2 L min^−1^	6	Rest Ex: 5	3.8 5.0			
	9 M		HG	Int, S	60 contractions	6	Rest Ex: final min	4.2 4.2	acv	4.1 4.8	
Laurell and Pernow (1966)	6 M	nr	CE	Incr-Max	Up to 245–294 W	nr	Rest Ex: “during” PEx: 0.5, 5, 20	4.5 5.9 5.4, 4.3, 4.8	fv fv fv	4.6 6.2 5.2, 3.9, 4.9	− 0.08 − 0.24 + 0.21, + 0.42, − 0.06
	3 M	nr	HE	nr	nr	nr	Rest Ex: “during” PEx: 0.5, 5, 20	4.7 5.1 5.1, 4.8, 4.9	acv acv acv	4.7 5.7 5.1, 4.8, 4.9	− 0.03 − 0.62 + 0.23, + 0.45, + 0.12
Bergström and Hultman (1966)	1 nr	nr	CE_sup_	C, S	49 W	30	Rest Ex: 2, 10, 22 PEx: + 7, + 35	3.5 3.9, 3.9, 3.8 3.6, 3.4	fv fv fv	3.7 4.3, 4.2, 4.0 3.5, 3.5	− 0.13 − 0.43, − 0.28, − 0.20
											+ 0.07, − 0.06
							Rest Ex: 2, 10, 22 PEx: + 7, + 35		acv acv acv	3.8 3.8, 3.9, 3.9 3.7, 3.6	− 0.23 + 0.07, + 0.07,− 0.08 − 0.1, − 0.13
Saltin et al. (1968)	3–5 M	20	CE	C, Incr-Max			Rest Ex: "during" Ex, Ex, Ex	4.3 4.6 4.3, 4.6, 4.8	fv fv fv	4.2 4.7 4.4, 4.6, 4.8	− 0.1 − 0.1 − 0.1, − 0.1, − 0.1
			CE_sup_		98 W 40, 60, 80% *V*O_2max_						
			TMR^b^		Max		Ex	5.5	fv	5.4	− 0.05
							Rest Ex Ex, Ex, Ex Ex		acv acv acv acv	4.2 4.3 4.0, 3.9, 4.5 4.5	+ 0.1 + 0.3 + 0.3, + 0.7, + 0.5 + 0.9
	1 M		TMR	C	80% *V*O_2max_	~ 31	Ex 6–7	5.6		6.1	− 0.5

Blank cell indicates that variable was either not measured (e.g. a blood sampling site) or value not reported; methods details often limited by inadequate description. Details on experiments conducted on patients were excluded

*(nr)* if not reported, *n* number of participants, *sex F*, female, *M* male (n F/n M); age is reported mean years, *m* muscle, *a* arterial, *fv* femoral venous, *acv* antecubital venous. *s.v.*, superficial venous

Exercise details and abbreviations: Mode: CE, cycle ergometer, TMR, treadmill running; TMW, treadmill walking; S, cross country skiing; KE, knee extension; HG, handgrip; HE, hand ergometer. All exercise conducted upright, unless indicated by subscript sup, supine, or s-r, semi-recumbent

Type: C, Continuous; Incr. incremental; Int, Intermittent; exercise intensity classified broadly as S, submaximal; Max, maximal (i.e. equivalent to *V*O_2max_); HI, high intensity at supramaximal workrate, i.e. exceeding *V*O_2max_

Intensity: either as % *V*O_2max_, *V*O_2_ (L.min^−1^), workrate in watts (W) or running speed (km h^−1^); Dur: exercise duration in minutes; Exh ~ exhaustion

Sample times: blood sampling times are Rest, during (Ex) and post-exercise (PEx). Where exercise sampling time was not specified, this is denoted as Ex, and where was not clearly specified as being sampled during exercise, these are indicated as PEx

Plasma [K^+^] and abbreviations: Resting [K^+^] not included if not reported. All measures are in plasma unless indicated as serum. Values rounded to one decimal place, except a–v differences at two decimal places when reported as such

[K^+^]_a_, arterial [K^+^]; [K^+^]_fv_, femoral venous [K^+^]; [K^+^]_acv_, antecubital venous [K^+^]; [K^+^]_sv_, superficial venous [K^+^]; the a–v differences as reported or calculated from the arterial and venous [K^+^]

^a^Method details from (Keys and Adelson 1936); exercise mode nr, but might be any of TMR, field/track running and/or rowing

^b^Methods unclear whether cycling or treadmill used for submaximal and maximal exercise K^+^

Arterio-venous plasma [K^+^] differences ([K^+^]_a-v diff_) were also measured across the forearm and leg to demonstrate the direct importance of the contracting musculature on [K^+^], finding plasma [K^+^] was elevated in venous blood draining the active forearm or leg, but not in arterial plasma [K^+^] ([K^+^]_a_) (Farber et al. 1951). Furthermore, during handgrip exercise, venous [K^+^] ([K^+^]_v_) was not increased in blood draining non-active muscle. Clinicians were concerned whether “fist pumping” during venous phlebotomy might artificially elevate [K^+^]_v_ and studies revealed that mild and more intense rhythmic forearm muscle contractions elevated superficial forearm venous plasma [K^+^] ([K^+^]_sv_), antecubital venous plasma [K^+^] ([K^+^]_acv_) and with a widening of the arterial-antecubital venous [K^+^] difference ([K^+^]_a-acv_) (Skinner 1961; Hultman and Bergström 1962). Four detailed exercise studies in the 1960s confirmed that the contracting muscles were the origin of elevations in [K^+^] (Kilburn 1966; Laurell and Pernow 1966; Bergström and Hultman 1966; Saltin et al. 1968). During treadmill walking [K^+^]_a_ rose 1.2 mM above rest to 5.0 mM, handgrip exercise had no effect on [K^+^]_a_ but increased [K^+^]_acv_ by 0.7 mM above rest to 4.8 mM (Kilburn 1966), whilst hand ergometer exercise increased [K^+^]_acv_ to 5.7 mM, with a wide [K^+^]_a-acv diff_ of − 0.62 mM (Laurell and Pernow 1966). The first study to use intense incremental cycling exercise reported pronounced increases in both [K^+^]_a_ and femoral venous [K^+^] ([K^+^]_fv_) to 5.9 and 6.2 mM, respectively, with a negative arterio-femoral venous plasma [K^+^] difference ([K^+^]_a-fv diff_), indicating that the contracting leg musculature was the source of the K^+^ (Laurell and Pernow 1966). In addition, [K^+^]_a_ and [K^+^]_fv_, respectively declined by ~ 0.5 and ~ 1 mM at 30 s after exercise to fall below rest values, with a positive corresponding [K^+^]_a-fv diff_, indicating K^+^ reuptake by the leg muscles after exercise. The first study to simultaneously measure [K^+^]_a_, [K^+^]_fv_ and [K^+^]_acv_ during exercise found each was elevated during mild recumbent cycling, with a negative [K^+^]_a-fv diff_ confirming K^+^ release from the exercising leg, that reversed to K^+^ uptake immediately after exercise; in contrast, a positive [K^+^]_a-acv diff_ during exercise indicated K^+^ uptake by the arm, which reversed to K^+^ release from the arm in recovery (Bergström and Hultman 1966). Simultaneous K^+^ release from the exercising limb and K^+^ uptake by an inactive limb was confirmed by findings of a small negative [K^+^]_a-fv diff_ and positive [K^+^]_a-acv diff_ during most submaximal exercise workrates, although findings for the active leg were less clear during maximal exercise (Saltin et al. 1968). Thus, by the end of the 1960s, it was established that intense exercise in humans induces marked perturbations in circulating [K^+^], comprising substantial elevations during exercise followed by a rapid decline post-exercise, in some instances to sub-resting concentrations. It was further established that contracting muscles released K^+^ into the plasma (i.e. negative [K^+^]_a-v diff_) which reversed to K^+^ uptake during recovery (i.e. positive [K^+^]_a-v diff_) and that non-contracting muscles removed K^+^ from the circulation during exercise (i.e. positive [K^+^]_a-v diff_).

#### Elevated [K^+^], paralysis and death: new understanding during the 1940s

Clinicians also began to understand the critical impacts of high systemic [K^+^] during this period, with the first studies that directly linked high [K^+^] with paralysis and death in humans published during and shortly after the Second World War. These studies observed high [K^+^] (> 8 mM), neuromuscular paralysing effects and death due to cardiac arrest after crush injuries and uremia (Finch and Marchand 1943; Marchand and Finch 1944; Finch et al. 1946). In crush victims from bombing raids in London, serum [K^+^] rose above 10 mM, K^+^_c_ in crushed muscle fell by two-thirds and urinary K^+^ was high, with death common within the first week after injury (Bywaters 1944). Insulin and dextrose could lower [K^+^] and reduce ECG abnormalities such as heightened T waves (Bywaters 1944). Thus, basic knowledge on the effects of high [K^+^] on paralysis and death were established by the middle of last century, when induced by renal disease, injury or treatment. Whilst these clinical studies did not involve exercise, they are relevant in understanding the safe upper limits of [K^+^]. At that time, there remained, however, a lack of awareness of the extent of exercise hyperkalaemia and post-exercise hypokalaemia.

### Detailed knowledge on plasma [K^+^] and exercise: studies during 1975–1999

**Fig. 7:**
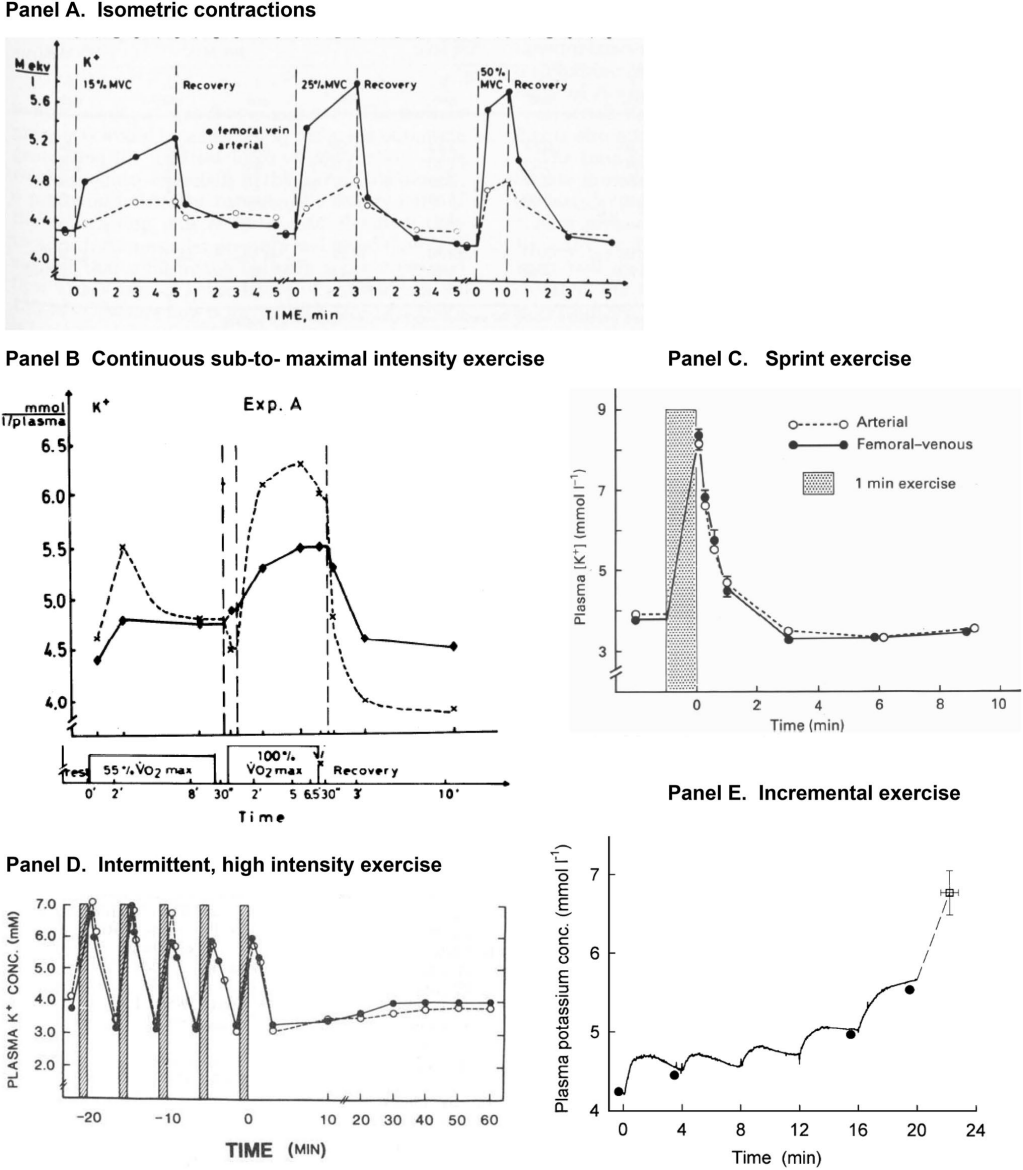
Arterial and femoral venous plasma [K^+^] during and after different types of exercise. **A** Isometric exercise. Arterial (o- - o) and femoral venous (•−•) plasma [K^+^] before, during and after knee extensor muscle contractions at 15%, 25% and 50% maximal voluntary contractions (MVC), for 5, 3 and 0.5 min, respectively, each followed by 5 min rest (*n* = 4–8, males). From (Saltin et al. 1981). **B** Continuous submaximal-to-maximal intensity exercise. Arterial (◆−◆) and femoral venous (X- - X) plasma [K^+^] before, during and after knee extension exercise for 10 min at 55% followed by 0.5 min rest and then to exhaustion at 100% *V*O_2max_ lasting ~ 7 min and 10 min recovery (*n* = 3, males). From (Sjøgaard et al. 1985). **C** Sprint exercise-continuous, short duration, high intensity. Arterial (o- - o) and femoral venous (•−•) plasma [K^+^] before, during and after 1 min exhaustive treadmill running, followed by 9 min recovery (*n* = 12, males, mean ± standard error of the mean). From (Medbø and Sejersted 1990). The “exercise” sample was taken about 10 s after completion of exercise bout and at 0.3, 1, 3, 6 and 9–10 min recovery. **D** Intermittent exercise. Arterial plasma [K^+^] before, during four, 1 min cycling bouts at 100% *V*O_2max_, separated by 1 min rest, then 60 min recovery in endurance (o- - o) and sprint trained (•−•) (*n* = 4 each group, sex not specified). Blood was sampled immediately after exercise bouts as well as in the rest period 30 s before the next bout and in recovery at 1, 2, 5, 10, 20, 30, 40, 50 and 60 min recovery. From (Hermansen et al. 1984). **E** Incremental exercise. Peak arterial [K^+^] (•) and femoral venous (--- - -) plasma [K^+^] before, during incremental cycling, with work rate every 4 min until exhaustion. Data from (Hallén et al. 1994) redrawn in (Hallén 1996). Continuous femoral venous [K^+^] data collected from a K^+^-electrode inserted into the vein

The final quarter of the twentieth century saw an upsurge in mechanistic studies investigating plasma K^+^ regulation with exercise in humans. This section focusses on plasma [K^+^] and exercise, with studies described after first classifying by exercise type, as either isometric, continuous submaximal, continuous high intensity, intermittent or incremental exercise, as detailed in Table 9. A timeline of the early and later developments in understanding plasma [K^+^] with exercise is shown in Fig. 6 and examples of arterial and femoral venous plasma [K^+^] during different types of exercise in Fig. 7.

**Table 9:** Plasma [K+] during and after exercise in healthy humans, from key studies during the last quarter of the twentieth century

References	*n*/sex	Age	Exercise details			Blood sampling time (min)	Plasma [K^+^] (mM)			
			Mode	Description	Dur (min)		[K^+^]_a_	Vein	[K^+^]_v_	a–v difference
*(9A) Isometric exercise*										
Saltin et al. (1981)	8 M	nr	KE	Rest			4.3	fv	4.3	0.0
				10–15%, 25%, 50% MVC	5, 3, 1	5, 3, 1	4.6, 4.6, 4.8		5.2, 5.8, 5.7	− 0.6, − 1.2, − 0.9
				Post-exercise		3, 5	4.4, 4.3		4.4, 4.4	+ 0.0, + 0.1
Sjøgaard (1988)	6 M	27	KE	Rest			4.3	fv	4.3	0.0
				5%, 15%, 25%, 50% MVC	30, 5, 3 1	30, 5, 3, 1	4.5, 4.3, 4.8, 4.8		4.7, 4.8, 5.7, 5.7	− 0.2, − 0.5, − 0.9, − 0.9
				Post-exercise		2	4.3		4.3	0.0
Fallentin et al. (1992)	7 M	28–43	HG	Rest				acv	3.9	
				15% MVC	3	1, 3			4.8, 5.0	
				30% MVC	3	1, 3			5.1, 5.8	
				Post-exercise					3.7	
Hallén and Sejersted (1993)	1	nr	KE	MVC	0.17	0.17		fv	↑ 0.2	
				Post-exercise		0.23			↑ 1.2	
				Post-exercise		1.0			0.2 below pre-Ex	
	1	nr	KE	MVC	1	0.2, 0.5, 1.0		fv	↑ 0.2, ↑ 1.0, ↑ 2.0	
				Post-exercise		1.0			0.5 above pre-Ex	
	1	nr	KE Int	45% MVC 36X 6:4 s W:R)	6	0.17, 0.5, 6.0		fv	↑ 0.4, ↑ 1.5, ↑ 1.9	
				Post-exercise		1.0			↑ 0.4 above pre-Ex	
West et al. (1996)	10 M	22	KE	Rest			4.1	fv	4.0	+ 0.1
				30% MVC	3	3 (+ 5 s)	5.1		5.9	− 0.8
				Post-exercise		5	3.8		3.7	+ 0.1
Verburg et al. (1999)	7 M 2F	26	2 leg KE	Rest			4.1	fv	4.0	+ 0.1
				30% MVC (6:4 s W:R)	60	1, 29, Exh	4.3, 4.7, 4.8		5.9, 4.8, 5.1	− 0.6, − 0.1, − 0.2
				Post-exercise		1, 20	4.5, 3.9		4.2, 3.9	+ 0.3, 0.00
*(9B) Continuous submaximal up to maximal intensity exercise*										
Linton et al. (1984)	3 M		CE	Rest			3.8			
				100 W	5–7	2, 5	5.4, 5.4			
Sjøgaard et al. (1985)	A) 3 M	nr	KE	Rest			4.4	fv	4.6	− 0.2
				A) 50–70% VO_2max_	8	2, 8	4.8, 4.7		5.5, 4.8	− 0.7, − 0.1
	B) 3 M			B) 50–70% VO_2max_	20	3, 17	4.9, 5.0		5.3, 5.2	− 0.4, − 0.2
Sahlin and Broberg (1989)	8 M	31	CE	Rest			4.0	fv	4.0	− 0.03
				67% VO_2max_	65	20, 40	5.1, 5.1		5.2, 5.3	− 0.09, − 0.15
				Exh		60–65	5.4		5.6	− 0.19
Rolett et al. (1990)	12 M	25	KE	Rest			4.1	fv	4.1	0.0
				67% (38 W)	20	5, 20	4.4, 4.4		4.6, 4.6	− 0.2, − 0.1
Lindinger et al. (1994)	4 M	23	CE	Rest			4.6	fv	4.3	+ 0.3
				75% VO_2max_	50	0.5, 2, 30	5.0, 5.6, 5.4		5.6, 5.7, 5.4	− 0.6, − 0.2, 0.0
				Exh		50	5.5		5.6	− 0.1
*(9C) Single short continuous exercise bout at high intensity*										
Sejersted et al. (1982)	1 M, ST	33	TMR	Rest			4.0			
				Exercise to Exh	1	1 (+ Immed. after)	6.5			
				Post-exercise		3, 6	3.6, 3.5			
	1 M, ET	26	TMR	Rest			3.6			
				EB to Exh	1	1	6.1			
				Post-exercise		3, 6	3.2, 3.1			
Sjøgaard et al. (1985)	3 M	nr	KE	(A) Rest			4.4	fv	4.6	− 0.2
				100% VO_2max_ to Exh	6–8	5	5.5		6.3	− 0.8
				Post-exercise		3	4.6		3.9	+ 0.7
	3 M	nr	KE	(B) Rest			4.5	fv	4.4	+ 0.1
				100% VO_2max_	5–7	6	5.5		6.0	− 0.5
				Post-exercise		30	4.5		4.5	0.0
Medbø and Sejersted (1985)	6 ST	25	TMR	Rest			4.0			
				Exercise to Exh	1	1 (+ 10–15 s)	6.6			
				Post-exercise		6, 60	3.5, 4.2			
	6 ET	25	TMR	Rest			3.8			
				EB to Exh	1	1	6.8			
				Post-exercise		6, 60	3.4, 3.8			
Kowalchuk et al. (1988b)	3 M	~ 25	CE	Max sprint (mean power 700W)						
				Rest			4.5	fv	5.4	− 0.9
				Exercise to Exh	0.5	0.5 (+ immed. after)	6.9		7.8	− 0.9
				Post-exercise		0.5, 1, 1.5,2.5	6.3,5.6,5.2,4.8		6.9, 6.1, 5.7, 5.3	− 0.6, − 0.5, − 0.5, − 0.5
Kowalchuk et al. (1988a)	6 M	30	CE	EB Max Sprint to Exh (mean power 845 W)						
				Rest			4.3	acv	4.3	0.0
				Unexercised arm	0.5	0.5 (+ immed. after)	7.2		5.9	+ 1.3
				Post-exercise		0.5, 1, 1.5,2.5	6.3,5.6,5.1,4.5		5.4, 5.1, 4.9, 4.5	+ 0.9, + 0.5, + 0.2, 0.0
Paterson et al. (1989)	6 M	21	CE	Rest			3.6			
				100 W	6	1, 2, End	4.2, 4.5, 4.6			
				Post-exercise		1, 3	4.1, 3.9			
				Rest			4.0			
				Sprint to Exh	1.7	End	7.0			
				Post-exercise		1, 3	3.9, 3.8			
Medbø and Sejersted (1990)	12 M (ST ET)	~ 25	TMR	(A) Rest			3.9	fv	3.8	+ 0.1
				Max speed to Exh	1	1 (+ 10 s)	8.2		8.3	− 0.1
				Post-exercise		1, 3, 6	4.7, 3.5, 3.3		4.4, 3.2, 3.3	+ 0.3, + 0.3, + 0.0
	6-8 M (ST ET)	~ 25	TMR	(B) 40% max speed 70% max speed	1 1	Rest, 1 (+ 10 s), PEx_nadir_		fv	4.1, 5.5, 3.7 3.8, 6.4, 3.5	
				92% max speed 100% max speed 100% max speed	1 0.4 0.7		nr, 7.5, nr		3.8, 7.7, 3.2 3.7, 6.5, 3.3 3.5, 7.4, 3.2	− 0.2
Juel et al. (1990)	10 M	23–29	KE_sup_	Rest			4.2	fv	4.1	+ 0.1
				65 W to Exh	3.18	0.5,1.5,3	4.5, 5.1, 5.8		5.7, 6.6, 6.8	− 1.2, − 1.5, − 1.0
				Post-exercise		1.5, 6, 8	4.0, 3.8, 4.0		3.6, 3.2, 3.8	+ 0.4, + 0.6, + 0.2
Hallén and Sejersted (1993)	1	nr	KE	(A) Rest			3.6	fv	3.7	− 0.1
				95% power (70W)	8	1,10	4.0, 4.6		4.3, 4.7	− 0.3, − 0.1
				Post-exercise		2	3.7		3.5	+ 0.2
	1	nr	CE	(B) Rest				fv	4.0	
				85% VO_2max_	6.5	6			5.5	
				Post-exercise		1.5			4.0	
*(9D) Intense intermittent exercise*										
Costill and Saltin (1975)	6 M	nr	CE	80–85% *V*O_2max_	3 × 5	EB1–EB3		acv	~ 4.4	
Hermansen et al. (1984)	4 ST		TMR	Rest			3.9			
				5xEB at a speed causing Exh in 60 s for the 2^nd^ bout, with 4–4.5 min rest periods	0.6–1	EB1 (+ 10 s), PEx_4 min_	6.8, 3.1			
						EB2 (+ 10 s), PEx_4min_	7.0, 3.1			
						EB3 (+ 10 s), PEx_4min_	5.9, 3.0			
						EB4 (+ 10 s), PEx_4min_	5.9, 3.1			
						EB5 (+ 10 s), PEx_4min_	6.0, 3.1			
				Post-exercise		10, 30	3.3, 3.8			
	4 ET		TMR	Rest			4.1			
				5xEB at a speed causing Exh in 60 s for the 2nd bout, with 4–4.5 min rest periods	0.6–1	EB1 (+ 10 s), PEx_4min_ EB2 (+ 10 s), PEx_4min_ EB3 (+ 10 s), PEx_4min_ EB4 (+ 10 s), PEx_4min_ EB5 (+ 10 s), PEx_4min_	7.2, 3.3 6.9, 3.2 6.8, 3.1 5.7, 3.0 5.7, 3.0			
				Post-exercise		10, 30	3.3, 3.6			
Katz et al. (1985)	4F, 4 M	25	CE	Rest			3.7	fv	3.8 5.7, 4.4 5.2, 4.2	− 0.1 − 0.6, + 0.1 − 0.4, + 0.2
				4xEB at 100% *V*O_2max_, with 1 min rest periods	1	EB2, PEx_1min_ EB4, PEx_5min_	5.1, 4.5 4.8, 4.4			
				Post-exercise		10, 30	3.5, 3.7		3.4, 3.7	+ 0.1, + 0.1
McKelvie et al. (1989)	5 M	30	CE	Rest			4.7			
				4xEB at max effort Mean power EB1-EB4: 800, 700, 600, 533 W, with 4 min rest periods	0.5	EB1 (+ 15 s) EB2 (+ 15 s) EB3 (+ 15 s) EB4 (+ 15 s)	6.6 6.4 5.9 5.9			
				Post-exercise		5, 15, 90	4.3, 4.4, 4.2			
Lindinger et al. (1990a)	8 M	22–44	CE	Rest			4.3	acv	4.5 5.5 5.2 5.2 4.8	− 0.2 + 0.6 + 0.6 + 0.4 + 0.6
				Leg exercise/resting arm muscle 4xEB at max effort Mean power EB1-EB4: 803, 707, 611, 562 W, with 4 min rest periods	0.5	EB1 (+ 15 s) EB2 (+ 15 s) EB3 (+ 15 s) EB4 (+ 15 s)	6.1 5.8 5.6 5.4			
				Post-exercise		5, 15, 90	3.8, 3.9, 3.8		3.8, 4.0, 4.2	0.0, − 0.1, − 0.4
Medbø and Sejersted (1990)	4 M ST	~ 25	TMR	Rest			3.8			
				5xEB at speed causing Exh in 60 s for 2nd bout, with 4 min rest periods	1	EB1 (+ 10 s), PEx_4min_ EB2 (+ 10 s), PEx_4min_ EB3 (+ 10 s), PEx_4min_ EB4 (+ 10 s), PEx_4min_ EB5(+ 10 s), PEx_4min_	7.2, 3.3 7.4, 3.3 6.2, 3.4 6.2, 3.4 6.3, 3.5			
				Post-exercise		10, 30, 60	3.5, 4.0, 4.0			
	4 M ET	~ 25	TMR	Rest			4.0			
				5xEB at speed causing Exh in 60 s for 2nd bout, with 4 min rest periods	1	EB1 (+ 10 s), PEx_4min_ EB2 (+ 10 s), PEx_4min_ EB3 (+ 10 s), PEx_4min_ EB4 (+ 10 s), PEx_4min_ EB5 (+ 10 s), PEx_4min_	7.9, 3.4 7.6, 3.4 7.6, 3.3 7.0, 3.3 6.5, 3.4			
				Post-exercise		10, 30, 60	3.5, 3.7, 3.9			
Lindinger et al. (1992)	5 M	24	CE	Rest			4.7	fv	4.8	− 0.1
				4 EB at max speed Mean power EB1-EB4: 800, 680, 552, 504 W, with 4 min rest periods	0.5	EB1 (+ 15 s)	6.5	fv	6.1	+ 0.4
						EB2 (+ 15 s)	6.2	fv	6.2	0.0
						EB3 (+ 15 s)	5.7	fv	5.7	0.0
						EB4 (+ 15 s)	5.8	fv	5.4	+ 0.4
				Post-exercise		5, 15, 90	4.2, 4.3, 4.1		3.9, 4.1, 4.2	+ 0.3, + 0.2, − 0.1
Bangsbo et al. (1992a)	6 M	22–26	KE_sup_	Exh EB1 130%*V*O_2peak_ (61W); 7 × 15 s Ex/Rest; Exh EB2 (63W)	3.73, 2.98	EB1, EB2	5.6, 5.3	fv	6.2, 5.9	
*(9E) Incremental exercise and different exercise modalities*										
Greenleaf et al. (1979)	4 M	26–45	CE CE_sup_			PEx: 0.5,5		acv	nc small ↑, ↓	
Wilkerson et al. (1982)	5 M	29	TMR	Rest				acv	4.4	
				30% *V*O_2max_ 45% *V*O_2max_ 60% *V*O_2max_ 75% *V*O_2max_ 90% *V*O_2max_	20	9, 19			4.6, 4.6 5.0, 4.9 5.0, 5.0 5.0, 5.3 5.5, 6.0	
Pivarnik et al. (1988)	10 M	26	CE (50 rpm)	Rest				acv	4.1	
				20% *V*O_2max_ 30% *V*O_2max_ 40% *V*O_2max_ 50% *V*O_2max_ 60% *V*O_2max_ 70% *V*O_2max_	5	5			4.3 4.3 4.5 4.6 4.7 5.0	
Paterson et al. (1990)	6 M	19	CE	Rest			3.8			
				50 W 100 W 150 W 200 W 250 W *W*_Exh_ (Varied between participants)	9–14 (varied between participants)	2 4 6 8 10 9–14	4.1 4.4 4.7 5.1 5.5 6.4			
				Post-exercise		8	3.8			
Vøllestad et al. (1994)	3-4 M	28	CE	Rest				fv	4.4	0.0
				60% *V*O_2max_ 85% *V*O_2max_ 110% *V*O_2max_ 140% *V*O_2max_	10 10 2.5_Exh_ 1.5_Exh_	Peak_1.5_, End, Post_1_ Peak_1.5_, End, Post_1_ Peak_2.5_, Post_1_ Peak_2_, Post_1_			6.0, 5.2,3.6 6.4, 5.9, 3.5 8.2, 3.4 8.0, 3.2^a^	
	6 M	28	CE	Rest			3.8	fv	3.8	0.0
				60%	20	Peak_2min_, End_20min_	5.7, 5.2		6.0, 5.3	− 0.3, − 0.1
				85%	10	Peak_2min_, End_10min_	6.0, 5.8		6.4, 6.0	− 0.4, − 0.2
				110%	3.8_Exh_	1, 3.8_Peak_	5.0, 8.0		5.6, 8.2	− 0.4, − 0.2
						Post 1, 6	6.1, 3.7		5.4, 3.8	+ 0.7, − 0.1
Hallén et al. (1994)	6F	21	CE	Rest	22.2		4.2	fv	4.3	− 0.01
				Start 30–40 W, increment 30–40 W every 4 min until Exh		3.5, 15.5, 19.5	4.5, 5.0, 5.5		4.5, 5.0, 5.6	− 0.1, − 0.1, − 0.1
						22.2_Exh_	6.4		6.8	− 0.3
				Post-exercise		4.0	3.8		3.7	0.1
Juel et al. (1999)	7 M	24–27	2 leg KE	Rest			4.0		4.1	− 0.1
			KE + AE	72 W (total)	10	10	4.4	fv	4.5	− 0.1
				72 incremented to 300 W (total)	10	19	5.6		5.2	+ 0.4
			2 leg KE	72 W (total)	10	30	4.4		4.5	− 0.1

Plasma [K^+^] values are as reported in text or interpolated from figures; if not reported the arterial–venous (a–v) differences were calculated. If data reported in text differed from that in Figures/Tables the latter time-series data was used. Sampling times indicate when blood was sampled during exercise or how much time after exercise for post-exercise sampling. All [K^+^] were rounded to one decimal place

*[K*^*+*^*]*_*a*_ arterial, *[K*^*+*^*]*_*fv*_ femoral venous, *[K*^*+*^*]*_*acv*_ antecubital venous, *M* male, *F* female, *W* watt, *AE* arm exercise, *CE* cycle ergometer, *KE* knee extension, *HG* hand grip, *MVC* maximum voluntary contraction, *TMR* treadmill running, *ST* sprint trained, *ET* endurance trained, *Ex* exercise, *Exh* exhaustion, *EB* exercise bout, *Post* post-exercise, *nr* not reported

^a^Data from only one subject, mean nr

#### Isometric exercise

Knee extensor and handgrip isometric contractions (Table 9A) both increased [K^+^]_a_ and to a greater extent also the corresponding [K^+^]_v_, with a negative [K^+^]_a-v diff_ during contractions indicating net K^+^ release, which reversed post-exercise to positive values, or a net K^+^ uptake (Saltin et al. 1981; Fallentin et al. 1992; Sjøgaard 1988; Hallén and Sejersted 1993; West et al. 1996; Unsworth et al. 1998; Verburg et al. 1999). Higher contraction intensities were generally accompanied by greater [K^+^] and a wider [K^+^]_a-v diff_ during contractions, e.g., during quadriceps contractions at 5–15% versus 50% MVC, respectively, the rise above rest for [K^+^]_a_ was ~ 0–0.3 versus ~ 0.5 mM, in [K^+^]_fv_ was ~ 0.5 to 0.9 versus ~ 1.4 mM, with the [K^+^]_a-v diff_ from ~ − 0.6 versus − 0.9 mM (Saltin et al. 1981; Sjøgaard 1988) (Fig. 7A). These differences were likely due to higher stimulation frequencies and cellular K^+^ efflux at higher intensities. However, intensity and [K^+^] were not directly related, with no or lesser further increases in [K^+^] above 25–50% MVC, due to elevated intramuscular pressure at higher intensities occluding blood vessels and reducing venous outflow. This occlusion effect was demonstrated when [K^+^]_fv_ was continuously monitored with a K^+^-sensitive electrode, where [K^+^]_fv_ rose by only 0.2 mM during a short, maximal isometric contraction but then immediately afterwards rose abruptly by 1.2 mM (Hallén and Sejersted 1993). Finally, [K^+^]_fv_ increased substantially, by ~ 1.9 mM during repeated 6 s intermittent contractions at 35–45% MVC (Hallén and Sejersted 1993; Verburg et al. 1999). Elevated [K^+^] was proposed to play a role in regulating blood pressure responses to isometric contractions (Saltin et al. 1981; Fallentin et al. 1992), likely mediated via increased [K^+^]_int_ stimulating Group III and IV afferents in muscle (Rybicki et al. 1984; McCloskey and Mitchell 1972), although numerous other intramuscular factors also activate Group III and IV afferents which, nonetheless, have been demonstrated to be important in the exercise pressor response (Mitchell et al. 1983; Mitchell 1990; Rowell and O'Leary 1990; Amann et al. 2010). Findings of elevated [K^+^]_fv_ after isometric contractions also led to proposal that elevated [K^+^]_int_ in fatigue may link metabolic insufficiency with impaired NKA function (Fallentin et al. 1992) and further studies explored a possible link between [K^+^] and fatigue (West et al. 1996; Unsworth et al. 1998; Verburg et al. 1999). After an isometric contraction of the knee extensors, the post-exercise [K^+^]_fv_ was related to muscle twitch force, but not to M-wave characteristics, which were potentiated, which suggested a role of elevated [K^+^]_int_ in muscle fatigue, not via impaired sarcolemmal excitability, but suggesting a t-tubule membranes locus (West et al. 1996; Unsworth et al. 1998). However, M-waves reflect primarily sarcolemmal and not t-tubular activation and interpretation is far more complex than earlier thought (Rodriguez-Falces and Place 2021). The possible role of elevated extracellular [K^+^] in fatigue is discussed in detail in our companion review (Renaud et al. 2023).

#### Continuous submaximal up to maximal intensity exercise

Knee extension or cycling exercise conducted between 50 and 75% *V*O_2max_ (Table 9B) induced a moderate increase in [K^+^]_a_ from 4.3 mM at rest to 4.8 mM (mean values) during the initial period of exercise and in [K^+^]_fv_ from 4.3 to 5.2 mM (Sjøgaard et al. 1985; Sahlin and Broberg 1989; Rolett et al. 1990; Lindinger et al. 1994). Most studies reported little variation in [K^+^]_a_ and [K^+^]_fv_ for the duration of the exercise, except at exhaustion, where two studies reported that [K^+^]_a_ and [K^+^]_fv_ increased to 5.5 and 5.6 mM. In each case, release of K^+^ from contracting muscles was indicated by a negative [K^+^]_a-fv diff_, although in one study, the calculated K^+^ flux disappeared after accounting for fluid movement from plasma into muscle (Lindinger et al. 1994).

#### A single short continuous exercise bout at high intensity

Short, continuous high-intensity exercise (Table 9C) induces a dramatic increase in plasma [K^+^] during exercise, followed by a rapid decline post-exercise (Sjøgaard et al. 1985; Sejersted et al. 1982; Medbø and Sejersted 1985, 1990; Kowalchuk et al. 1988a, b; Paterson et al. 1989; Juel et al. 1990; Hallén and Sejersted 1993). In eight studies utilising exhaustive exercise, [K^+^]_a_ rose from a mean value of 4.1 mM (range 3.8–4.5 mM) at rest to 6.8 mM (range 5.5–8.2 mM) at exhaustion and typically fell to below resting values within 1–6 min after exercise. Four of these studies also reported [K^+^]_fv_ rose from 4.5 mM (3.8–5.4 mM) at rest to 7.0 mM (5.7–8.3 mM) at exhaustion and decreased after exercise to below resting values. These above studies drew the final exercise sample “immediately after”, or ~ 10 to 20 s after exercise and therefore probably underestimated the actual increase in [K^+^]_a_ or [K^+^]_fv_ during exercise. Four studies that determined [K^+^]_a-fv diff_ during exhaustive exercise reported values ranging from − 0.1 to − 1.0 mM at end-exercise, indicating net K^+^ release from contracting muscles and which then reversed to positive values after exercise, indicating net K^+^ movement back into muscle. Finally, arterio-venous [K^+^] measures across inactive forearm muscles revealed a positive [K^+^]_a-acv diff_ of 1.3 mM during intense leg exercise, which indicated that inactive muscles take up K^+^ during exercise (Kowalchuk et al. 1988a). The elevated [K^+^]_a_ was proposed to contribute to the exercise hyperpnea (Paterson 1992, 1996b), most likely mediated via K^+^ effects on the carotid body chemoreceptors (Band and Linton 1986; Linton and Band 1985). Many studies suggested that the large muscle K^+^ release contributed to fatigue, which is discussed elsewhere (Renaud et al. 2023).

#### Intense intermittent exercise

Eight studies during this period (Table 9D) examined plasma [K^+^] during and after intense intermittent exercise and most used 4–5 bouts of exercise of 30–60 s duration, with intervening rest periods of 4–4.5 min and with blood samples “during” exercise being drawn ~ 10 to 15 s after completion of the bout (Costill and Saltin 1975; Katz et al. 1985; Hermansen et al. 1984; Medbø and Sejersted 1990; Lindinger et al. 1990a, 1992; McKelvie et al. 1989, 1991, 1992; Bangsbo et al. 1992a, b). The dramatic oscillations in [K^+^]_a_ first reported with repeated sprint treadmill running (Hermansen et al. 1984) were observed in most studies, with mean [K^+^]_a_ for the five studies that utilised short sprints reaching 6.9, 6.7, 6.2 and 6.0 mM for the first four bouts. A few studies also determined [K^+^]_fv_, with a small positive or zero [K^+^]_a-fv diff_ found at ~ 15 s after exercise in one study (Lindinger et al. 1992), but − 0.6 and − 0.4 mM when measured during two of the four bouts at 100% *V*O_2max_ in another (Katz et al. 1985) (Fig. 7D). An important additional observation from these studies was that [K^+^]_a_ declined below rest or to hypokalaemic values after each sprint bout and in early recovery (Hermansen et al. 1984; Medbø and Sejersted 1990), or from 5 to 90 min post-exercise (McKelvie et al. 1989; Lindinger et al. 1992). Many of these studies focused on the possible role of elevated [K^+^]_e_ in fatigue, whilst others also examined the importance of changes in [K^+^] and other strong ions in acid–base regulation with exercise (Kowalchuk et al. 1988a, b), see (Stickland et al. 2013).

#### Incremental exercise or exercise with combined modalities

Early incremental cycling or treadmill running studies that sampled blood *during* exercise reported a progressive increase in [K^+^]_acv_ to ~ 5 to 6 mM (Wilkerson et al. 1982; Pivarnik et al. 1988), whilst [K^+^]_a_ rose in a concave curve with workrate to ~ 6 to 6.5 mM (Paterson et al. 1990; Vøllestad et al. 1994). The [K^+^]_fv_ determined by a K^+^-selective electrode reached ~ 6.2 to 6.8 mM during submaximal-to-maximal knee extensions or cycling (Hallén and Sejersted 1993; Vøllestad et al. 1994; Hallén et al. 1994) (Fig. 7E). They showed that after an initial brief lag, [K^+^]_fv_ rose rapidly in a manner dependent upon both exercise intensity and duration. During moderate workrates, [K^+^]_fv_ rose initially and then declining slightly, at higher submaximal workrates [K^+^]_fv_ increased and then plateaued, whilst during workrates close to and above *V*O_2max_, [K^+^]_fv_ increased continuously (Hallén and Sejersted 1993; Vøllestad et al. 1994; Hallén et al. 1994). During submaximal workrates, the [K^+^]_a-fv diff_ difference was mostly negative, indicating that K^+^ was released from the contracting muscles throughout most of submaximal exercise. They concluded that muscle K^+^ efflux was dependent on exercise workrate and suggested that muscle NKA activity was insufficient to prevent K^+^ loss, with K^+^ reuptake rate estimated to be only 15–25% of the theoretical maximum K^+^ uptake rate. Finally, during two-legged knee extensor exercise, both [K^+^]_a_ and [K^+^]_fv_ were further increased when incremental arm exercise was superimposed, with K^+^ release into plasma reversed to net K^+^ uptake, indicating that already active muscle could also take up K^+^, possibly due to elevated catecholamines and by inactive fibres (Juel et al. 1999).

#### Muscle interstitial to plasma [K^+^] gradients with exercise

A key question is to what extent plasma [K^+^] is indicative of muscle [K^+^]_int_. Two studies measured [K^+^]_a_, [K^+^]_fv_ and [K^+^]_int_ concomitantly and demonstrated that a large positive gradient exists between muscle [K^+^]_int_ and plasma [K^+^] during exercise (Green et al. 2000; Nielsen et al. 2004). During calf contractions, muscle [K^+^]_int_ was up to 6.5 and 5.8 mM higher than [K^+^]_a_ and popliteal [K^+^]_v_, respectively (Green et al. 2000). During knee extensor exercise, *m. vastus lateralis* [K^+^]_int_ was ~ 5.5 mM higher than [K^+^]_fv_ in the initial minutes of exercise and ~ 3.2 mM higher after 30 min and was ~ 3.9 mM higher at fatigue during incremental exercise, with similar patterns found in the [K^+^]_int_–[K^+^]_a_ gradient (Nielsen et al. 2004). Thus, [K^+^] measurements in arterial or venous plasma substantially underestimate those in the interstitium of contracting muscles.

#### Possible role for red blood cells in K^+^ homeostasis with exercise

Red cells are capable of accumulating K^+^ via NKA activity and Na^+^-K^+^-Cl^−^ co transport and could potentially act as an important transport vehicle for K^+^ released from contracting muscles. Numerous studies therefore investigated the role of erythrocytes in K^+^ homeostasis during exercise, with conflicting findings. Most studies demonstrate that measures of red cell K^+^ were either unchanged or reduced with exercise, although these measures were inconsistent. Early studies reported that arterial erythrocyte [K^+^] ([K^+^]_rbc a_) was unchanged by walking (Kilburn 1966) and that K^+^_rbc a_ content declined during light cycling (Kawakami et al. 1975), whilst femoral venous [K^+^]_rbc_ ([K^+^]_rbc fv_) was unchanged during incremental cycling (Boning et al. 1976). The antecubital venous [K^+^]_rbc_ ([K^+^]_rbc acv_) was unchanged during cycling at 20–60% *V*O_2max_ but fell by 2 mM during exercise at 80% *V*O_2max_ (Hespel et al. 1986a), declined after cross-country running (Hespel et al. 1986b) and by ~ 6 mM after a marathon (Lijnen et al. 1989). The [K^+^]_rbc_ was unchanged during submaximal cycling (Rolett et al. 1990), whilst a small increase in [K^+^]_rbc_ occurred at exhaustion during knee extension exercise due to red cell shrinkage (Juel et al. 1990). During cycling at 110%*V*O_2peak_, K^+^_rbc_ content was unchanged (Vøllestad et al. 1994), during two-legged knee extensor, [K^+^]_rbc a_ and [K^+^]_rbc fv_ were unchanged (Juel et al. 1999) and during exhaustive handgrip exercise, [K^+^]_rbc acv_ and red cell content were unchanged (Maassen et al. 1998). The effects of 4 × 30 s maximal sprint cycling was then determined on [K^+^]_rbc_ derived from [K^+^] in plasma and in whole blood (Lindinger et al. 1990a, 1992; McKelvie et al. 1991, 1992), finding that [K^+^]_rbc a_ was increased at the end of the second and third sprint bouts, with [K^+^]_rbc fv_ unchanged (McKelvie et al. 1991), whilst [K^+^]_rbc a_ was increased during each of 4 × 30 s maximal sprints, with no change found in [K^+^]_rbc acv_ (McKelvie et al. 1992). In summary, most studies reported that measures of K^+^_rbc_ were unchanged during walking, submaximal or maximal cycling, or knee extensor exercise, indicating that erythrocytes did not take up additional K^+^. In contrast, studies utilising intense intermittent sprint exercise found increased [K^+^]_rbc_, suggesting that erythrocytes may contribute to K^+^ homeostasis during such exercise.

#### Possible role for liver in K^+^ homeostasis with exercise

The liver participates in K^+^ homeostasis at rest, with glucose administration inducing liver K^+^ uptake, evidenced in humans by a greater decline in hepatic venous than arterial plasma [K^+^] (Farber et al. 1951). Hepatic vein drains liver, gut and mesentery and thus arterio-hepatic K^+^ differences represent splanchnic K^+^ balance, but K^+^ uptake primarily reflects hepatic K^+^ uptake (Bia and DeFronzo 1981). When insulin was elevated, the liver initially took up K^+^ and accounted for ~ 70% of K^+^ disposal, but after around 1 h, this had returned to zero, or even K^+^ release into the hepatic vein, compensating for hypokalaemia due to peripheral muscle K^+^ uptake (DeFronzo et al. 1980; Alvestrand et al. 1984). In cats, 31% of K^+^ liberated from stimulated muscle was absorbed by the liver (Fenn 1939). However, in humans undertaking submaximal exercise, hepatic venous [K^+^] did not differ greatly from [K^+^]_a_, suggesting the liver did not exert important modulatory effects on [K^+^] during exercise (Linton et al. 1984).

#### Summary of plasma [K^+^] changes with exercise

During the final quarter of the twentieth century, many studies carefully documented plasma [K^+^] responses to exercise in humans in arterial blood and in venous blood draining both active and inactive musculatures and non-muscle tissues (Table 9). The magnitude of increase in plasma [K^+^] with exercise depends on the type of contractions, intensity and duration of exercise, and the blood sampling sites. Intense, brief exercise with a large contracting musculature can increase [K^+^]_a_ to 8 mM, with even higher [K^+^]_fv_ found, followed by extremely rapid decline after exercise. Intense intermittent exercise with relatively short exercise bout durations (30–60 s) yielded dramatic oscillations in [K^+^]_a_ and [K^+^]_fv_, including sustained post-exercise hypokalaemia, whilst submaximal exercise typically induced moderate rises in [K^+^] that might subsequently gradually decline. Most of these studies demonstrated a negative [K^+^]_a-fv diff_ during leg exercise indicating net K^+^ release from the working musculature into plasma, followed post-exercise by a positive [K^+^]_a-fv diff_ indicating net K^+^ re-uptake by previously active muscle, whilst inactive muscles took up K^+^ during exercise. Most studies showed that erythrocytes do not play an important role in K^+^ regulation during exercise, with the exception being intense intermittent exercise, where increases in [K^+^]_rbc_ were found.

### Plasma [K^+^] changes in an applied sport context

The vast majority of studies during the twentieth century focused on K^+^ regulation during laboratory exercise modes, such as cycling, knee extension, running, or forearm contractions, enabling precise measures of exercise intensity, duration and mode in a controlled laboratory environment. However, far fewer studies have investigated K^+^ regulation during an applied sport setting, or even simulated sport activity, with limiting factors including the rapidity of K^+^ regulation together with difficulties of repeated blood and tissue sampling for [K^+^] determinations.

#### Intermittent sports: football (soccer) and squash

Plasma [K^+^] has not been studied extensively during intense intermittent team sports, but is elevated during football (soccer). During friendly matches in Danish Division 4 soccer players, [K^+^]_v_ collected within 30 s of play was moderately increased at various time points during the match, with a peak of 5.1 mM (Krustrup et al. 2006). More detailed analyses of [K^+^] have been undertaken during the intermittent Yo–Yo test, designed to replicate intense running patterns in soccer, where [K^+^]_acv_ peaked at 7.0 mM at exhaustion and fell to 3.7 mM in recovery, similar to exhaustive incremental treadmill exercise (Krustrup et al. 2003). During a modified Yo-Yo test, [K^+^]_acv_ peaked at ~ 5.8 to 6.1 mM, fell post-exercise to ~ 3.5 to 3.7 mM, comparable to peak [K^+^]_acv_ with repeated sprints during sprint-endurance, and sprint training sessions (Mohr et al. 2006; Krustrup et al. 2015). The short duration and intermittent nature of high intensity efforts, with considerable low-intensity recovery periods each would constrain [K^+^]_v_ during soccer, whilst both the arm venous sampling and post-exercise sampling delays mean [K^+^]_v_ would substantially underestimate both [K^+^]_a_ and [K^+^]_fv_. Furthermore, the large gradients between [K^+^]_a_, [K^+^]_fv_ and [K^+^]_int_ found in continuous exercise (Sect. Muscle interstitial to plasma [K^+^] gradients with exercise) suggest that muscle [K^+^]_int_ may also be high during soccer and play a role in fatigue, but this remains to be shown.

During another intermittent sport, squash, forearm [K^+^]_sv_ increased only slightly from 3.8 mM at rest to 4.3 mM at end-exercise, but fell to 3.4 mM at 3 min post-exercise (Struthers et al. 1988), with similar reductions in [K^+^]_sv_ found 5 min post-match to 3.2 mM (Brady et al. 1989) and 3.5–3.6 mM (Lynch et al. 1992). Whilst hyperkalaemia was ruled out as a mechanism of sudden death associated with match-play (Northcote et al. 1986; Struthers et al. 1988), ventricular arrhythmias were often detected after match play (Northcote et al. 1983; Brady et al. 1989). Whilst in healthy individuals during intense exercise, the combined detrimental effects of elevated [K^+^]_a_ and acidosis on the heart are likely offset by the elevation in catecholamines (Paterson 1996a), post-exercise hypokalaemia after squash may increase the risk of arrhythmias. The small increase in [K^+^]_sv_ immediately after squash likely substantially underestimated [K^+^]_a_ during the match, due to blood sampling post-exercise and from a sub-optimal sampling site, whilst the intermittent nature of exercise with only brief sprints also likely limited the exercise-induced [K^+^] increase.

#### Continuous sports: rowing and cross country skiing

The impact of two continuous sports on [K^+^] are contrasted, rowing, which comprises intense exercise conducted over minutes and cross country skiing, which comprises exercise prolonged over many hours. Both utilise a large contracting muscle mass during exercise, capable of releasing K^+^ into plasma with a smaller inactive muscle mass available to clear K^+^ from plasma, and involve both upper and lower limbs. During 2000 m rowing in recreationally active participants, [K^+^]_a_ peaked after 90 s at 6.1 mM and was then sustained at this high level throughout ~ 7 min exercise, despite fatigue indicated by declines in both power output and EMG average median frequency (Atanasovska et al. 2014). In recovery, [K^+^]_a_ fell below rest by 1 min, reached a nadir of 3.3 mM and remained low for 20 min. During “all-out” rowing for 3 min, [K^+^]_a_ rose to ~ 7 mM after 60 s and then remained ~ constant, fell rapidly post-exercise to ~ 3.3 mM after 5 min and remained below baseline for 60 min (Atanasovska et al. 2018). No studies have detailed [K^+^] dynamics in elite rowers to ascertain whether greater K^+^ shifts are elicited. Prolongation of the cardiac QT interval and T wave peak-to-end interval after exercise were related to [K^+^]_a_, suggesting the possibility of vulnerability to arrhythmias (Atanasovska et al. 2018; Tran et al. 2022). In contrast to rowing, cross country skiing, for traditional events, is usually for much longer duration and at a lower intensity, with only modest [K^+^] found “immediately after” skiing, although the timing of sampling is unclear. Studies that reported long delays in blood sampling post-exercise are here ignored. After an 85 km, “Vasaloppet” cross country skiing race over 5–8.5 h, which included 2 world champion participants, [K^+^]_acv_ increased to 5.3 mM (Åstrand and Saltin 1964), although after a 70 km cross country ski race of 4.4–6.5 h duration, serum [K^+^]_v_ of only 4.7 mM was found (Refsum and Strömme 1975). More recently, during intense double pole skiing (upper body exercise) on a modified rowing ergometer, [K^+^]_a_ and [K^+^]_fv_ both rose to 5.4 mM and subclavian [K^+^]_v_ to 5.8 mM, with K^+^ released from the contracting arm muscles ([K^+^]_a-v diff_ ~ − 1.2 mmol.min^−1^) (Rud et al. 2014).

## Specific intervention effects on plasma [K^+^] with exercise, linked with perturbations in muscle NKA activity

Studies in humans since the 1990s increasingly investigated the effects of different interventions that potentially involve effects via NKA activity in muscle, on plasma [K^+^] responses to exercise and assessed implications for fatigue, including the NKA inhibitor digoxin, acid–base manipulations, glucose/carbohydrate intake, muscle glycogen depletion, training and inactivity, β-adrenergic antagonists/agonists, as well as antioxidants. The following section briefly cites key historical intervention studies, then focusing mainly on studies from late in the previous century to the present. Although not exhaustive, this nonetheless indicates the tremendous recent growth in research and enhanced understanding of the complexity of K^+^ regulation with exercise and their implications for fatigue.

### Digoxin effects on plasma [K^+^] and exercise

Digoxin, a specific NKA inhibitor, is used to treat patients with atrial fibrillation or severe heart failure (Bavendiek et al. 2017) to improve cardiac output (Levi et al. 1994), but potentially impairs exercise performance due to NKA inhibition and ensuing high [K^+^]. The first evidence that digoxin affected [K^+^] during exercise was in patients with atrial fibrillation, where 2.5 nM serum digoxin induced an ~ 0.4 mM higher serum [K^+^]_acv_ during submaximal cycling than with zero digoxin (Nørgaard et al. 1991). Similarly, in patients with heart failure, digoxin elevated [K^+^]_fv_ by ~ 0.1 to 0.3 mM during and after submaximal and incremental cycling and increased muscle K^+^ release during exercise by 138% (Schmidt et al. 1995). They also reported a 9% digoxin occupancy of muscle NKA and a ~ 18% lower ouabain-binding site content with digoxin than in healthy controls. Three studies have investigated digoxin effects on [K^+^] in healthy individuals. Whilst resting serum [K^+^]_acv_ was elevated by ~ 0.2 mM after 10 day oral digoxin (Edner et al. 1993), no effects of digoxin were found after intravenous digoxin infusion on [K^+^]_acv_ after 3 min handgrip exercise (Janssen et al. 2009), or after 14 d oral digoxin on [K^+^] or K^+^ fluxes during and after either finger flexion or leg cycling (Sostaric et al. 2022), with time to fatigue unchanged during both latter studies. Thus, healthy individuals taking digoxin had no major effects on [K^+^] with exercise, or on exercise performance. Acute experimental approaches to reduce functional muscle NKA are required to ascertain possible effects on K^+^ regulation and fatigue.

### Acid–base manipulation effects on plasma [K^+^] and exercise

Alkalosis (10 g sodium bicarbonate, NaHCO_3_) increased exercise duration, which was reduced by acidosis (15 g ammonium chloride, NH_4_Cl) (Dennig et al. 1931), as later confirmed (Jones et al. 1977) and now with strong evidence that alkalosis induced by NaHCO_3_ ingestion can enhance performance during intense exercise of 0.5–12 min duration (Grgic et al. 2021). A role of alkalosis in K^+^ regulation was indicated by reports of low [K^+^]_acv_ in patients with alkalosis (Farber et al. 1951). Several studies subsequently investigated alkalosis or acidosis effects on plasma [K^+^] with exercise and performance in humans. Prior acidosis induced higher early K^+^ release and reduced time to fatigue by 26% during knee extensor exercise (Bangsbo et al. 1996). NaHCO_3_ ingestion reduced [K^+^]_acv_ by ~ 0.3 to 0.6 mM and increased time to fatigue by ~ 12% during wrist flexion exercise (Raymer et al. 2004), lowered [K^+^]_a_ and [K^+^]_acv_, and prolonged time to fatigue by ~ 25% during finger flexion contractions (Sostaric et al. 2006), but did not lower [K^+^]_v_ during submaximal cycling (Stephens et al. 2002) or [K^+^]_acv_ during an intermittent Yo–Yo test performance (Krustrup et al. 2015), whilst alkalosis induced by sodium citrate lowered *m. vastus lateralis* [K^+^]_int_ during knee extensor exercise by ~ 1.5 to 3 mM, but did not affect [K^+^]_v_ (Street et al. 2005). Experimental manipulation of alkalosis in rats reduced the loss of muscle intracellular K^+^ in stimulated muscles (Lindinger et al. 1990b), but elevated HCO_3_^−^ did not affect K^+^ efflux during *m. soleus* contractions (Broch-Lips et al. 2007). Thus, ergogenic effects of NaHCO_3_ typically found in humans during intense exercise of short duration are not always associated with K^+^-lowering and the suggested links between alkalosis and increased muscle NKA activity or K^+^ channel deactivation remain to be convincingly proven.

### Insulin, glucose and glycogen effects on plasma [K^+^] and exercise

The K^+^-lowering effects of insulin were first shown in humans around one century ago, where insulin injection lowered serum [K^+^]_v_ from 4.5 to 3.2 mM in diabetic patients (Harrop 1924), to 1.8 mM in an untreated diabetic patient (Kerr 1928) and consequently used as “*insulin shock therapy*” to lower [K^+^]_v_ in patients with schizophrenia (Keys 1938a). An important link with muscle function was seen after treatment with insulin, NaHCO_3_ and glucose which lowered [K^+^] to 2.5 mM (Holler 1946), whilst oral glucose and intravenous glucose infusion in “normal subjects” reduced [K^+^]_a_ by 0.4 mM and doubled the [K^+^]_a-acv diff_ (Farber et al. 1951). Insulin infusion was later found not to affect [K^+^]_a_, to lower [K^+^]_acv_ and widen the [K^+^]_a-acv diff_ to ~ 0.35 mM, demonstrating K^+^ uptake by forearm muscle (Andres et al. 1962), with similar muscle K^+^ uptake evident at ninefold lower insulin infusion (Zierler and Rabinowitz 1964). A standard oral glucose tolerance test elevated insulin, lowered both [K^+^]_a_ and [K^+^]_acv_ and increased the [K^+^]_a-acv diff_ during and after intense intermittent cycling exercise (Steward et al. 2021). This K^+^-lowering with leg exercise was probably due to insulin increasing muscle NKA activity and thus also K^+^ uptake in inactive forearm muscles.

Prolonged exercise lowered muscle K^+^_c_ as muscle glycogen was consumed (Ahlborg et al. 1967; Bergström and Hultman 1966), suggesting a possible link between muscle glycogen content and K^+^ homeostasis. The effects of elevated muscle glycogen content on muscle K^+^ release was examined during intense one-legged exercise leading to exhaustion (~ 3 min), comparing exercise with normal glycogen content in one leg versus the other where glycogen content was ~ doubled, finding 14% greater muscle K^+^ release in the high- than normal-glycogen leg in an initial bout (Bangsbo et al. 1992b). The opposite effect of muscle glycogen depletion increased both [K^+^]_a_ and [K^+^]_fv_ during cycling to exhaustion at 75% *V*O_2peak_, with a more negative [K^+^]_a-fv diff_ early in exercise, that later reversed to a net K^+^ uptake by contracting muscles (Lindinger et al. 1994). The cessation of net muscle K^+^ efflux after the first 15 min of exercise in glycogen depletion suggested greater muscle NKA activation. Muscle NKA depends on ATP from glucose derived from glycogenolytic or glycolytic sources (Jensen et al. 2020) and has its own sub-sarcolemmal pool of glycogen (Nielsen et al. 2022), consistent with a link between modulation of muscle glycogen, NKA activity and K^+^ regulation during exercise. Thus, elevated insulin and glucose at rest promote K^+^ entry into muscle and liver and lower [K^+^], whilst glucose ingestion lowers [K^+^] during exercise and induces K^+^ uptake by inactive muscle. Manipulation of muscle glycogen also modulates muscle K^+^ release and [K^+^] during intense exercise, and the [K^+^]_a-fv_ during prolonged exercise, but further work is required to understand the mechanisms underlying these effects.

### Caffeine effects on plasma [K^+^] and exercise

Caffeine has long been known to enhance performance during prolonged exercise (Ivy et al. 1979; Costill et al. 1978; Graham and Spriet 1991; Grgic et al. 2020) and to elevate circulating adrenaline (Graham and Spriet 1991). However, despite the well-known adrenergic stimulation of NKA activity in animal muscle (Clausen and Flatman 1977; Flatman and Clausen 1979) and plasma K^+^-lowering effects in humans (“Adrenaline, β-adrenergic agonists and antagonists and plasma [K+] with exercise”), only a few studies have investigated caffeine effects on plasma [K^+^] during exercise in humans. Caffeine (9 mg kg^−1^) elevated plasma adrenaline and lowered [K^+^]_acv_ by ~ 0.4 mM during cycling and by ~ 0.7 mM during running at 78–85% *V*O_2peak_ (Lindinger et al. 1993) and (6 mg.kg^−1^) increased arterial plasma adrenaline and noradrenaline and lowered plasma [K^+^]_a_ whilst cycling at 70% *V*O_2max_ (Graham et al. 2000) and lowered muscle [K^+^]_int_ by 1.8 mM during knee extensor exercise (Mohr et al. 2011). In contrast, caffeine (6 mg kg^−1^) did not affect peak [K^+^]_acv_ during a Yo–Yo intermittent recovery test (Mohr et al. 2011). Thus, caffeine likely modulates K^+^ homeostasis during exercise, probably due to elevated adrenaline and/or caffeine metabolites stimulating increased muscle NKA activity and inducing K^+^-lowering.

### Training and reduced activity effects on plasma [K^+^] with exercise

As training characteristics and their physiological consequences and performance benefits vary, the effects on plasma [K^+^] during exercise are considered separately for each of endurance, sprint interval and speed-endurance interval training (McKenna 1995; McKenna et al. 1996).

#### Endurance training

An early seminal study demonstrated that prolonged and interval running training for ~ 2 months (after 20 d prior bedrest), increased *V*O_2max_ and also each of peak incremental exercise [K^+^]_a,_ [K^+^]_fv_ and [K^+^]_acv_, by 0.1, 0.4 and 0.5 mM, respectively (Saltin et al. 1968). After training, a more negative [K^+^]_a-fv diff_ (− 0.05 vs − 0.2 mM) suggested greater leg muscle K^+^ release, whilst a narrower [K^+^]_a-acv diff_ (0.9 vs 0.5 mM) suggested less forearm muscle K^+^ uptake than in control. In an early cross-sectional comparison, lower [K^+^]_fv_ during incremental cycle ergometer exercise was seen at equivalent workrates in endurance trained than in untrained individuals (Tibes et al. 1974), with K^+^-lowering effects of endurance training later confirmed in longitudinal training studies, as reviewed previously (McKenna 1995). Thus, endurance training for 10 weeks lowered [K^+^]_acv_ by ~ 0.2 to 0.5 mM during submaximal and by 0.5 mM at peak incremental cycling exercise (Kjeldsen et al. 1990), whilst after 6 consecutive days, arterialised [K^+^]_v_ was reduced during submaximal cycling where workrates and times were ~ matched before and after training (Green et al. 1993).

#### Sprint and intense interval training

Studies also reveal that sprint training enhances K^+^ regulation with exercise. Seven weeks of sprint training reduced arterialised [K^+^]_v_ by 0.2 mM during maximal intermittent cycle sprint bouts after correcting for fluid shifts (McKenna et al. 1993), by ~ 0.6 mM during cycling at 130% pre-train *V*O_2peak_ (Harmer et al. 2000) and lowered both [K^+^]_a_ and [K^+^]_fv_ by 0.2–0.3 mM after a maximal 30 s cycle sprint (McKenna et al. 1997). Similarly, intense intermittent training of the knee extensors lowered [K^+^]_fv_ during submaximal and incremental exercise (Nielsen et al. 2004), although intense interval training in older participants did not modify peak [K^+^]_acv_ during incremental cycling (Wyckelsma et al. 2017). High-intensity interval training also reduced [K^+^]_fv_ and K^+^ release rate during high intensity exercise and increased K^+^ uptake rate in recovery (Hostrup et al. 2023), whilst training increased incremental peak work rate and lowered [K^+^]_fv_ during low and high intensity exercise in one leg and additionally in a blood flow restricted leg, and also reduced the [K^+^]_a-fv diff_ and the K^+^ release at high workrate (Christensen and Bangsbo 2019). Several other studies demonstrated that high-intensity interval training enhanced K^+^ regulation during intense exercise, evidenced by ~ 0.3 to 0.6 mM lower [K^+^]_acv_ after exhaustive treadmill runs, 0.7—0.8 mM lower [K^+^]_fv_ after an exhaustive cycling bout and 0.3 mM lower [K^+^]_fv_ during submaximal knee extensor exercise (Iaia et al. 2008; Bangsbo et al. 2009; Gunnarsson et al. 2013; Lemminger et al. 2022). Thus, the majority of evidence points to enhanced K^+^ regulation during exercise after intense interval training.

#### Inactivity and bedrest

Few studies have investigated the effects of reduced physical activity on [K^+^] during exercise. After 20 day bedrest, the peak incremental exercise [K^+^]_a_, [K^+^]_fv_, [K^+^]_acv_ and *V*O_2max_ each declined, by 0.7, 0.5 and 0.4 mM and by 26%, respectively (Saltin et al. 1968). During submaximal exercise, [K^+^]_a_, [K^+^]_fv_ and [K^+^]_acv_ were similarly reduced after bedrest, reflecting lower absolute workrates, indicating that [K^+^] remained similar for a given *V*O_2_. Similar [K^+^]_acv_ were found during submaximal cycling before and after 4 weeks of detraining in endurance athletes (Madsen et al. 1993) and after inactivity via 23 d unilateral lower limb suspension (Perry et al. 2016), despite shorter time to fatigue in both studies.

#### Summary and implications of training and reduced activity effects on plasma [K^+^] with exercise

In summary, a characteristic finding in many training studies is a lowering of circulating [K^+^] during and after intense exercise, but this did not always occur. First, this is dependent on appropriate comparisons between workrates, intensities or durations matched before and after training (McKenna 1995; McKenna et al. 1996). Second, in many instances, [K^+^]_acv_ was measured during leg exercise, where measures of [K^+^]_fv_ or of systemic changes via [K^+^]_a_ would be more appropriate for determining [K^+^] changes with training. Lowering of circulating [K^+^] during exercise after training is consistent with the lower [K^+^]_int_ during exercise after training (Sect. Human skeletal muscle interstitial [K^+^] with exercise) and also generally consistent with the 8–22% increase found in muscle NKA_c_ (“Effects of training, inactivity and aging on muscle [3H]-ouabain-binding site content”), although typically only weak or even no association was reported after training between the plasma [K^+^] variables and muscle NKA_c_ (McKenna 1995; McKenna et al. 1996). In addition to lowered plasma [K^+^] and increased muscle NKA_c_ after training, adaptations also include lower [K^+^]_int_ during exercise and hyperpolarised *E*_m_ (Knochel et al. 1985), indicating an overall improvement in K^+^ homeostasis. Studies examining the effects of reduced physical activity utilising bedrest, detraining or unilateral limb suspension on [K^+^] during exercise have also shown varying findings, with only bedrest inducing reduced [K^+^] during exercise. Further work is required to ascertain these effects and their functional sequalae.

### Adrenaline, β-adrenergic agonists and antagonists and plasma [K^+^] with exercise

Β-adrenergic activation is a potent activator of muscle NKA (Cairns and Borrani 2015) and increased circulating catecholamines during exercise due to elevated sympathetic activity (Kjaer 1989) could likely influence systemic [K^+^] during exercise. Whilst studies early in the twentieth century examined the direct impacts of adrenaline injection on circulating [K^+^] in resting humans, many studies since the 1970s have utilised either β-blockade or β-stimulation to examine the role of β-adrenergic regulation in K^+^ homeostasis with exercise and its implications for fatigue.

#### Adrenaline injection

Intravenous adrenaline injection decreased serum [K^+^] within a few minutes by 4–15% below rest (Keys 1937) and in healthy men caused “a consistent and marked drop in the K immediately…”, with [K^+^]_acv_ lowered by ~ 0.3 mM from rest after 0.4 min, remaining low for 25 min, with a slight rise after 40–60 min and with little effect on [K^+^]_rbc_ (Keys 1938b).

#### β-Adrenergic blockade

The effects of β-adrenergic blockade on K^+^ during and after exercise were first studied using the non-specific β-blocker propranolol and the β_1_-specific blocker metoprolol delivered orally, finding each increased [K^+^]_acv_ by ~ 0.4 to 0.5 mM during submaximal exercise and slightly in recovery (Carlsson et al. 1978). Subsequent studies using intravenous propranolol interventions revealed that β-adrenergic blockade elevates plasma [K^+^]_a_, [K^+^]_fv_ or [K^+^]_acv_ during a wide range of different exercise types, with several, but not all studies demonstrating an increased K^+^ release from contracting muscles, as well as a reduced K^+^ uptake by other tissues and with these thought to be mediated by adrenergic inhibition of muscle NKA (Williams et al. 1985; Katz et al. 1985; Hallén et al. 1994; Gullestad et al. 1995).

#### β-Adrenergic agonists

The β_2_-specific adrenoreceptor agonist salbutamol was used to treat patients with hyperkalaemic periodic paralysis, substantially reducing the unusual post-exercise increase in [K^+^]_acv_ found in these patients and alleviating the associated muscle weakness and paralysis (Wang and Clausen 1976). Several studies then investigated the effects of β_2_-agonists terbutaline and salbutamol on K^+^ dynamics and exercise performance in healthy individuals. Intravenous terbutaline infusion lowered both [K^+^]_a_ and [K^+^]_fv_ by ~ 0.8 to 0.9 mM during knee extensor contractions, with conflicting findings on K^+^ release by the active leg (Rolett et al. 1990; Hallén et al. 1996). Terbutaline or salbutamol inhalation lowered [K^+^]_acv_ or induced a smaller rise in [K^+^]_a_ (~ 0.17 mM), after exhaustive sprints, during and after submaximal as well as intense intermittent cycling (Hostrup et al. 2014a, 2016; Altarawneh et al. 2016). The effects of β_2_-agonists on lowered [K^+^] during and after exercise are likely due to increased activation of muscle NKA (Clausen and Flatman 1977), mediated via cAMP and PKA pathways (Cairns and Borrani 2015).

### Antioxidant status and plasma [K^+^] with exercise

The NKA is redox-sensitive in many cell types, with NKA activity inhibited by numerous reactive oxygen species and this alleviated by different antioxidants (McKenna et al. 2006). Hence, there is recent interest in whether interventions altering redox status can also modulate systemic [K^+^] during exercise and affect performance. Several studies examined the effects of intravenous infusion of the non-specific antioxidant, *N*-acetyl cysteine (NAC) on arterialised [K^+^]_sv_ and on exercise performance, which yielded varying conclusions (Medved et al. 2003, 2004; McKenna et al. 2006). It was first found that NAC did not affect [K^+^]_sv_ during 4 bouts of cycling at 130%*V*O_2peak_, but did cause a greater rise in [K^+^]_sv_ above rest in the final bout continued to exhaustion, without affecting performance time (Medved et al. 2003). Similarly, NAC did not change [K^+^]_sv_ during 45 min cycling at 70% *V*O_2peak_, but in contrast, reduced the rise in [K^+^]_sv_ when continued to fatigue at 90% *V*O_2peak_, also without affecting time to fatigue (Medved et al. 2004). However, in a subsequent study, NAC increased performance time (24%), the rise in [K^+^]_v_ at fatigue and the rise in [K^+^]_sv_ corrected for work done during prolonged cycling including to fatigue at 92% *V*O_2peak_, as well as attenuating the decline in muscle 3-*O*-MFPase activity at fatigue (McKenna et al. 2006). These studies thus gave contradictory conclusions on NAC effects on K^+^ regulation during exercise, but were limited by not directly measuring [K^+^]_a_ or [K^+^]_fv_. More recently, NAC did not affect [K^+^]_a_, [K^+^]_fv_, the [K^+^]_a-fv diff_ or muscle K^+^ release during knee extension exercise, with some additional affects noted in either a blood flow restricted leg, or after intense intermittent training (Christiansen et al. 2019; Lemminger et al. 2022). Finally, in well-trained cyclists, oral NAC supplementation did not change either [K^+^]_acv_ or mean power during exhaustive cycling (Christensen and Bangsbo 2019). In summary, altering redox state using the antioxidant NAC has to date yielded inconsistent effects on [K^+^] and time to fatigue during prolonged and intense exercise, with further research required to clarify these inconsistencies.

## Sex and sample size limitations in plasma [K^+^] changes with exercise

### A century of studying exercise responses in male participants

**Fig. 8:**
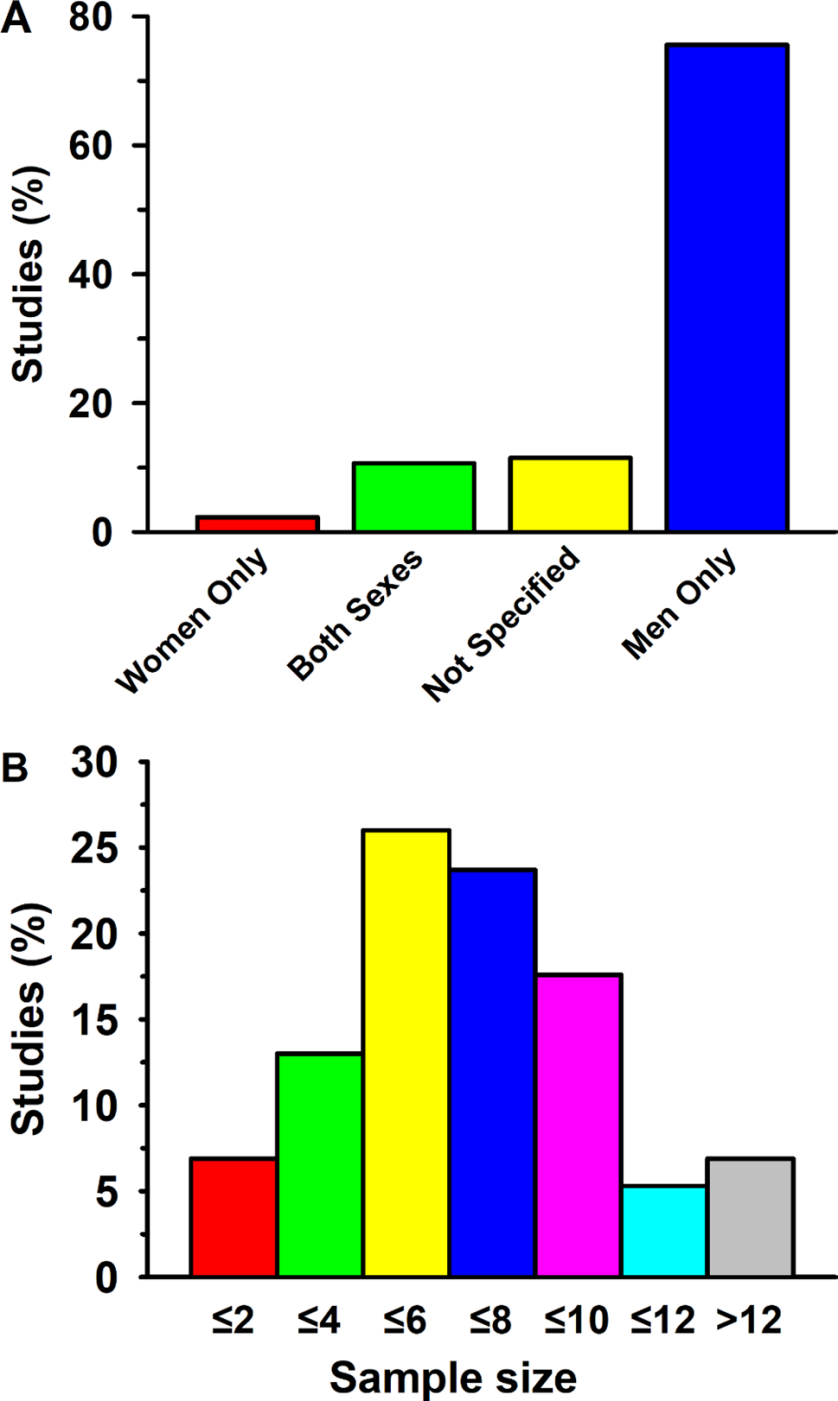
Histograms of participant characteristics from human exercise and plasma [K^+^] studies for **A**) sex and **B**) number of participants

A striking limitation of studies conducted during the twentieth century and including the first 2 decades in the twenty-first century was that they almost exclusively studied physiological responses in males. Of the 131 studies (and including sub-studies) cited above on plasma [K^+^] and exercise, 113 (85%) included male participants, 99 comprised males only, 18 included female participants, and only 3 studies (2%) comprised females only (Fig. 8). This review does not cite every study published during this period, so these statistics are not fully inclusive but are, nonetheless, striking. Hence, the past century was largely spent studying plasma [K^+^] and exercise in men and therefore most of this literature should be relabeled as physiological studies in men. Past historical, sexist approaches to science have excluded women and rectifying this is a major challenge, but one that is relatively simple to address for future studies.

#### Women only studies: plasma [K^+^] and exercise

The three studies cited over the past century that investigated plasma [K^+^] and exercise in women only, appear to show similar responses to men, although this has never been systematically examined. During incremental cycle ergometer exercise to exhaustion in 6 women, [K^+^]_a_ rose from 4.2 at rest to 6.4 mM at exhaustion, [K^+^]_fv_ from 4.3 to 6.8 mM, with both declining rapidly at 4 min post-exercise to 3.8 and 3.7 mM, respectively (Hallén et al. 1994). During incremental treadmill running in 29 women, [K^+^]_a_ rose from ~ 4.2 mM at rest to a peak of ~ 6.4 mM (McClaran et al. 1998). During three, 5 min intense cycle ergometer bouts in 14 women, [K^+^]_a_ rose from ~ 4 mM pre-exercise to 5.3, 5.1 and 5.1 mM, respectively, and declined at 5 min recovery [K^+^]_a_ to ~ 3.6 mM (Zavorsky et al. 2007). Further studies on women, [K^+^] and exercise are clearly required.

### A century of studying too few participants

A second striking observation was the propensity for utilising a very small sample size in most studies. Of these 131 cited studies (and including sub-studies), 26 utilised only *n* ≤ 4 participants, nearly half were conducted utilising *n* ≤ 6, and almost two-thirds used *n* ≤ 8 participants, whilst only 9 (7%) used *n* ≥ 12 participants. Therefore, around one-half to two-thirds of studies conducted over the past century utilised a small-to-very small sample size and consequently, many may have had an increased risk of Type II error and have failed to detect at least some subtleties in the plasma [K^+^] responses to exercise and/or in the efficacy of the intervention used. Hence, many conclusions that have been accepted may well be subject to further scrutiny in studies with higher statistical power. In addition, some early and well-accepted findings such as changes in [K^+^]_a-fv diff_ during different submaximal exercise protocols were obtained from two subgroups each with only three participants (Sjøgaard et al. 1985), whilst small sample sizes were routine in studies investigating muscle NKA and exercise. There is value in small-n studies in special situations and this will continue to apply to human exercise studies (Ploutz-Snyder et al. 2014). Nonetheless, future studies should include a larger sample size based on prior statistical power calculations (and report this) and also include a sufficient number of participants of both sexes to enable more inclusive physiological interpretations. Possible sex-based differences in plasma [K^+^], muscle NKA, exercise and training should also be explored.

## Summary

This review covered the huge historical developments in each of the broad fields of skeletal muscle Na^+^ and K^+^ contents, concentrations and fluxes; muscle NKA activity, content and isoforms; and plasma [K^+^] during muscle contractions and exercise (Figs. 2, 4, 6). The compounding growth in knowledge over this past century in each of these fields serves as a platform for the next. The resulting impacts of this research progressed from discovery and understanding of basic mechanisms, through to uncovering the intricacies of their regulation *in-vitro* and *in-vivo*, then to understanding their integration with multiple proteins, local factors, endocrine systems and organs, to applications such as, e.g., understanding their roles in muscle function, fatigue and performance, and to understanding their collective impacts in health and disease.

## Data Availability

No data statement is required as this is a review and cites published data. No new original data is included other than that in Figure 8 but this is descriptive only.

## Abbreviations

AP: Action potential
acv: Antecubital, or deep venous forearm plasma
*K*_m_, *K*_0.5_: Apparent affinity
a: Arterial plasma
a-acv diff: Arterio-venous difference in plasma [ion] across the forearm
a-fv diff: Arterio-venous differences in plasma [ion] across the leg
Cl^−^: Chloride ion
Cl^–^_c_: Cl^−^ content
cAMP: Cyclic AMP
PKA: cAMP-dependent protein kinase
DNA: Deoxyribonucleic acid
rbc: Erythrocyte
E_m_: Membrane potential
EDL: Extensor digitorum longus
fv: Femoral venous plasma
α_1_^+/−^, α_2_^+/−^: Gene-targeted heterozygous mice lacking one copy of the α_1_ or the α_2_ isoform
α_2_^−^^/^^−^: Gene-targeted mice lacking both copies of the α_2_ isoform (α_2_ knockout)
skα_2_^−^^/^^−^: Gene-targeted mice lacking both copies of the α_2_ isoform specifically in skeletal muscle (skeletal muscle α_2_ knockout)
H^+^: Hydrogen ion
int: Interstitial
i: Intracellular
*p*-NPPase: K^+^-activated *p*-nitrophenyl phosphatase assay
3-*O*-MFPase: K^+^-stimulated 3-*O*-methyl fluorescein phosphatase assay
*E*_m_: Membrane potential
mRNA: Messenger ribonucleic acid
*m.*: Muscle
NKA: Na^+^, K^+^-ATPase
α_1_, α_2_, α_3_: Na^+^, K^+^-ATPase alpha subunit 1, 2 and 3 isoforms
β_1_, β_2_, β_3_: Na^+^, K^+^-ATPase beta subunit 1, 2 and 3 isoforms
FXYD1: Phospholemman, member of FXYD family of proteins associated with NKA
[K^+^]: Potassium concentration
K^+^_c_: Potassium content
K^+^: Potassium ion
^42^K: Radioactive potassium
^86^Rb: Radioactive rubidium
^22^Na, ^24^Na: Radioactive sodium
Rb^+^: Rubidium ion
SR: Sarcoplasmic reticulum
SET: Speed-endurance training
[Na^+^]: Sodium concentration
Na^+^_c_: Sodium content
Na^+^: Sodium ion
sv: Superficial venous plasma
t-tubule: Transverse tubule
[^3^H]-ouabain: Tritiated ouabain
VO_4_: Vanadate
v: Venous plasma from unspecified site
Nav: Voltage-gated Na^+^ channel

## Footnotes

1 In this review, the dispersion of results around a mean uses the standard deviation (reported or calculated).
